# Electrochemical Dehydration of Sulfonic Acids to Their Anhydrides

**DOI:** 10.1021/acs.joc.5c01155

**Published:** 2025-08-22

**Authors:** Enrico Lunghi, Annemijn M. van Koten, Johannes Schneider, Siegfried R. Waldvogel

**Affiliations:** a 28313 Max-Planck-Institute for Chemical Energy Conversion (MPI CEC), Stiftstraße 34−36, Mülheim an der Ruhr 45470, Germany; b Department of Chemistry, 9182 Johannes Gutenberg University (JGU), Duesbergweg 10-14, Mainz 55128, Germany; c Institute of Biological and Chemical Systems − Functional Molecular Systems (IBCS FMS), Karlsruhe Institute of Technology (KIT), Kaiserstraße 12, Karlsruhe 76131, Germany

## Abstract

We developed an electrochemical method for sulfonic anhydride synthesis and one-pot derivatization into sulfonamides and mesylates under mild conditions. Aliphatic sulfonic anhydrides were electrogenerated in up to a 74% yield. Since isolating these moisture-sensitive intermediates is challenging, the in situ-generated sulfonic anhydrides were directly converted into sulfonamides or alkyl sulfonates using amines or alcohols, respectively. This scalable process enables the synthesis of various nitrogen- and oxygen-based products in the multigram range.

## Graphical abstract

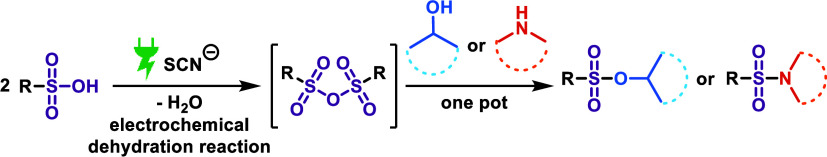

## Introduction

The sulfonic anhydride functionality represents a very versatile structural motif in organic synthesis, as it serves as a key intermediate in the preparation of highly value-added compounds.^1^ The simplest representative is methanesulfonic anhydride, which is a widely used reagent from academic to industrial scale.^2^ Sulfonic anhydrides enable the direct preparation of sulfonamides, sulfonates, and other sulfonyl-containing compounds, which are prevalent in pharmaceuticals (Scheme 1), agrochemicals, and materials science.^3^ Because of their electrophilic nature, sulfonic anhydrides facilitate efficient and selective transformations of various nucleophiles under mild reaction conditions, making them indispensable reagents in contemporary synthetic chemistry. Despite their broad usefulness, conventional methods for the synthesis of sulfonic anhydrides often rely on harsh dehydrating agents such as phosphorus pentoxide or thionyl chloride,^4^ leading to toxic waste and operational hazards.^5^ Sulfur-based functionalization strategies typically rely on prefunctionalized sulfonyl halides, which are often expensive, difficult to store due to their moisture sensitivity, and not widely commercially available.^6^ In contrast, sulfonic acids are inexpensive, widely available, and environmentally benign starting materials, making their direct electrochemical valorization highly desirable. The electrochemical dehydration of sulfonic acids to sulfonic anhydrides therefore represents an interesting alternative to conventional dehydration reactions.

**Scheme 1:**
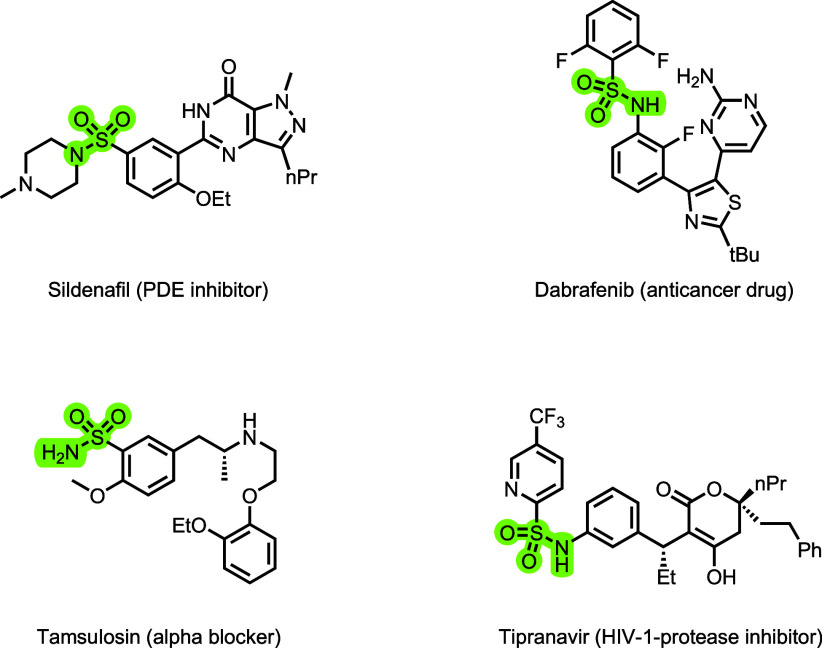
Examples of Active Pharmaceutical Ingredients (APIs) that Contain a Sulfonamide Structural Motif

The field of electrosynthesis is currently experiencing a remarkable and ongoing renaissance,^7^ because electrochemical reactions offer a variety of advantages over conventional transformations.^8^ Electrochemical reactions are characterized by mild reaction conditions.^9^ Electricity, preferably originating from renewable energy sources, is utilized as a comparably inexpensive and universal redox agent, which helps to minimize reagent waste.^10^ These and further advantages are the reason electrosynthesis is establishing itself as a 21st-century technique.^11^

**Scheme 2:**
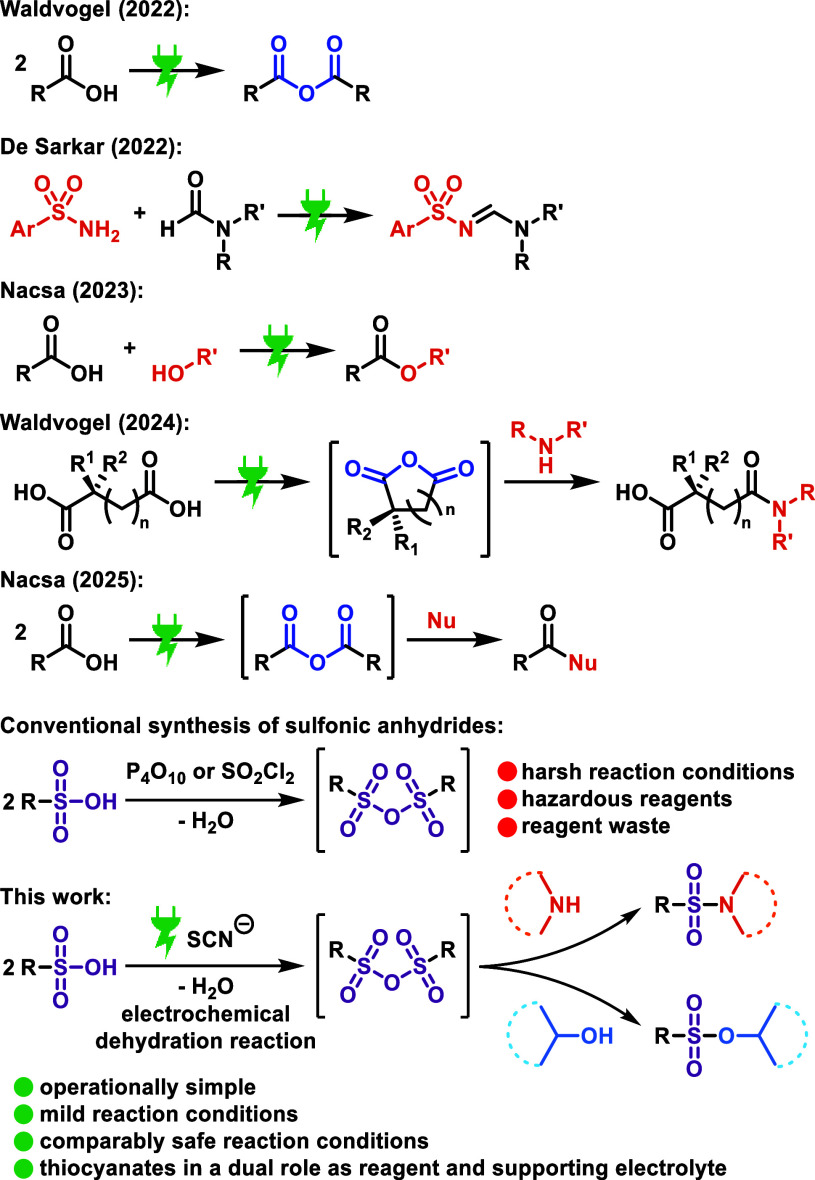
Recent Progress in Electrochemical Dehydration

Specifically, there has been notable progress in the underexplored field of electrochemical dehydration in recent years (Scheme 2). In 2022, our group discovered the electrochemical dehydration of carboxylic acids to their carboxylic anhydrides,^12^ and the group of De Sarkar developed an electrochemical dehydrative coupling of sulfonamides with derivatives of dimethylformamide to *N*-sulfonyl amidines.^13^ In 2023, the group of Nacsa developed an electrochemical design for catalytic dehydration of carboxylic acids for the synthesis of esters,^14^ and in 2024, our group reported on the electrochemical dehydration of dicarboxylic acids to their cyclic anhydrides.^15^ Recently, the Nacsa group developed an electrochemical design for catalytic carboxylic acid substitution via carboxylic anhydrides for the synthesis of amides, esters, and thioesters.^16^ These rapid developments emphasize the increasing interest in electrochemical dehydration reactions. Until now, the main focus in the field of electrochemical dehydration reactions has been the electrosynthesis of carboxylic anhydrides from carboxylic acids. In this work, we report an electrochemical dehydration of sulfonic acids to their sulfonic anhydrides under mild reaction conditions. The sulfonic anhydrides are electrochemically generated, and by the addition of amines or alcohols, they are directly employed in a one-pot reaction for the synthesis of highly value-added sulfonamides and sulfonates, respectively. This approach circumvents the need for conventional dehydrating agents while offering a scalable, milder, and operationally simple route to a class of compounds with broad synthetic applicability.

## Results and Discussion

The reaction optimization started with the knowledge from our previous publications, which used thiocyanate-supporting electrolytes for the electrochemical dehydration of carboxylic acids to their carboxylic anhydrides.^12,15^ The optimized reaction conditions for methanesulfonic anhydride are depicted in Scheme 3. The yields were determined using ^1^H NMR spectroscopy with 1,3,5-trichlorobenzene as internal standard.^17^ With the optimized conditions (Table 1, entry 1), an NMR yield of 74% was achieved. To analyze the reaction, we varied the current density and the amount of applied charge (Table 1, entries 2 and 3). Decreasing the amount of applied charge to only 2 F, a yield of only 62% was obtained (entry 2), and for 50 mA cm^–2^, a lower yield was also observed (entry 3). When using an interelectrode gap of only 3 mm, a yield of 32% was found (entry 4). Next, different electrode materials were evaluated. Notably, the use of graphite as a cathode material resulted in a 49% NMR yield (entry 5), although cathodic corrosion was observed.^18^ When graphite was tested as the anode material, the yield decreased to 37% (entry 6).

**Scheme 3:**
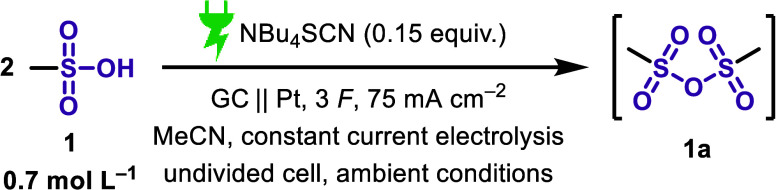
Optimized Reaction Conditions

**Table 1:** Optimization of the Test Reaction

entry	deviation from optimal conditions	NMR yield^a^
1	none	74%
2	2 F	62%
3	50 mA cm^–2^	34%
4	interelectrode gap 3 mm	32%
5	graphite as a cathode material	49%^b^
6	graphite as an anode material	37%
7	KSCN instead of NBu_4_SCN	51%^c^
8	*c*(**1**) = 0.5 mol L^–1^	44%
9	*c*(NBu_4_SCN) = 0.3 mol L^–1^ (0.45 equiv)	42%
10	DMF instead of MeCN	0%
11	50 °C	45%
12	no NBu_4_SCN^d^	0%
13	no electricity	0%

a The yields were determined using ^1^H NMR spectroscopy using 1,3,5-trichlorobenzene as an internal standard. Further details about the experimental setup can be found in the Supporting Information.

b Cathodic corrosion was observed.

c The upper terminal voltage limit of the power supply was reached, but the mentioned amount of applied charge was fully applied.

d For better conductivity, NBu_4_PF_6_ (0.1 mol L^–1^) was used in this case.

The use of KSCN instead of NBu_4_SCN provided moderate yields (entry 7), and the upper terminal voltage limit of the power supply was reached. Additionally, we explored varying concentrations of both the starting material and thiocyanate-supporting electrolyte (entries 8 and 9), as well as different solvents (entry 10), with acetonitrile affording the highest yields. The reaction works best in acetonitrile, which is likely because of its optimal balance of polarity, low nucleophilicity, and high oxidative stability. Increasing the temperature did not lead to an improvement in the yield (entry 11). Without NBu_4_SCN, no traces of product were found. When no electricity was applied, no traces of the product were found (entry 13).

### Scope

With the optimized conditions in hand, we investigated the scope of this reaction by employing various sulfonic acids as starting materials to generate the corresponding sulfonic anhydrides by electrolysis (Scheme 4). Additionally, by adding various amines or alcohols to the reaction solution after electrolysis, we directly employ the in situ-generated sulfonic anhydrides in one-pot sulfonylation reactions to yield the corresponding sulfonamides and sulfonates, respectively. Noteworthy, the sulfonamides and related compounds can also be made electrochemically by multicomponent reactions.^19^ Aliphatic cyclic and acyclic amines were evaluated as nucleophiles in reactions using methanesulfonic acid as starting material, affording the corresponding sulfonamides in up to 64% overall yield (Scheme 4, substrates **2a** to **2g**). Pyrrolidine gave sulfonamide **2a** in 64% isolated yield. Aromatic amines were also examined, yielding compounds **2b** for aniline and **2d** for imidazole in 56 and 63% yields, respectively. Morpholine, an aliphatic heterocyclic amine, provided the sulfonamide product **2c** in 60% isolated yield. Furthermore, indole was tested as a nucleophile, affording the corresponding sulfonamide **2e** in 41% overall isolated yield, likely due to its decreased nucleophilicity.^20^ Products **2f** and **2g** were isolated in 64 and 56% yield, respectively. Next, several alcohols were tested to quench the methanesulfonic anhydride that was generated in situ by electrolysis. Phenol underwent mesylation to furnish **3a** in a 50% yield. Aliphatic primary and secondary alcohols provided mesylates **3b** and **3c** in 66 and 58% isolated yield, respectively. Similarly, **3d** and **3e** were obtained in relatively high yields. The α-hydroxy ester (−) methyl l-lactate was also employed, affording mesylate **3f** in 63% yield. To further explore the scope of this transformation, various sulfonic acids were evaluated as starting materials for the electrochemical generation of the corresponding sulfonic anhydrides, which were subsequently intercepted with pyrrolidine or aniline in a one-pot procedure. The study encompassed both aromatic and aliphatic sulfonic acids to assess their reactivity and influence on the product yield. Aromatic sulfonic acids generally resulted in lower yields, with electron-poor para-substituted derivatives exhibiting particularly poor efficiency (**4a**, **4b**, **4c**, **4d**, **4e**, and **4i**). We observed evidence of ipso-substitution side reactions, which likely contribute to the lower yields obtained with aromatic substrates (see the Supporting Information). **4k** was also isolated in poor yields, probably due to the fact that the benzylic position is easily electrochemically oxidizable.^21^ In contrast, aliphatic sulfonic acids demonstrated significantly higher yields, suggesting a more favorable formation of the anhydride intermediate under the electrochemical conditions. The superior reactivity of aliphatic sulfonic acids may stem from their electronic and steric properties, which facilitate a more efficient anhydride formation and subsequent nucleophilic attack (**4f**, **4g**, **4h**, and **4j**). Moreover, the reaction proceeded smoothly with a variety of aliphatic substrates, reinforcing the robustness and general applicability of this method. These findings highlight the crucial role of electronic effects in determining the efficiency of the sulfonic acid activation and provide valuable insights into substrate selection for optimal reaction performance.

**Scheme 4:**
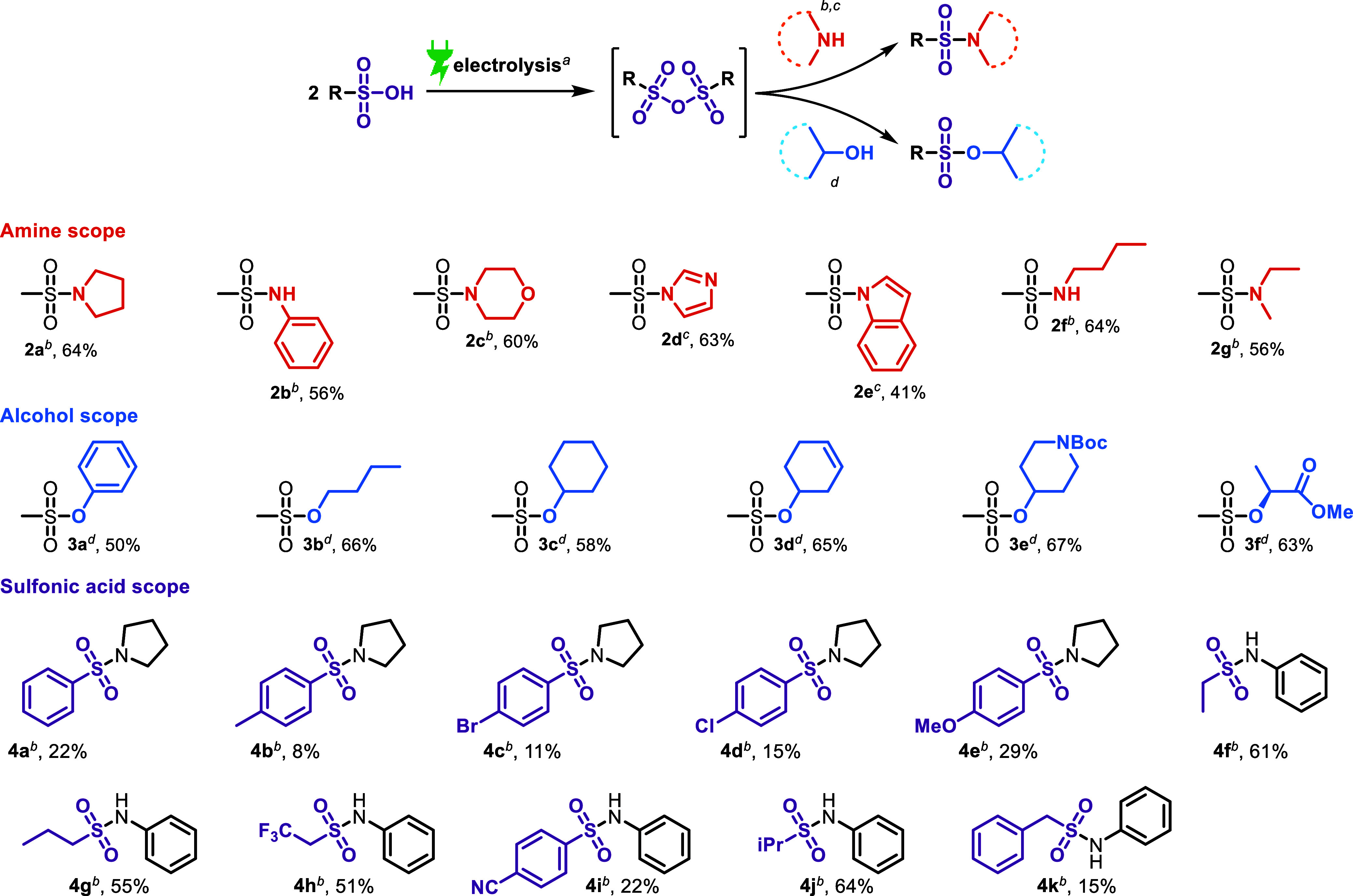
Scope of Amines, Alcohols, and Sulfonic Acids

a Unless stated otherwise, the optimized reaction conditions of Scheme 3 were used.
b Amine (5 equiv)
c Triethylamine (2 equiv) and amine (2 equiv);
d Triethylamine (2 equiv) and alcohol (2 equiv)

### Scale-up

It is important to demonstrate the scalability of an electrochemical protocol because this opens the way for an application on an industrial scale.^22^ To demonstrate the robustness and scalability of our electrochemical dehydration, methanesulfonic acid **1** was electrolyzed to generate methanesulfonic anhydride in situ in a 10-fold scale-up, which is quenched after electrolysis by adding an excess of pyrrolidine as an amine. Upon workup, product **2a** was obtained in 58% yield over two steps (Scheme 5). Further details about this scale-up reaction can be found in the Supporting Information.

**Scheme 5:**
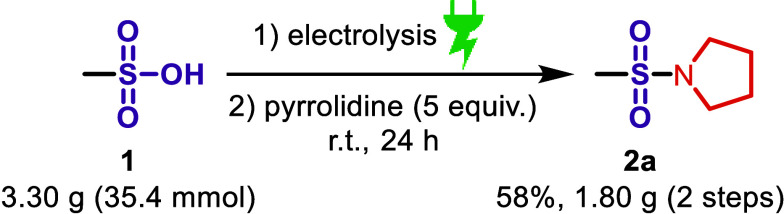
Scale-up Reaction

### Proposed Mechanism

The proposed mechanism for this electrochemical dehydration of sulfonic acids to their sulfonic anhydrides is depicted in Scheme 6. The thiocyanate anion **I** is anodically oxidized to intermediate **II**, which reacts with sulfonate **III** to activated intermediate **IV**. This can react with another equivalent of **III** to form the sulfonic anhydride **VI**, while **V** is eliminated as a good leaving group. The mechanism proposed here represents a simplified picture, as species **V** is known to undergo a variety of hydrolysis, comproportionation, and disproportionation reactions.^23^ It should be noted that during the addition of alcohol or amine after electrolysis, species **IV** is likely to still be present because it has not yet completely reacted with sulfonic acid **III** to the sulfonic anhydride **VI**. If this is the case, then species **IV** itself will react as the electrophile instead of the sulfonic anhydride.

**Scheme 6:**
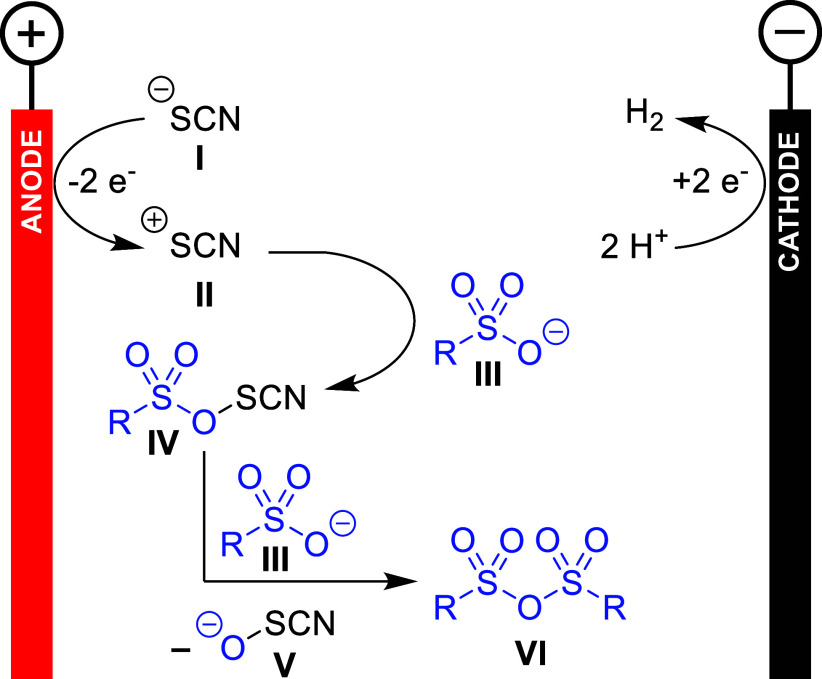
Proposed Reaction Mechanism

In this electrolysis, the thiocyanate-supporting electrolyte acts in a dual role as an enabler of conductivity and as a reagent.^24^ Cyclic voltammetry (CV) studies reveal a nonreversible oxidation of SCN^–^ at +1.45 V, which persisted over multiple scans (Supporting Information, page S11). Upon addition of methanesulfonic acid, this peak decreased and disappeared on the second scan, indicating the rapid trapping of the oxidized species by MsOH. This suggests that oxidized SCN^–^ participates in MsOH anhydride formation. In radical trapping experiments using TEMPO (2,2,6,6-tetramethylpiperidinyloxyl), no radical intermediate could be observed (see the Supporting Information). At the cathode, hydrogen evolution reactions occur as counter reactions.^25^

## Conclusions

In summary, we have transferred our electrochemical dehydration protocol from carboxylic acids to sulfonic acids and established the electrochemical synthesis of sulfonic anhydrides and their subsequent one-pot reaction with amines or alcohols to sulfonamides and sulfonates, respectively. The reaction proceeds under a galvanostatic setup in a simple undivided cell and is carried out at ambient conditions. In total, 24 substrates are demonstrated with up to 67% isolated yield, and the scalability of the protocol is also demonstrated. The thiocyanate-supporting electrolyte serves a dual role as a reagent and enabler of conductivity. This research is part of the very recent advances in the field of electrochemical dehydration,^26,27^ which is an underexplored field of research. Given the enormous importance of dehydration reactions for organic chemistry, electrochemical dehydration reactions promise a milder, safer, and more sustainable approach to such transformations.

## Supplementary Material



## Data Availability

The data underlying this study are available in the published article and its Supporting Information.

## Notes

General aspects, experimental procedures, details of reaction optimization, control experiments, cyclovoltammetry (CV) studies, and NMR spectra of isolated compounds (PDF)
