# Supplementary material for: Electrochemical Dehydration of Sulfonic Acids to Their Anhydrides
Source: J Org Chem. 2025 Aug 22;90(35):12259–64. doi: 10.1021/acs.joc.5c01155 (PMC12418306; doi:10.1021/acs.joc.5c01155)
Supplement: Supplementary file 1 [file jo5c01155_si_001.pdf]

Supporting Information

Electrochemical Dehydration of Sulfonic Acids to their Anhydrides

Enrico Lunghi,[a] Annemijn M. van Koten,[a] Johannes Schneider,[b] and Siegfried R. Waldvogel*[a,c]

E-Mail: siegfried.waldvogel@cec.mpg.de

[a] Max-Planck-Institute for Chemical Energy Conversion (MPI CEC), Stiftstraße 34–36, 45470 Mülheim an der Ruhr (Germany)
[b] Department of Chemistry, Johannes Gutenberg University (JGU), Duesbergweg 10-14, 55128 Mainz
[b] Karlsruhe Institute of Technology (KIT), Institute of Biological and Chemical Systems – Functional Molecular Systems (IBCS FMS), Kaiserstraße 12, 76131 Karlsruhe (Germany)

Table of Contents

GENERAL ASPECTS S3

1.1 General Information S3
1.2 Instruments and Analytical Methods S3
1.3 Electrochemical Setup S3

EXPERIMENTAL PROCEDURES S5

1.4 General protocol for the synthesis of sulfonic anhydrides and their derivatives (GP1) S5
1.5 General protocol for the optimization of reaction conditions (GP2) S5
1.6 General protocol for scale-up S6

OPTIMIZATION S7

CONTROL EXPERIMENTS S10

CYCLOVOLTAMMETRY STUDIES S11

COMPOUND CHARACTERIZATION S14

1.7 1-(Methylsulfonyl)pyrrolidine (2a) S14
1.8 *N*-Phenyl methanesulfonamide (2b) S14
1.9 4-(Methylsulfonyl)morpholine (2c) S14
1.10 1-(Methylsulfonyl)imidazole (2d) S14
1.11 1-(Methylsulfonyl)indole (2e) S14
1.12 *N*-Butyl methanesulfonamide (2f) S15
1.13 *N*-Ethyl-*N*-methyl methanesulfonamide (2g) S15
1.14 *O*-Phenyl methanesulfonate (3a) S15
1.15 *O*-Butyl methanesulfonate (3b) S15
1.16 *O*-Cyclohexyl methanesulfonate (3c) S16
1.17 Cyclohex-3-en-1-yl methanesulfonate (3d) S16
1.18 1,1-Dimethylethyl-4-((methylsulfonyl)oxy)piperidine-1-carboxylate (3e) S16
1.19 Methyl (S)-2-((methylsulfonyl)oxy)propanoate (3f) S16
1.20 1-(Phenylsulfonyl)pyrrolidine (4a) S16
1.21 1-Tosyl pyrrolidine (4b) S17
1.22 1-((4-Bromophenyl)sulfonyl)pyrrolidine (4c) S17
1.23 1-((4-Chlorophenyl)sulfonyl)pyrrolidine (4d) S17
1.24 1-((4-Methoxyphenyl)sulfonyl)pyrrolidine (4e) S17
1.25 *N*-Phenyl ethanesulfonamide (4f) S18
1.26 *N*-Phenyl propanesulfonamide (4g) S18
1.27 *N*-Phenyl-2,2,2-trifluoroethanesulfonamide (4h) S18
1.28 4-Cyano-*N*-phenylbenzenesulfonamide (4i) S18
1.29 *N*-Phenyl 1-methylethanesulfonamide (4j) S18
1.30 *N*-Phenyl phenylmethanesulfonamide (4k) S19
1.31 Limitations of the Scope/Unsuccessful Substrates S19

NMR SPECTRA S21

MASS SPECTRA S45

RADICAL TRAPPING EXPERIMENT S46

AUTHOR CONTRIBUTIONS S47

REFERENCES S48

General aspects

1.1 General Information

If not stated otherwise, all reactions were performed at ambient conditions and chemicals in analytical grade were used as purchased without further purification. Cyclohexane and ethyl acetate used for column chromatography were purchased in HPLC grade. Acetonitrile was commercially obtained in LCMS grade. These solvents were used without further treatment. The IUPAC nomenclature for the substances was checked using OPSIN parser.[1]

1.2 Instruments and Analytical Methods

Chromatography
Thin layer chromatography (TLC) for reaction monitoring was performed using DC Kieselgel 60 F_{254} on aluminium plates (*Merck KGaA,* Darmstadt, Germany). A UV lamp (*λ* = 254 nm, UV-4 S/L, *Herolab GmbH Laborgeräte*, Wiesloch, Germany) and potassium permanganate solution (3 g $KMnO_4$, 20 g K_2CO_3, 5 mL NaOH (5%), 300 mL H_2O) were used for staining of TLC plates. Preparative column chromatography was performed manually on 100-200 mesh silica gel.

Gas Chromatography coupled with Mass Spectrometry (GC/MS)
Analysis of crude reaction mixtures and purified products were performed using a GCMS QP2010SE (*Shimadzu*, Kyoto, Japan) equipped with an electron ionization (EI) source and a quadrupole mass analyzer. A quartz capillary column HI-5MS (*Avantor VWR*, Radnor, USA) with the following specification was used: length of 30 m, inner diameter of 0.25 mm and a stationary phase [(5%-phenyl)-dimethylsiloxane] of 0.25 µm thickness. Helium was used as carrier gas with a constant velocity of 30 cm/s. The GC temperature ramp started at 50 °C (holding for 1 min) and heated to 300 °C (holding for 4.71 min) with a temperature ramp of 17.5 °C/min (total program time: 20.0 min). Measurements were performed at an injector temperature of 270 °C and a temperature of the EI source of 250 °C.

Nuclear Magnetic Resonance (NMR) Spectroscopy
^{1}H NMR and ^{13}C{^{1}H} NMR spectra were recorded at 25°C on a Bruker AVANCE III HD 400 MHz NMR spectrometer with a Bruker Prodigy probe (*Bruker BioSpin GmbH*, Rheinstetten, Germany) using $CDCl_3$ or DMSO as deuterated solvent. All chemical shifts are reported in δ-scale as parts per million [ppm] (multiplicity, coupling constant *J*, number of protons), relative to the solvent residual peaks as the internal standard.[2] Coupling constants *J* are given in Hertz [Hz]. The following abbreviations were used to describe the signals: s (singlet), d (doublet), t (triplet), q (quartet), pent (pentet), sext (sextet), hept (heptet), m (multiplet), br (broad signal). The spectra obtained were evaluated with MestReNova 14 (*Mestrelab Research S.L.*, Spain).

High Resolution Mass Spectrometry
Mass spectra *via* electrospray-ionization (ESI+) were recorded using a Q Exactive™ mass spectrometer (*Thermo Fischer Scientific*™, Waltham, USA). Mass spectra *via* electron-ionization (EI) were recorded using a Q Exactive™ GC Orbitrap™ GC-MS/MS (*Thermo Fischer Scientific*™, Waltham, USA) equipped with an XTI-5 column (*Restek GmbH*, Bad Homburg v. d. Höhe, Deutschland).

Cyclic Voltammetry (CV) Measurements
Cyclic voltammetry was performed using a Metrohm 663 VA Stand equipped with an Autolab type III potentiostat (*Metrohm AG*, Herisau, Switzerland). WE: glassy carbon electrode (*d* = 2 mm); CE: glassy carbon rod; RE: Ag/$AgNO_3$; Scan rate *v* = 100 mV/s. Electrolyte: NBu_4BF_4 (0.1 mol/L) in acetonitrile; *c*(substrate) = 10 mmol/L; *V* = 5 mL. All solutions have been deoxygenated by bubbling with argon for 10 min prior to measurement. Electrodes have been thoroughly rinsed before and after each measurement with acetone and acetonitrile. The glassy carbon working electrode was polished with alumina paste (1 µm followed by 0.1 µm) and sonicated for 5 min each in between measurements. All data is displayed against the half-wave potential of ferrocene/ferrocenium redox couple (FcH/FcH^+; –0.10 V vs. Ag/$AgNO_3$) as internal reference. Oxidation potentials are marked and displayed as the half-wave potential of the respective peak.[3]

1.3 Electrochemical Setup

Galvanostat (DC power supply)
Electrochemical reactions were carried out using a multichannel galvanostat HMP4040 programmable power supply (4 channels per device; max. electric current per channel: 10 A; max. power per channel: 160 W; total output power per device: 384 W; upper terminal voltage limit per channel: 32 V; Rohde & Schwarz GmbH & Co. KG, Munich, Germany).

Screening and small-scale batch-type reactions
Screening reactions were carried out in undivided/quasi-dived Teflon™ cells with a volume of 5 mL equipped with two electrodes and a round, cross-shaped stirring bar. The described system is commercially available as IKA Screening System Package (*IKA™ Werke GmbH & Co. KG*, Staufen, Germany). Detailed description of these cells are reported in the literature.[4]

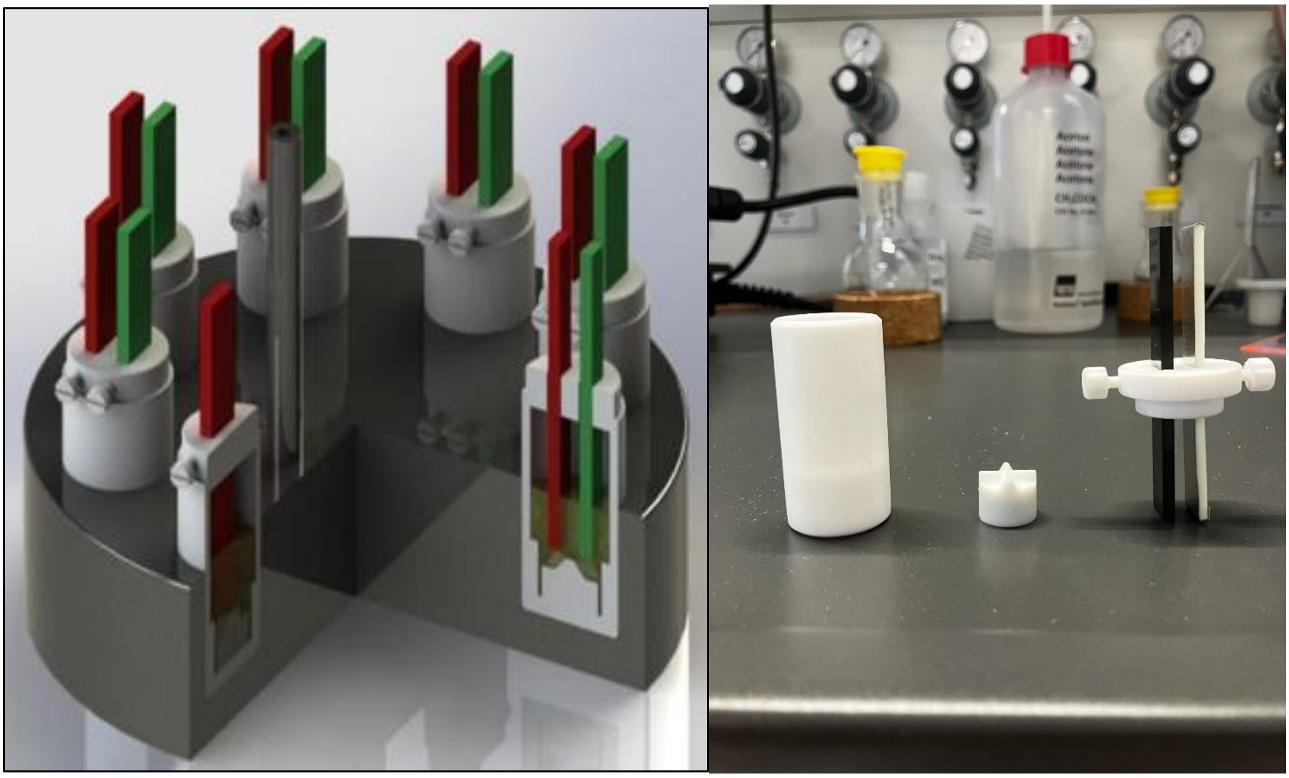

Figure S1: Undivided screening setup as available from IKA equipped with glassy carbon as anode and platinum as cathode (left) and undivided cell equipped with platinum foil and glassy carbon (right).[5]

The screening was carried out using various electrode materials (electrode size: 7 cm × 1 cm × 0.3 cm). Graphite electrodes were sanded using sandpaper of grit size 600, followed by grit size 1000, and cleaned with acetonitrile prior to use. BDD electrodes were electrochemically treated as anode (j = 50 mA/cm^2) in 20% (v/v) sulfuric acid for 10 min before use. The anodes were immersed 1.8 cm into the electrolyte. This resulted in a geometric active electrode area of 1.8 cm^2.

Table S1: Electrode materials, specification, and supplier.

Entry	Electrode Material	Specification	Supplier
1	Boron-doped diamond (BDD)	15 µm BDD on silicon carrier material	*CONDIAS GmbH*, Itzehoe, Germany
2	Glassy Carbon	Sigradur G	*HTW*, Thierhaupten, Germany
3	Graphite	highly isostatic, V2100	*SGL Carbon*, Bonn, Germany
4	Graphite foil (Sigraflex™)	F02012Z	*SGL Carbon*, Meitingen, Germany
5	Platinum	99.9% Pt	ÖGUSSA, Vienna, Austria.

Scale-up

The scale-up experiment was performed in a double-walled undivided glass cell with no thermostat attached, with a volume of 100 mL equipped with a PTFE stopper and sleeve, electrodes, electrode holders and a cross-shaped stirring bar. The cell is commercially available *via HWS Labortechnik* (Mainz, Germany) as Sigma-Aldrich within the SynLectro™ series. The electrolysis was carried out using a TDK-Lambda Z+ series (*TDK-Lambda UK Limited*, Devon, United Kingdom) as power supply. The glassy carbon anode was held in place using a PTFE support frame. Its dimensions were 6 cm × 2 cm × 0.02 cm and was half submerged into the solution. This resulted in an active electrode area of 6 cm^2. As cathode, a platinum foil (3 x 2 cm) was used and placed in front of the anode by a PTFE support frame. The interelectrode gap is 0.5 cm. The stoppers and electrode holders are available from Sigma-Aldrich within the SynLectro™ series.

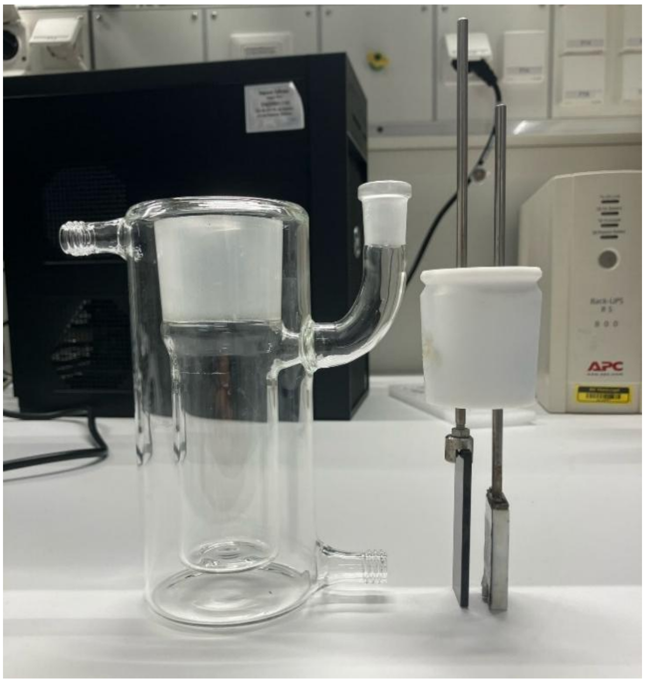

Figure S2: 100 mL undivided cell with glassy carbon anode and platinum cathode and electrode holders used for the scale-up.

Experimental Procedures

1.4 General protocol for the synthesis of sulfonic anhydrides and their derivatives (GP1)

Caution! *Methanesulfonic acid (CAS 75-75-2) and methanesulfonic anhydride (CAS 7143-01-3) are highly corrosive and cause severe skin burns and eye damage! They must be handled with extreme care!*

The reactions are carried out using the undivided Teflon™ cells with a glassy carbon anode and a platinum foil as a cathode as described in the section before.

Electrolyte: An undivided Teflon™ cell is filled with a sulfonic acid (3.54 mmol, 1 equiv.), tetrabutylammonium thiocyanate (0.5 mmol, 0.15 equiv.), and acetonitrile (5 mL).

The lid including a prefixed glassy carbon anode and a platinum cathode is attached to the cell, the electric current is set accordingly so that a current density of 75 mA/cm^2 is reached (172 mA with the setup described herein) and the amount of applied charge is set to 3 *F* (1143 C for 3.54 mmol of starting material). The electrolysis is carried out at room temperature under constant stirring (900 rpm) for ca. 1 h 50 min. After completion of the electrolysis, 5 equiv. of an amine (**2a**, **2b**, **2c**, **2f, 2g**, **4a-k**) or 2 equiv. of triethylamine and 2 equiv. of amine (**2d**, **2e**) or 2 equiv. of triethylamine and 2 equiv. of an alcohol (**3a**-**f**) are added to the reaction mixture in the same reaction vessel and the solution is stirred overnight. The solvent is then removed under vacuum and the residue is dissolved in ethyl acetate, washed two times with aqueous HCl (1 mol/L, 2x10 mL), two times with saturated aqueous $NaHCO_3$ solution (2x10 mL) and one time with brine (1x10 mL). The organic layer is then dried over $MgSO_4$, filtered and concentrated under vacuum. The crude was then purified *via* column chromatography using cyclohexane and ethyl acetate.

1.5 General protocol for the optimization of reaction conditions (GP2)

The reactions are carried out using the undivided Teflon™ cells with a PTFE lid including the anode and a cathode as described in the section before.

Electrolyte: An undivided Teflon™ cell is filled with methanesulfonic acid (3.54 mmol, 1 equiv.), tetrabutylammonium thiocyanate (0.5 mmol, 0.15 equiv.), and acetonitrile (5 mL).

The lid including the electrodes is attached to the cell, the electric current and amount of applied charge are set to the desired values, and the electrolysis was carried out at room temperature under constant stirring (900 rpm). After completion, 1,3,5-trichlorobenzene (60 mg) is added to the reaction mixture as an internal standard.[2] The solution is stirred for 5 min at room temperature and an aliquot (ca. 0.5 mL) is taken for qNMR measurement (^{1}H NMR, 400 MHz). The yield is calculated using the six protons of the product **2a** (Figure S3).

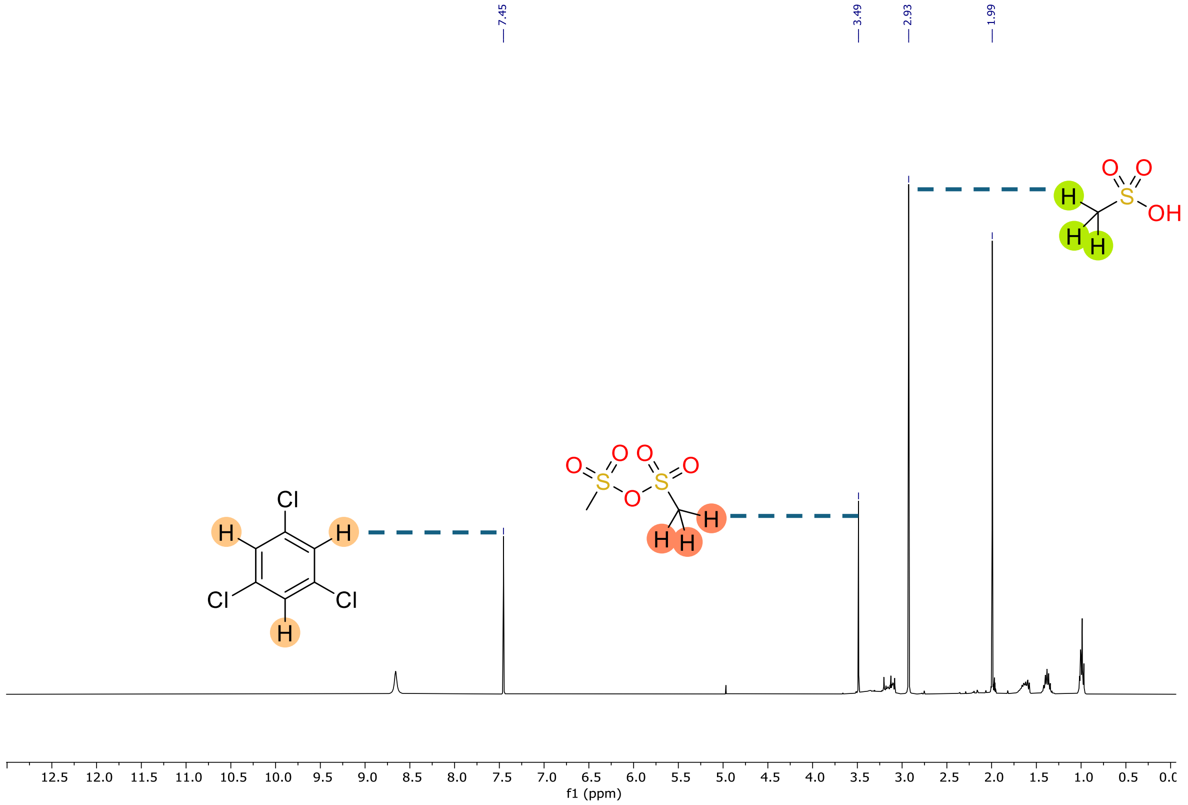

Figure S3: Typical ^{1}H NMR spectrum in CD_3CN of a reaction mixture used for quantification of product **2a**.[2]

1.6 General protocol for scale-up

The reaction is carried out using the undivided glass cell with a glassy carbon anode and a platinum foil as a cathode as described in the section above.

Electrolyte: The glass cell is filled with methanesulfonic acid (**1**, 2.25 mL, 35.4 mmol, 1 equiv.), NBu_4SCN (1.8 g, 5 mmol, 0.15 equiv.), and acetonitrile (50 mL).

The stopper including the electrodes and electrode holders is attached to the cell and connected to the power supply. The electric current is set accordingly so that a current density of 75 mA/cm^2 is reached (345 mA with the setup described herein) and the amount of applied charge is set to 3 *F* (corresponds to 11430 C when using 35.4 mmol of starting material). The electrolysis is carried out at room temperature under constant stirring (500 rpm) for ca. 9 h 35 min. After completion of the electrolysis, NMR yield was determined as described in **GP1**: 1,3,5-trichlorobenzene (600 mg) are dissolved in the reaction mixture. The solution was stirred for 5 min at room temperature and an aliquot (ca. 0.5 mL) was taken for qNMR measurement (^{1}H NMR, 400 MHz).

Spectroscopic data of the compound matched the one obtained from the small-scale reaction described in section “NMR Spectra”.

Optimization

Optimization of the reaction conditions was carried out by using a linear screening approach.[4] Methanesulfonic acid (MsOH) was used as a test substrate according to **GP2**. Yields were determined by ^{1}H NMR spectroscopy in CD_3CN *via* the addition of 1,3,5-trichlorobenzene (60 mg) as an internal standard after the end of electrolysis.[2]

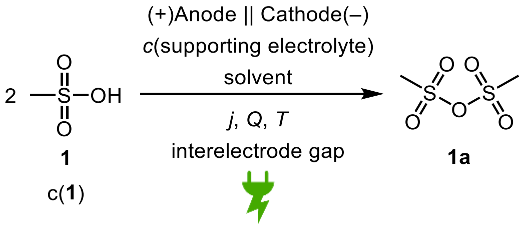

Initial experiments were carried out in an undivided Teflon™ cell with a planar glassy carbon anode and cathode (Table S2), with KSCN as supporting electrolyte.

Table S2: First experiments.

Entry	Anode	Cathode	Yield (^{1}H NMR)
1	Graphite plate	Graphite plate	0%
2	Graphite plate	Stainless-steel plate	0%
3	Glassy carbon	Glassy carbon	11%

Conditions (GP2): KSCN (0.5 mmol, 0.15 equiv., 0.1 M), MeCN, j = 50 mA cm^{-2}, 2 F, r.t., 5 mm interelectrode gap, 900 rpm.

Next, the current density was screened (Table S3). 75 mA cm^{-2} gave the best results, and screening was continued with this parameter. This is also beneficial because a higher current density decreases the electrolysis time.

Table S3: Screening of current densities.

j [mA cm^{-2}]	1	1a
10	93%	5%
25	90%	8%
50	62%	11%
75	62%	34%
100	70%	26%

Conditions (GP2): KSCN (0.5 mmol, 0.15 equiv., 0.1 mol/L), (+)GC||GC(-), MeCN, 2 F, r.t., 5 mm interelectrode gap, 900 rpm.

Screening of various interelectrode gaps (Table S4) showed no improvement to the previously used conditions, so the optimization was continued with 5 mm.

Table S4: Screening of interelectrode gap.

gap [mm]	1	1a
1	70%	28%
2	69%	28%
3	66%	32%
4	60%	34%
5	61%	34%

Conditions (GP2): KSCN (0.5 mmol, 0.15 equiv., 0.1 mol/L), (+)GC||GC(–), j = 75 mA cm^{-2}, MeCN, 2 F, r.t, 900 rpm.

Table S5: Screening of cathode materials.

Cathode	1	1a
GC	62%	34%
graphite	47%	49%
BDD	77%	22%
RVC	70%	27%
Pt	45%	51%

Conditions (GP2): KSCN (0.5 mmol, 0.15 equiv., 0.1 mol/L), j = 75 mA cm^{-2}, GC anode, MeCN, 2 F, r.t., interelectrode gap 5 mm, 900 rpm. GC: glassy carbon, RVC: reticulated vitreous carbon foam, BDD: boron-doped diamond.

Table S6: Screening of anode materials.

Anode	1	1a
GC	45%	51%
Pt	56%	42%
Gr	60%	37%
Zn	-	-
Ni (foam)	-	-
BDD	56%	41%

Conditions (GP2): KSCN (0.5 mmol, 0.15 equiv., 0.1 mol/L), j = 75 mA cm^{-2}, MeCN, Pt cathode, 2 F, r.t., interelectrode gap 5 mm, 900 rpm.

Table S7: Screening of supporting electrolytes.

Supporting Electrolyte	1	1a
KSCN	45%	51%
EMIMSCN	90%	10%
NBu_4SCN	36%	62%

Conditions (GP2): supporting electrolyte (0.5 mmol, 0.15 equiv., 0.1 mol/L), j = 75 mA cm^{-2} , (+)GC||Pt(–), MeCN, 2 F, r.t., interelectrode gap 5 mm, 900 rpm. EMIMSCN: 1-Ethyl-3-methylimidazolium thiocyanate.

Table S8: Screening of starting material concentrations.

$c(CH_3SO_3H)$ [mol L^{-1}]	1	1a
0.1	80%	17%
0.3	72%	26%
0.5	50%	44%
0.7	36%	62%
1.0	51%	44%

Conditions (GP2): NBu_4SCN (0.5 mmol, 0.15 equiv., 0.1 mol/L), (+)GC||Pt(–), j = 75 mA cm^{-2} MeCN, 2 *F*, r.t., interelectrode gap 5 mm, 900 rpm.

Table S9: Screening of solvents.

Solvent	1	1a
DMSO	62%	0%
DMF	74%	0%
DMC	91%	7%
DMC/PC	84%	10%
MeCN	36%	62%

Conditions (GP2): NBu_4SCN (0.5 mmol, 0.15 equiv., 0.1 mol/L), j = 75 mA cm^{-2}, (+)GC||Pt(–), 2 *F*, r.t., interelectrode gap 5 mm, 900 rpm.
DMSO: dimethyl sulfoxide, DMF: dimethyl formamide, DMC: dimethyl carbonate, PC: propylene carbonate, MeCN: acetonitrile.

Table S10: Screening of the amount of applied charge.

Q [*F*]	1	1a
2.0	36%	62%
2.5	27%	70%
3.0	22%	74%
3.5	45%	51%

Conditions (GP2): NBu_4SCN (0.5 mmol, 0.15 equiv., 0.1 mol/L), j = 75 mA cm^{-2}, MeCN, (+)GC||Pt(–), r.t., interelectrode gap 5 mm, 900 rpm.

Table S11: Screening of concentrations of the supporting electrolyte NBu_4SCN.

$c(NBu_4SCN)$ [mol L^{-1}]	1	1a
0.07	42%	55%
0.1	22%	74%
0.3	52%	42%
0.5	58%	39%
0.7	75%	28%

Conditions (GP2): NBu_4SCN, *j* = 75 mA cm^{-2}, MeCN, 3 *F*, (+)GC||Pt(–), r.t., interelectrode gap 5 mm, 900 rpm.

Table S12: Screening of temperatures.

T [°C]	1	1a
0	66%	29%
r.t.	22%	74%
50	50%	45%

Conditions (GP2): NBu_4SCN (0.5 mmol, 0.15 equiv., 0.1 mol/L), *j* = 75 mA cm^{-2}, MeCN, 3 *F*, (+)GC||Pt(–), interelectrode gap 5 mm, 900 rpm.

Table S13: Final optimized reaction conditions.

c(SM)	Supporting Electrolyte (SE)	*c*(SE)	*j* [mA cm^{-2}]	Cathode Material	Anode Material	*T*	*Q* [*F*]	Yield 1a (^{1}H NMR)
0.7	NBu_4SCN	0.1	75	Pt	GC	r.t.	3	74%

Control Experiments

Several control experiments (Table S14) were performed using the optimized conditions (Table S13).

Table S14: Control experiments.

Entry	Deviation from the standard conditions[a]	Yield (^{1}H NMR)
1	No electricity; stirred for 1.5 h at room temperature	0%
2	No SCN supporting electrolyte (0.1 mol/L NBu_4PF_6 added for conductivity)	0%

[a]: Conditions (GP2): NBu_4SCN (0.5 mmol, 0.15 equiv., 0.1 mol/L), *j* = 75 mA cm^{-2}, MeCN, 3 *F*, (+)GC||Pt(–), r.t., interelectrode gap 5 mm, 900 rpm.

Cyclovoltammetry Studies

To investigate the interaction between the thiocyanate anion and methanesulfonic acid, we carried out cyclic voltammetry (CV) studies. When tetrabutylammonium thiocyanate was the only component in a solution in acetonitrile, a non-reversible oxidation peak was observed at +1.45 V (Figure S3, blue curve), indicating the oxidation of the SCN^- anion. A subsequent scan of the same solution decreased the oxidation peak, confirming that the oxidized species was neither fully consumed nor sequestered by another component. In contrast, upon the addition of methanesulfonic acid (MsOH), the first scan exhibited a broader oxidation peak at +1.45 V with a decreased current response, suggesting rapid trapping of the oxidation product by MsOH (Figure S3, orange curve). In the second scan, the oxidation peak was no longer detected, providing further evidence that the oxidized thiocyanate species had been effectively sequestered by MsOH.

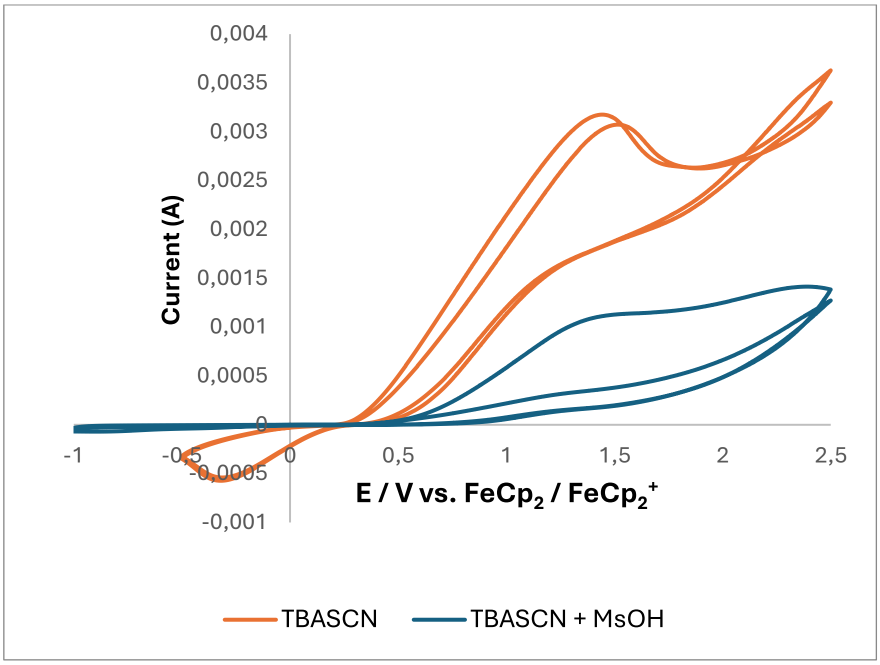

Figure S4: Cyclic voltammogram of 0.1 mol/L NBu_4SCN in MeCN (orange curve), addition of methanesulfonic acid 0.06 mol/L (blue curve), Ag^+/Ag reference electrode (RE), glassy carbon as working electrode (WE) and counter electrode (CE). All CV measurements were carried out under argon atmosphere. Each CV measurement was referenced against ferrocene. Oxidation potentials are marked and displayed as the half-wave potential of the respective peak (IUPAC convention). Scan has been taken in this direction: 0 V → +2.5 V → –0.5 V → 0 V.

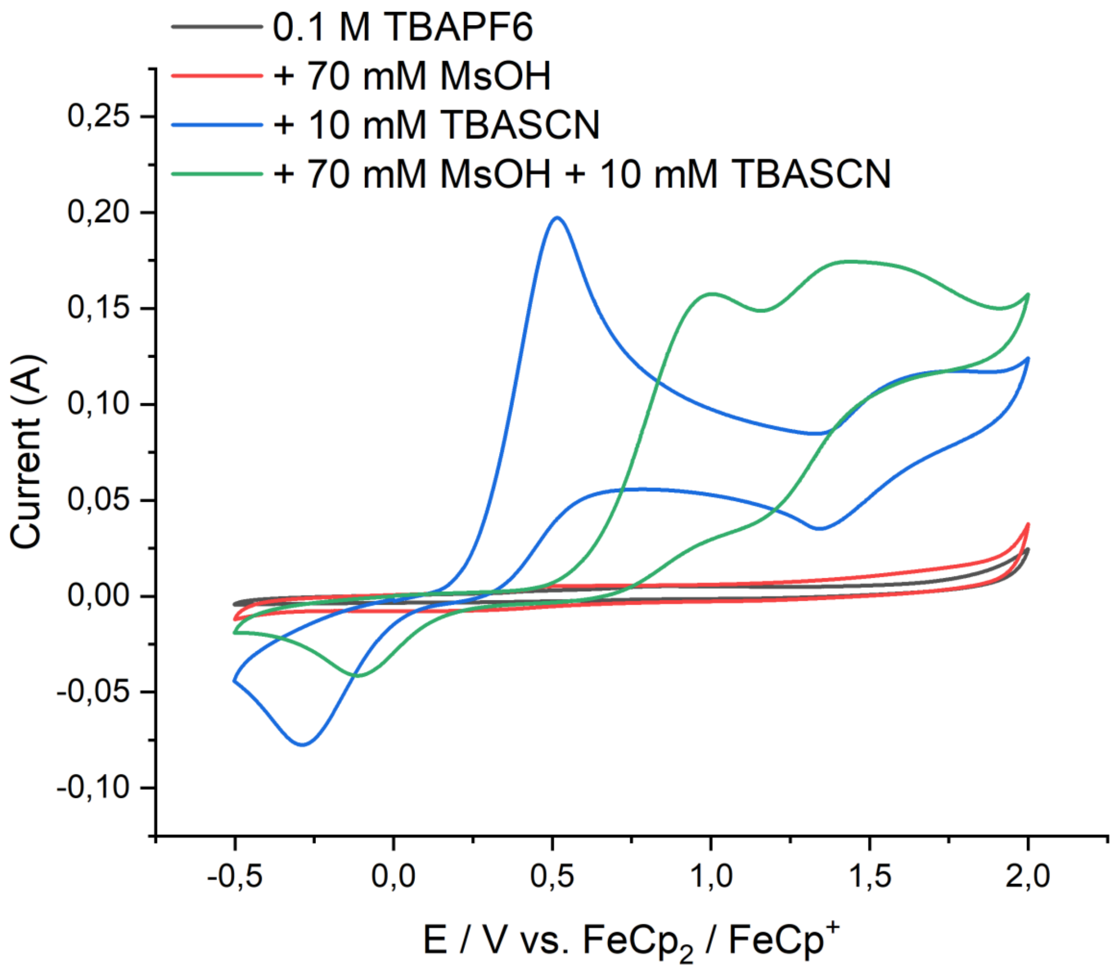

Figure S5: Cyclic voltammogram of 0.1 mol/L NBu_4PF_6 in MeCN (gray curve), addition of methanesulfonic acid 0.07 mol/L (red curve). CV of NBu_4SCN 10 mM in NBu_4PF_6 (blue curve), addition of methanesulfonic acid 70 mM (green curve). Ag^+/Ag reference electrode (RE), glassy carbon as working electrode (WE) and counter electrode (CE). All CV measurements were carried out under argon atmosphere. Each CV measurement was referenced against ferrocene. Oxidation potentials are marked and displayed as the half-wave potential of the respective peak (IUPAC convention). Scan has been taken in this direction: 0 V → +2.0 V → –0.5 V → 0 V.

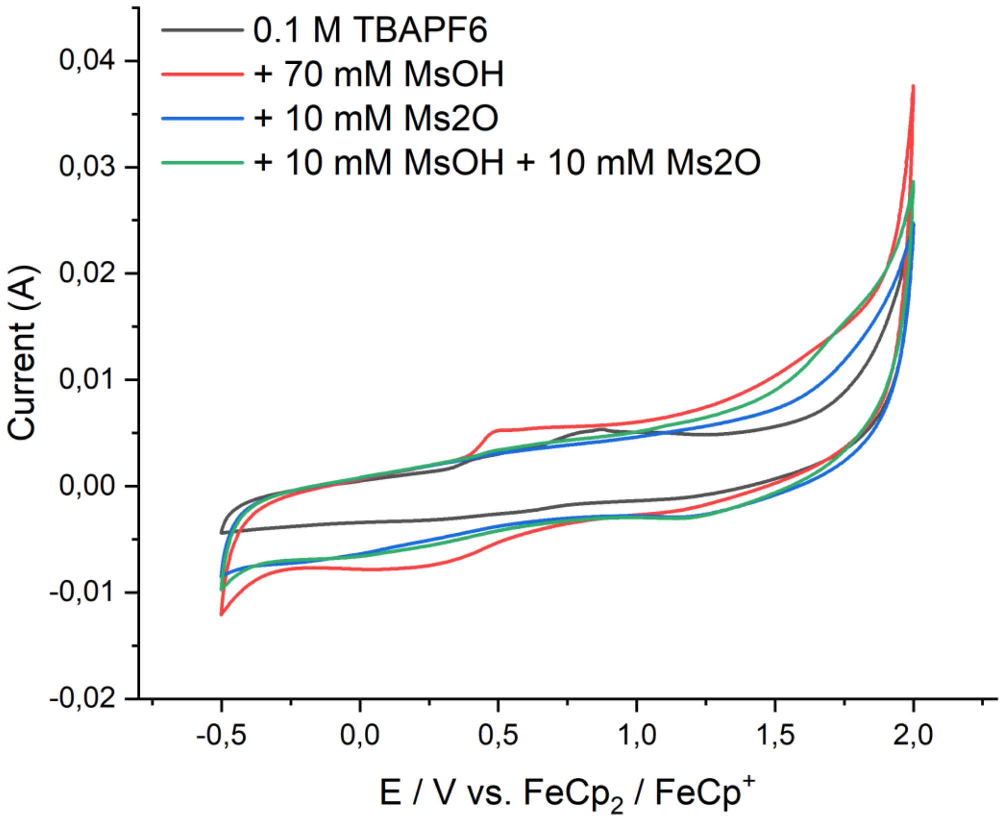

Figure S6: Cyclic voltammogram of 0.1 mol/L NBu_4PF_6 in MeCN (gray curve), addition of methanesulfonic acid 0.07 mol/L (red curve). CV of methanesulfonic anhydride 10 mM in NBu_4PF_6 (blue curve), addition of methanesulfonic acid 70 mM (green curve). Ag^+/Ag reference electrode (RE), glassy carbon as working electrode (WE) and counter electrode (CE). All CV measurements were carried out under argon atmosphere. Each CV measurement was referenced against ferrocene. Oxidation potentials are marked and displayed as the half-wave potential of the respective peak (IUPAC convention). Scan has been taken in this direction: 0 V → +2.0 V → –0.5 V → 0 V.

Compound Characterization

1.7 1-(Methylsulfonyl)pyrrolidine (2a)

According to GP1, methanesulfonic acid (MsOH) (340 mg, 3.54 mmol, 1 equiv.), tetrabutylammonium thiocyanate (NBu_4SCN) (180 mg, 0.5 mmol, 0.15 equiv.), and pyrrolidine (17.7 mmol, 1.5 mL, 5 equiv.) were employed. The electrolysis was carried out using 3 *F*. After purification by column chromatography (cyclohexane/ethyl acetate = 9/1 → 6/4), the desired product **2a** (181 mg, 1.2 mmol, 64%) was obtained as a yellow oil.

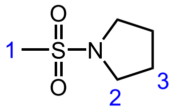

^{1}H NMR (400 MHz, $CDCl_3$) δ = 3.31 (t, *J* = 6.8 Hz, 4H, 2-*H*), 2.80 (s, 3H, 1-*H*), 1.99 – 1.86 (m, 4H, 3-*H*) ppm.
^{13}C{^{1}H} NMR (101 MHz, $CDCl_3$) δ = 48.0, 34.6, 25.8 ppm.
This NMR data agrees with the data previously reported in literature.[6]

1.8 *N*-Phenyl methanesulfonamide (2b)

According to GP1, methanesulfonic acid (MsOH) 340 mg, 3.54 mmol, 1 equiv.), tetrabutylammonium thiocyanate (NBu_4SCN) (180 mg, 0.5 mmol, 0.15 equiv.), and aniline (17.7 mmol, 1.6 mL, 5 equiv.) were employed. The electrolysis was carried out using 3 *F*. After purification by column chromatography (cyclohexane/ethyl acetate = 9/1 → 6/4), the desired product **2b** (170 mg, 1 mmol, 56%) was obtained as a yellow oil.

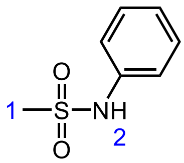

^{1}H NMR (400 MHz, DMSO) δ = 9.73 (s, 1H, 2-*H*), 7.40 – 7.26 (m, 2H, Ar-*H*), 7.26 – 7.16 (m, 2H, Ar-*H*), 7.15 – 7.04 (m, 1H, Ar-*H*), 2.97 (s, 3H, 1-*H*) ppm.
^{13}C{^{1}H} NMR (101 MHz, DMSO) δ = 138.4, 129.4, 123.9, 119.8, 39.2 ppm.
This NMR data agrees with the data previously reported in literature.[7]

1.9 4-(Methylsulfonyl)morpholine (2c)

According to GP1, methanesulfonic acid (MsOH) (340 mg, 3.54 mmol, 1 equiv.), tetrabutylammonium thiocyanate (NBu_4SCN) (180 mg, 0.5 mmol, 0.15 equiv.), and morpholine (17.7 mmol, 1.5 mL, 5 equiv.) were employed. The electrolysis was carried out using 3 *F*. After purification by column chromatography (cyclohexane/ethyl acetate = 9/1 → 6/4), the desired product **2c** (174 mg, 1.05 mmol, 60%) was obtained as a yellow oil.

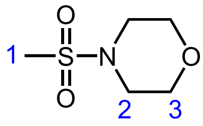

^{1}H NMR (400 MHz, $CDCl_3$) δ = 3.83 – 3.75 (m, 4H, 2-*H*), 3.25 – 3.17 (m, 4H, 3-*H*), 2.79 (s, 3H, 1-*H*) ppm.
^{13}C{^{1}H} NMR (101 MHz, $CDCl_3$) δ = 66.3, 45.9, 34.1, 29.7 ppm.

1.10 1-(Methylsulfonyl)imidazole (2d)

According to GP1, methanesulfonic acid (MsOH) (340 mg, 3.54 mmol, 1 equiv.), tetrabutylammonium thiocyanate (NBu_4SCN) (180 mg, 0.5 mmol, 0.15 equiv.), and imidazole (17.7 mmol, 1.2 g, 5 equiv.) were employed. The electrolysis was carried out using 3 *F*. After purification by column chromatography (cyclohexane/ethyl acetate = 1/1 → 3/7), the desired product **2d** (160 mg, 1.1 mmol, 63%) was obtained as a white solid.

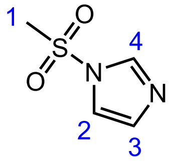

^{1}H NMR (400 MHz, $CDCl_3$) δ = 7.96 (m, 1H, 4-*H*), 7.32 (m, 1H, 2-*H*), 7.16 (m, 1H, 3-*H*), 3.27 (s, 3H, 1-*H*) ppm.
^{13}C{^{1}H} NMR (101 MHz, $CDCl_3$) δ = 136.6, 131.7, 117.4, 43.8 ppm.
This NMR data agrees with the data previously reported in literature.[9]

1.11 1-(Methylsulfonyl)indole (2e)

According to GP1, methanesulfonic acid (MsOH) (340 mg, 3.54 mmol, 1 equiv.), tetrabutylammonium thiocyanate (NBu_4SCN) (180 mg, 0.5 mmol, 0.15 equiv.), triethylamine (1.5 mL, 10.6 mmol, 3 equiv.) and indole (7.08 mmol, 828 mg, 2 equiv.) were employed. The electrolysis was carried out using 3 *F*. After purification by column chromatography (cyclohexane/ethyl acetate = 1/1 → 4/6), the desired product **2e** (140 mg, 0.71 mmol, 41%) was obtained as a viscous colorless oil.

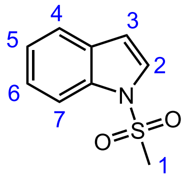

^{1}H NMR (400 MHz, $CDCl_3$) δ = 7.85 (dq, *J* = 8.3, 0.9 Hz, 1H, 7-*H*), 7.61 – 7.52 (m, 1H, 4-*H*), 7.37 (d, *J* = 3.7 Hz, 1H, 6-*H*), 7.30 (ddd, *J* = 8.4, 7.1, 1.4 Hz, 1H, 2-*H*), 7.26 – 7.20 (m, 1H, 5-*H*), 6.64 (dd, *J* = 3.7, 0.9 Hz, 1H, 3-*H*), 3.02 (s, 3H, 1-*H*) ppm.
^{13}C{^{1}H} NMR (101 MHz, $CDCl_3$) δ = 134.7, 130.5, 125.9, 124.7, 123.4, 121.5, 112.8, 108.7, 40.5 ppm.
This NMR data agrees with the data previously reported in literature.[10]

1.12 *N*-Butyl methanesulfonamide (2f)

According to GP1, methanesulfonic acid (MsOH) (340 mg, 3.54 mmol, 1 equiv.), tetrabutylammonium thiocyanate (NBu_4SCN) (180 mg, 0.5 mmol, 0.15 equiv.), and 1-butylamine (17.7 mmol, 1.7 mL, 5 equiv.) were employed. The electrolysis was carried out using 3 *F*. After purification by column chromatography (cyclohexane/ethyl acetate = 9/1 → 6/4), the desired product **2f** (171 mg, 1.13 mmol, 64%) was obtained as a colorless oil.

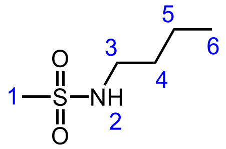

^{1}H NMR (400 MHz, $CDCl_3$) δ = 4.74 (s, 1H, 2-*H*), 3.09 (t, *J* = 7.1 Hz, 2H, 3-*H*), 2.93 (s, 3H, 1-*H*), 1.59 – 1.47 (m, 2H, 4-*H*), 1.43 – 1.29 (m, 2H, 5-*H*), 0.91 (t, *J* = 7.3 Hz, 3H, 6-*H*) ppm.
^{13}C{^{1}H} NMR (101 MHz, $CDCl_3$) δ = 43.1, 40.1, 32.1, 19.8, 13.7 ppm.
This NMR data agrees with the data previously reported in literature.[11]

1.13 *N*-Ethyl-*N*-methyl methanesulfonamide (2g)

According to GP1, methanesulfonic acid (MsOH) (340 mg, 3.54 mmol, 1 equiv.), tetrabutylammonium thiocyanate (NBu4SCN) (180 mg, 0.5 mmol, 0.15 equiv.), and methyl ethyl amine (17.7 mmol, 1.5 mL, 5 equiv.) were employed. The electrolysis was carried out using 3 *F*. After purification by column chromatography (cyclohexane/ethyl acetate = 9/1 → 6/4), the desired product **2g** (135 mg, 0.99 mmol, 56%) was obtained as colorless oil.

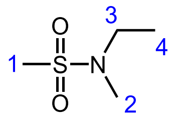

^{1}H NMR (400 MHz, $CDCl_3$) δ = 3.23 (q, *J* = 7.1 Hz, 2H), 2.85 (s, 3H), 2.79 (s, 3H), 1.22 (t, *J* = 7.2 Hz, 3H) ppm.
^{13}C{^{1}H} NMR (101 MHz, $CDCl_3$) δ = 44.8, 35.8, 33.9, 13.3 ppm.
HRMS (EI, m/z): [M]$^+$ = calcd. For $C_4H_{11}NO_2S^+$: 137.050501, found: 137.050500.

1.14 *O*-Phenyl methanesulfonate (3a)

According to GP1, methanesulfonic acid (MsOH) (340 mg, 3.54 mmol, 1 equiv.), tetrabutylammonium thiocyanate (NBu_4SCN) (180 mg, 0.5 mmol, 0.15 equiv.), triethylamine (1.5 mL, 10.6 mmol, 3 equiv.) and phenol (7.08 mmol, 665 mg, 5 equiv.) were employed. The electrolysis was carried out using 3 *F*. After purification by column chromatography (cyclohexane/ethyl acetate = 9/1 → 7/3), the desired product **3a** (115 mg, 0.66 mmol, 50%) was obtained as a white crystalline solid.

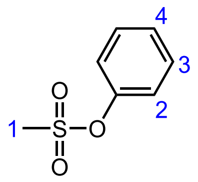

^{1}H NMR (400 MHz, $CDCl_3$) δ = 7.39 – 7.30 (m, 2H, 3-*H*), 7.28 – 7.17 (m, 3H, 2-4*H*), 3.05 (s, 3H, 1-*H*) ppm.
This NMR data agrees with the data previously reported in literature.[12]
^{13}C{^{1}H} NMR (101 MHz, $CDCl_3$) δ = 149.33, 130.1, 127.5, 122.0, 37.3 ppm

1.15 *O*-Butyl methanesulfonate (3b)

According to GP1, methanesulfonic acid (MsOH) (340 mg, 3.54 mmol, 1 equiv.), tetrabutylammonium thiocyanate (NBu_4SCN) (180 mg, 0.5 mmol, 0.15 equiv.), triethylamine (1.5 mL, 10.6 mmol, 3 equiv.) and 1-butanol (7.08 mmol, 0.65 mL, 2 equiv.) were employed. The electrolysis was carried out using 3 *F*. After purification by column chromatography (cyclohexane/ethyl acetate = 9/1 → 8/2), the desired product **3b** (177 mg, 1.16 mmol, 66%) was obtained as a pale yellow oil.

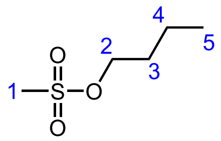

^{1}H NMR (400 MHz, $CDCl_3$) δ = 4.17 (t, *J* = 6.5 Hz, 2H, 2-*H*), 2.94 (s, 3H, 1-*H*), 1.67 (ddt, *J* = 8.9, 7.9, 6.5 Hz, 2H, 3-*H*), 1.45 – 1.27 (m, 2H, 4-*H*), 0.89 (t, *J* = 7.4 Hz, 3H, 5-*H*) ppm.
^{13}C{^{1}H} NMR (101 MHz, $CDCl_3$) δ = 70.0, 37.4, 31.2, 18.8, 13.6.ppm.
This NMR data agrees with the data previously reported in literature.[13]

1.16 *O*-Cyclohexyl methanesulfonate (3c)

According to GP1, methanesulfonic acid (MsOH) (340 mg, 3.54 mmol, 1 equiv.), tetrabutylammonium thiocyanate (NBu_4SCN) (180 mg, 0.5 mmol, 0.15 equiv.), triethylamine (1.5 mL, 10.6 mmol, 3 equiv.) and cyclohexanol (7.08 mmol, 0.74 mL, 2 equiv.) were employed. The electrolysis was carried out using 3 *F*. After purification by column chromatography (cyclohexane/ethyl acetate = 9/1 → 8/2), the desired product **3c** (182 mg, 1.03 mmol, 58%) was obtained as a clear oil

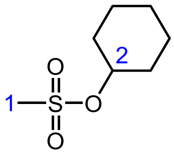

^{1}H NMR (400 MHz, CDCl$_3$) δ = 4.68 (tt, *J* = 9.1, 3.9 Hz, 1H, 2-*H*), 2.99 (s, 3H, , 1-*H*), 2.03 – 1.91 (m, 2H, CyHex-*H*), 1.83 – 1.71 (m, 2H, CyHex-*H*), 1.69 – 1.57 (m, 2H, CyHex-*H*), 1.57 – 1.47 (m, 1H, CyHex-*H*), 1.46 – 1.14 (m, 4H, CyHex-*H*) ppm.
^{13}C{^{1}H} NMR (101 MHz, CDCl$_3$) δ = 81.5, 38.9, 32.8, 24.9, 23.6 ppm.
This NMR data agrees with the data previously reported in literature.[13]

1.17 Cyclohex-3-en-1-yl methanesulfonate (3d)

According to GP1, methanesulfonic acid (MsOH) (340 mg, 3.54 mmol, 1 equiv.), tetrabutylammonium thiocyanate (NBu_4SCN) (180 mg, 0.5 mmol, 0.15 equiv.), triethylamine (1.5 mL, 10.6 mmol, 3 equiv.) and cyclohexen-3-ol (7.08 mmol, 0 86 mL, 2 equiv.) were employed. The electrolysis was carried out using 3 *F*. After purification by column chromatography (cyclohexane/ethyl acetate = 9/1 → 8/2), the desired product **3d** (202 mg, 1.15 mmol, 65%) was obtained as a colorless oil.

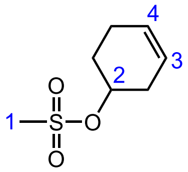

^{1}H NMR (400 MHz, CDCl$_3$) δ = 5.70 (ddt, *J* = 7.3, 3.5, 1.8 Hz, 1H, 3-*H*), 5.61 – 5.52 (m, 1H, 4-*H*), 4.96 (tq, *J* = 8.8, 3.2 Hz, 1H, 2-*H*), 3.02 (s, 3H, 1-*H*), 2.57 – 2.44 (m, 1H, CyHex-*H*), 2.40 – 2.10 (m, 3H, CyHex-*H*), 2.09 – 1.85 (m, 2H, CyHex-*H*) ppm.
^{13}C{^{1}H} NMR (101 MHz, CDCl$_3$) δ = 127.0, 122.8, 78.2, 38.9, 31.8, 28.5, 23.2 ppm.
HRMS (ESI, m/z): [M+Na]$^+$ = calcd. For $C_7H_{13}O_3SNa^+$: 199.03994, found: 199.03999

1.18 1,1-Dimethylethyl-4-((methylsulfonyl)oxy)piperidine-1-carboxylate (3e)

According to GP1, methanesulfonic acid (MsOH) (340 mg, 3.54 mmol, 1 equiv.), tetrabutylammonium thiocyanate (NBu_4SCN) (180 mg, 0.5 mmol, 0.15 equiv.), triethylamine (1.5 mL, 10.6 mmol, 3 equiv.) and 1-Boc-4-hydroxypiperidine (7.08 mmol, 1.4 g, 2 equiv.) were employed. The electrolysis was carried out using 3 *F*. After purification by column chromatography (cyclohexane/ethyl acetate = 9/1 → 6/4), the desired product **3e** (331 mg, 1.18 mmol, 67%) was obtained as an off-white solid.

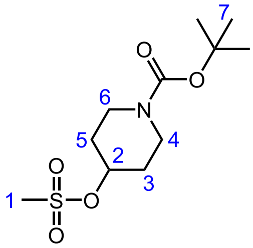

^{1}H NMR (400 MHz, CDCl$_3$) δ = 4.88 (tt, *J* = 7.8, 3.8 Hz, 1H, 2-*H*), 3.70 (ddd, *J* = 13.8, 7.0, 4.0 Hz, 2H, 3-*H*), 3.30 (ddd, *J* = 13.7, 8.1, 3.8 Hz, 2H, 5-*H*), 3.03 (s, 3H, 1-*H*), 1.96 (ddt, *J* = 10.9, 7.3, 3.9 Hz, 2H, 4-*H*), 1.81 (dtd, *J* = 12.3, 7.9, 4.0 Hz, 2H, 6-*H*), 1.46 (s, 9H, 7-*H*) ppm.
^{13}C{^{1}H} NMR (101 MHz, CDCl$_3$) δ = 154.7, 80.1, 77.8, 39.0, 31.8, 28.5 ppm.
This NMR data agrees with the data previously reported in literature.[14]

1.19 Methyl (S)-2-((methylsulfonyl)oxy)propanoate (3f)

According to GP1, methanesulfonic acid (MsOH) (340 mg, 3.54 mmol, 1 equiv.), tetrabutylammonium thiocyanate (NBu_4SCN) (180 mg, 0.5 mmol, 0.15 equiv.), triethylamine (1.5 mL, 10.6 mmol, 3 equiv.) and (–)methyl L-lactate (7.08 mmol, 0.74 mL, 2 equiv.) were employed. The electrolysis was carried out using 3 *F*. After purification by column chromatography (cyclohexane/ethyl acetate = 9/1 → 8/2), the desired product **3f** (201 mg, 1.11 mmol, 63%) was obtained as a clear oil.

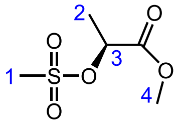

^{1}H NMR (400 MHz, CDCl$_3$) δ = 5.14 (q, *J* = 7.0 Hz, 1H, 3-*H*), 3.80 (s, 3H, 4-*H*), 3.15 (s, 3H, 1-*H*), 1.62 (d, *J* = 7.0 Hz, 3H, 2-*H*) ppm.
^{13}C{^{1}H} NMR (101 MHz, CDCl$_3$) δ = 170.1, 74.2, 53.0, 39.3, 18.5 ppm.
This NMR data agrees with the data previously reported in literature.[15]

1.20 1-(Phenylsulfonyl)pyrrolidine (4a)

According to GP1, benzenesulfonic acid (340 mg, 3.54 mmol, 1 equiv.), tetrabutylammonium thiocyanate (NBu_4SCN) (180 mg, 0.5 mmol, 0.15 equiv.), and pyrrolidine (17.7 mmol, 1.5 mL, 5 equiv.) were employed. The electrolysis was carried out using 3 *F*. After purification by column chromatography (cyclohexane/ethyl acetate = 9/1 → 6/4), the desired product **4a** (82 mg, 0.39 mmol, 22%) was obtained as a pale yellow viscous foam.

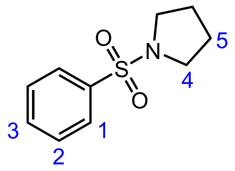

^{1}H NMR (400 MHz, CDCl$_3$) δ = 7.87 – 7.81 (m, 2H, 1-*H*), 7.64 – 7.57 (m, 1H, 3-*H*), 7.56 – 7.49 (m, 2H, 2-*H*), 3.31 – 3.19 (m, 4H, 4-*H*), 1.82 – 1.70 (m, 4H, 5-*H*) ppm.
^{13}C{^{1}H} NMR (101 MHz, CDCl$_3$) δ = 137.1, 132.7, 129.1, 127.6, 48.1, 25.4 ppm.
This NMR data agrees with the data previously reported in literature.[6,8,16]

1.21 1-Tosyl pyrrolidine (4b)

According to GP1, p-toluensulfonic acid (340 mg, 3.54 mmol, 1 equiv.), tetrabutylammonium thiocyanate (NBu$_4$SCN) (180 mg, 0.5 mmol, 0.15 equiv.), and pyrrolidine (17.7 mmol, 1.5 mL, 5 equiv.) were employed. The electrolysis was carried out using 3 *F*. After purification by column chromatography (cyclohexane/ethyl acetate = 9/1 → 6/4), the desired product **4b** (31 mg, 0.14 mmol, 8%) was obtained as a pale yellow oil.

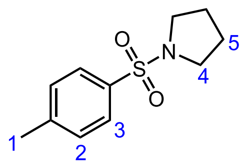

^{1}H NMR (400 MHz, CDCl$_3$) δ = 7.72 (d, *J* = 8.3 Hz, 2H, 3-*H*), 7.32 (d, *J* = 8.0 Hz, 2H, 2-*H*), 3.29 – 3.18 (m, 4H, 4-*H*), 2.43 (s, 3H, 1-*H*), 1.80 – 1.70 (m, 4H, 5-*H*) ppm.
^{13}C NMR couldn't be measured because of the low quantity resulted in bad signal to noise ration in the spectrum
This NMR data agrees with the data previously reported in literature.[6,17]

1.22 1-((4-Bromophenyl)sulfonyl)pyrrolidine (4c)

According to GP1, 4-bromobenzenesulfonic acid (340 mg, 3.54 mmol, 1 equiv.), tetrabutylammonium thiocyanate (NBu$_4$SCN) (180 mg, 0.5 mmol, 0.15 equiv.), and pyrrolidine (17.7 mmol, 1.5 mL, 5 equiv.) were employed. The electrolysis was carried out using 3 *F*. After purification by column chromatography (cyclohexane/ethyl acetate = 9/1 → 6/4), the desired product **4c** (56 mg, 0.19 mmol, 11%) was obtained as a brown oil.

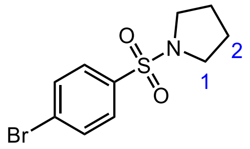

^{1}H NMR (400 MHz, CDCl$_3$) δ = 7.72 – 7.60 (m, 4H, Ar-*H*), 3.27 – 3.16 (m, 4H, 1-*H*), 1.82 – 1.69 (m, 4H, 2-*H*) ppm.
^{13}C{^{1}H} NMR (101 MHz, CDCl$_3$) δ = 136.1, 132.4, 129.0, 127.6, 48.0, 25.3 ppm.
This NMR data agrees with the data previously reported in literature.[6,8]

1.23 1-((4-Chlorophenyl)sulfonyl)pyrrolidine (4d)

According to GP1, 4-chlorobenzenesulfonic acid (340 mg, 3.54 mmol, 1 equiv.), tetrabutylammonium thiocyanate (NBu$_4$SCN) (180 mg, 0.5 mmol, 0.15 equiv.), and pyrrolidine (17.7 mmol, 1.5 mL, 5 equiv.) were employed. The electrolysis was carried out using 3 *F*. After purification by column chromatography (cyclohexane/ethyl acetate = 9/1 → 6/4), the desired product **4d** (65 mg, 0.26 mmol, 15%) was obtained as a pale yellow oil.

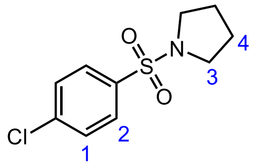

^{1}H NMR (400 MHz, CDCl$_3$) δ = 7.81 – 7.74 (m, 2H, 2-*H*), 7.54 – 7.48 (m, 2H, 1-*H*), 3.30 – 3.19 (m, 4H, 3-*H*), 1.85 – 1.72 (m, 4H, 4-*H*) ppm.
^{13}C{^{1}H} NMR (101 MHz, CDCl$_3$) δ = 139.2, 135.7, 129.5, 129.0, 48.1, 25.4 ppm.
This NMR data agrees with the data previously reported in literature.[6,8]

1.24 1-((4-Methoxyphenyl)sulfonyl)pyrrolidine (4e)

According to GP1, 4-methoxybenzenesulfonic acid (340 mg, 3.54 mmol, 1 equiv.), tetrabutylammonium thiocyanate (NBu$_4$SCN) (180 mg, 0.5 mmol, 0.15 equiv.), and pyrrolidine (17.7 mmol, 1.5 mL, 5 equiv.) were employed. The electrolysis was carried out using 3 *F*. After purification by column chromatography (cyclohexane/ethyl acetate = 9/1 → 6/4), the desired product **4e** (123 mg, 0.51 mmol, 29%) was obtained as a pale yellow oil.

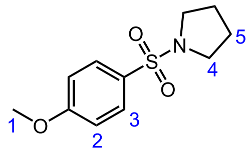

^{1}H NMR (400 MHz, CDCl$_3$) δ = 7.84 – 7.75 (m, 2H, 3-*H*), 7.06 – 6.97 (m, 2H, 2-*H*), 3.90 (s, 3H, 1-*H*), 3.34 – 3.18 (m, 4H, 4-*H*), 1.86 – 1.72 (m, 4H, 5-*H*) ppm.
^{13}C{^{1}H} NMR (101 MHz, CDCl$_3$) δ = 162.9, 129.6, 128.8, 114.1, 55.6, 47.9, 25.2 ppm.
This NMR data agrees with the data previously reported in literature.[18]

1.25 *N*-Phenyl ethanesulfonamide (4f)

According to GP1, ethanesulfonic acid (340 mg, 3.54 mmol, 1 equiv.), tetrabutylammonium thiocyanate (NBu_4SCN) (180 mg, 0.5 mmol, 0.15 equiv.), and aniline (17.7 mmol, 1.6 mL, 5 equiv.) were employed. The electrolysis was carried out using 3 *F*. After purification by column chromatography (cyclohexane/ethyl acetate = 9/1 → 7/3), the desired product **4f** (199 mg, 1.08 mmol, 61%) was obtained as a colorless viscous oil.

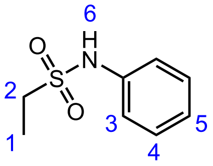

^{1}H NMR (400 MHz, $CDCl_3$) δ = 7.41 – 7.33 (m, 2H, 3-*H*), 7.30 – 7.22 (m, 2H, 4-*H*), 7.19 (td, *J* = 7.3, 1.2 Hz, 1H, 5-*H*), 6.82 (s, 1H, 6-*H*), 3.16 (q, *J* = 7.4 Hz, 2H, 2-*H*), 1.40 (t, *J* = 7.4 Hz, 3H, 1-*H*) ppm.
^{13}C{^{1}H} NMR (101 MHz, $CDCl_3$) δ = 137.0, 129.8, 125.3, 120.6, 46.0, 8.3 ppm.
This NMR data agrees with the data previously reported in literature.[19]

1.26 *N*-Phenyl propanesulfonamide (4g)

According to GP1, propanesulfonic acid (340 mg, 3.54 mmol, 1 equiv.), tetrabutylammonium thiocyanate (NBu_4SCN) (180 mg, 0.5 mmol, 0.15 equiv.), and aniline (17.7 mmol, 1.6 mL, 5 equiv.) were employed. The electrolysis was carried out using 3 *F*. After purification by column chromatography (cyclohexane/ethyl acetate = 9/1 → 6/4), the desired product **4g** (193 mg, 0.97 mmol, 55%) was obtained as a yellow oil.

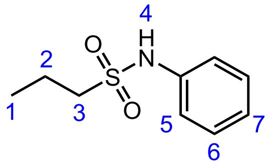

^{1}H NMR (400 MHz, $CDCl_3$) δ = 7.27 (dd, *J* = 8.5, 7.3 Hz, 2H, 5-*H*), 7.18 – 7.02 (m, 3H, 6-7-*H*), 6.69 (s, 1H, 4-*H*), 3.08 – 2.93 (m, 2H, 3-*H*), 1.88 – 1.71 (m, 2H, 2-*H*), 0.95 (t, *J* = 7.4 Hz, 3H, 1-*H*) ppm.
This NMR data agrees with the data previously reported in literature.[20]
^{13}C{^{1}H} NMR (101 MHz, $CDCl_3$) δ = 136.9, 129.7, 125.1, 120.4, 53.4, 17.3, 12.9 ppm.

1.27 *N*-Phenyl-2,2,2-trifluoroethanesulfonamide (4h)

According to GP1, 2,2,2-trifluoroethanesulfonic acid (340 mg, 3.54 mmol, 1 equiv.), tetrabutylammonium thiocyanate (NBu_4SCN) (180 mg, 0.5 mmol, 0.15 equiv.), and aniline (17.7 mmol, 1.6 mL, 5 equiv.) were employed. The electrolysis was carried out using 3 *F*. After purification by column chromatography (cyclohexane/ethyl acetate = 9/1 → 6/4), the desired product **4h** (215 mg, 0.90 mmol, 51%) was obtained as a colorless oil.

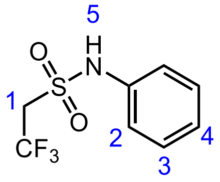

^{1}H NMR (400 MHz, $CDCl_3$) δ = 7.33 (dd, *J* = 8.7, 7.0 Hz, 2H, 2-H), 7.20 (ddd, *J* = 8.4, 7.3, 2.0 Hz, 3H, 3-4-H), 6.74 (s, 1H, 5-H), 3.73 (q, *J* = 8.9 Hz, 2H, 1-H) ppm.
^{13}C{^{1}H} NMR (101 MHz, $CDCl_3$) δ = 134.9, 129.8, 126.5, 122.6, 121.7, 119.8, 52.3, 52.0, 51.7, 51.3 ppm.
^{19}F NMR (376 MHz, $CDCl_3$) δ = -62.27 ppm.
HRMS (ESI, m/z): $[M+H]^+$ = calcd. For $C_8H_9F_3NO_2S^+$: 240.0301, found: 240.0304

1.28 4-Cyano-*N*-phenylbenzenesulfonamide (4i)

According to GP1, 4-cyanobenzenesulfonic acid (340 mg, 3.54 mmol, 1 equiv.), tetrabutylammonium thiocyanate (NBu_4SCN) (180 mg, 0.5 mmol, 0.15 equiv.), and aniline (17.7 mmol, 1.6 mL, 5 equiv.) were employed. The electrolysis was carried out using 3 *F*. After purification by column chromatography (cyclohexane/ethyl acetate = 9/1 → 6/4), the desired product **4i** (100 mg, 0.39 mmol, 22%) was obtained as an off-white foam.

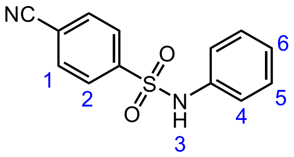

^{1}H NMR (400 MHz, $CDCl_3$) δ = 7.84 – 7.74 (m, 2H, 2-*H*), 7.66 (d, *J* = 8.5 Hz, 2H, 1-*H*), 7.20 (dd, *J* = 6.7, 1.4 Hz, 2H, 4-*H*), 7.16 – 7.07 (m, 1H, 6-*H*), 7.03 – 6.96 (m, 2H, 5-*H*), 6.78 (s, 1H, 3-*H*) ppm.
^{13}C{^{1}H} NMR (101 MHz, $CDCl_3$) δ = 143.3, 135.5, 133.0, 129.8, 128.0, 126.6, 122.5, 117.3, 116.9 ppm.
This NMR data agrees with the data previously reported in literature.[21]

1.29 *N*-Phenyl 1-methylethanesulfonamide (4j)

According to GP1, isopropanesulfonic acid (340 mg, 3.54 mmol, 1 equiv.), tetrabutylammonium thiocyanate (NBu_4SCN) (180 mg, 0.5 mmol, 0.15 equiv.), and aniline (17.7 mmol, 1.6 mL, 5 equiv.) were employed. The electrolysis was carried out using 3 *F*. After purification by column chromatography (cyclohexane/ethyl acetate = 9/1 → 6/4), the desired product **4j** (225 mg, 1.13 mmol, 64%) was obtained as a yellow oil.

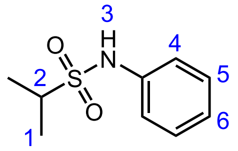

^{1}H NMR (400 MHz, $CDCl_3$) δ = 7.30 – 7.23 (m, 2H, 4-*H*), 7.18 – 7.12 (m, 2H, 5-*H*), 7.09 (td, *J* = 7.3, 1.3 Hz, 1H, 6-*H*), 3.23 (hept, *J* = 6.8 Hz, 1H, 2-*H*), 1.33 (d, *J* = 6.9 Hz, 6H, 1-*H*) ppm.
^{13}C{^{1}H} NMR (101 MHz, $CDCl_3$) δ = 137.0, 129.7, 125.0, 120.3, 52.5, 16.5 ppm.
This NMR data agrees with the data previously reported in literature.[22]

1.30 *N*-Phenyl phenylmethanesulfonamide (4k)

According to GP1, phenylmethanesulfonic acid (340 mg, 3.54 mmol, 1 equiv.), tetrabutylammonium thiocyanate (NBu_4SCN) (180 mg, 0.5 mmol, 0.15 equiv.), and aniline (17.7 mmol, 1.6 mL, 5 equiv.) were employed. The electrolysis was carried out using 3 *F*. After purification by column chromatography (cyclohexane/ethyl acetate = 9/1 → 6/4), the desired product **4k** (65 mg, 0.26 mmol, 15%) was obtained as a dark oil.

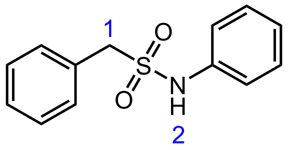

^{1}H NMR (400 MHz, $CDCl_3$) δ = 7.37 (ddd, *J* = 7.2, 5.0, 3.8 Hz, 5H, Ar-*H*), 7.31 – 7.24 (m, 2H, Ar-*H*), 7.23 – 7.14 (m, 3H, Ar-*H*), 6.60 – 6.46 (m, 1H, 2-*H*), 4.35 (s, 2H, 1-*H*) ppm.
^{13}C{^{1}H} NMR (101 MHz, $CDCl_3$) δ = 137.1, 131.0, 129.8, 129.1, 129.0, 128. 7, 125.0, 120.0, 57.6, 27.1 ppm.
This NMR data agrees with the data previously reported in literature.[23]

1.31 Limitations of the Scope/Unsuccessful Substrates

The following substrates were also employed, but the desired products could not be isolated in sufficient quantities.

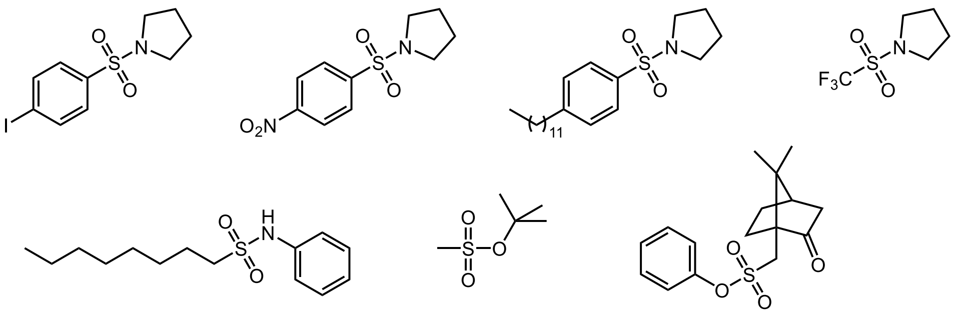

Figure S7 : Unsuccessful substrates.

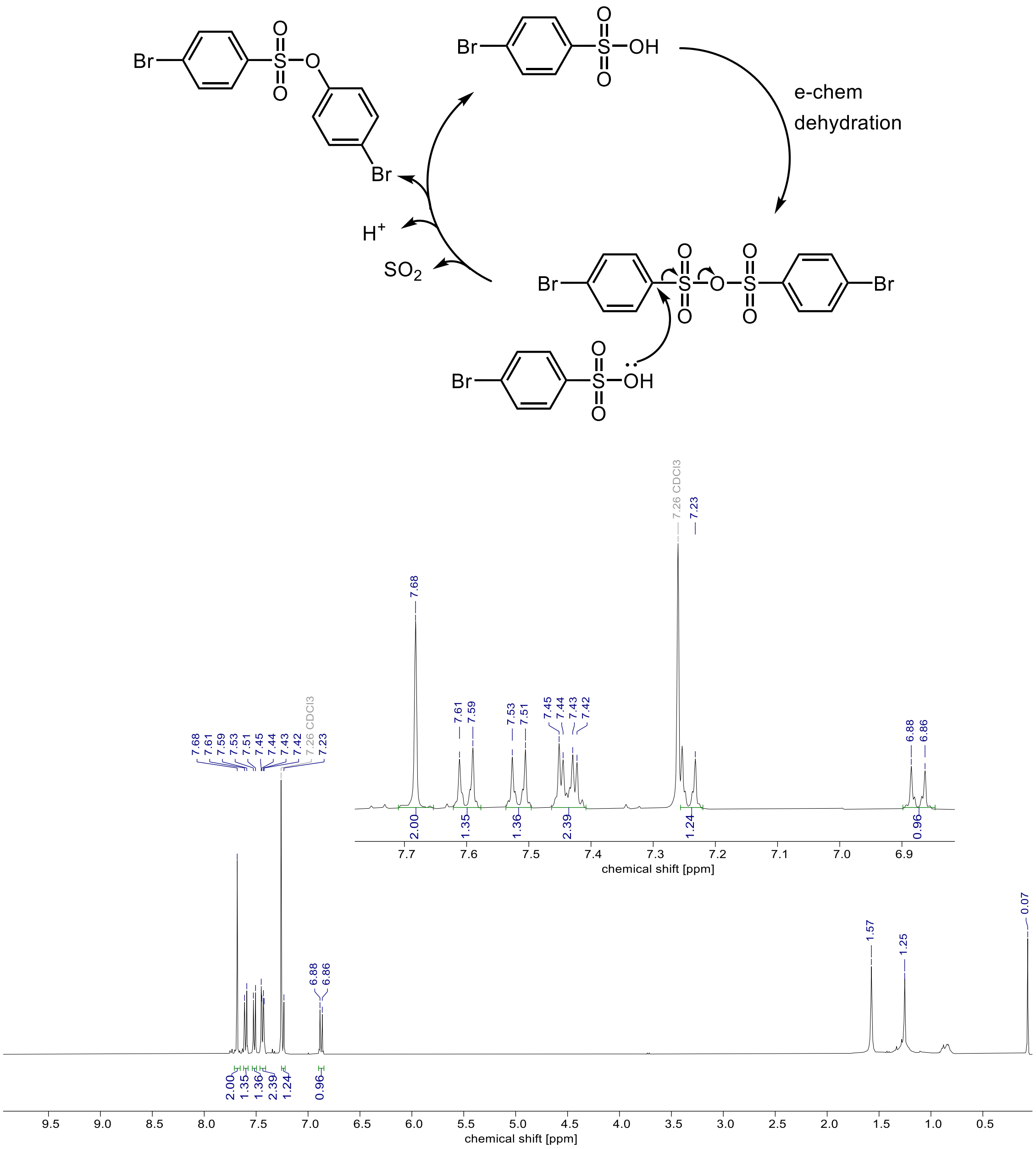

Figure S8: Proposed mechanism and ^{1}H-NMR spectrum of the ipso-substitution side products.

NMR Spectra

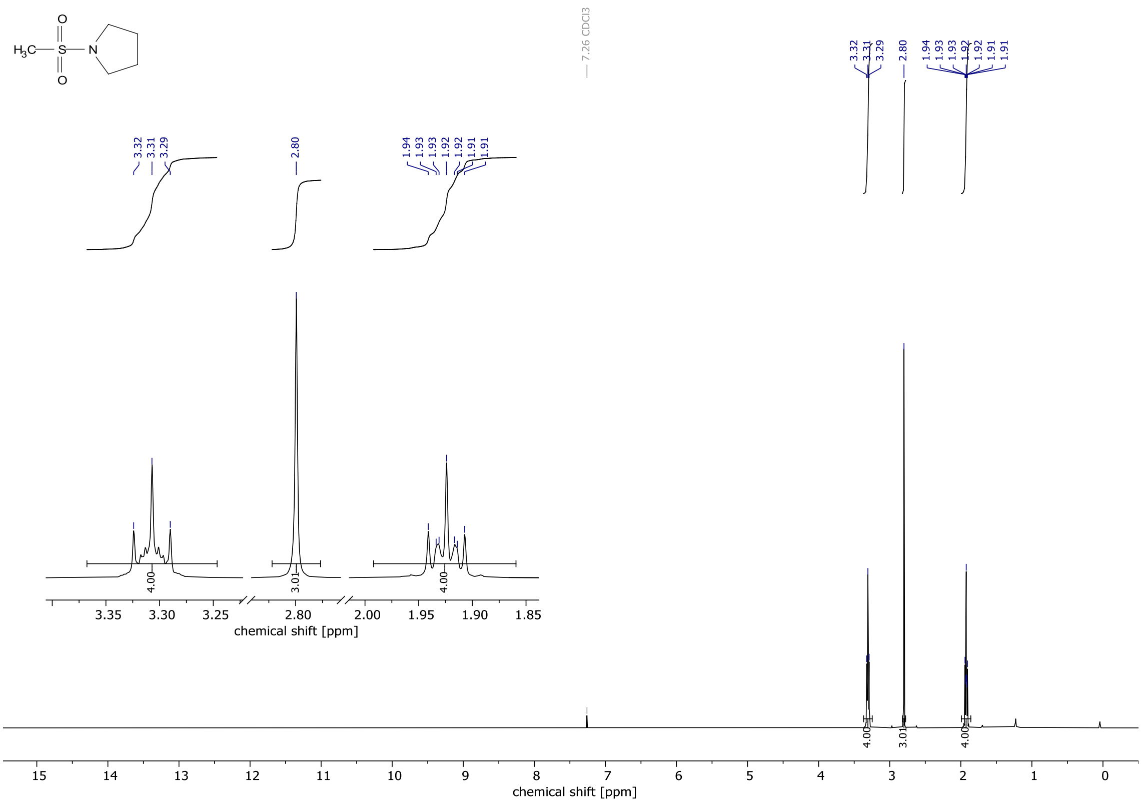

Figure S9: ^{1}H NMR spectrum (400 MHz, 25 °C, $CDCl_3$) of **2a**.

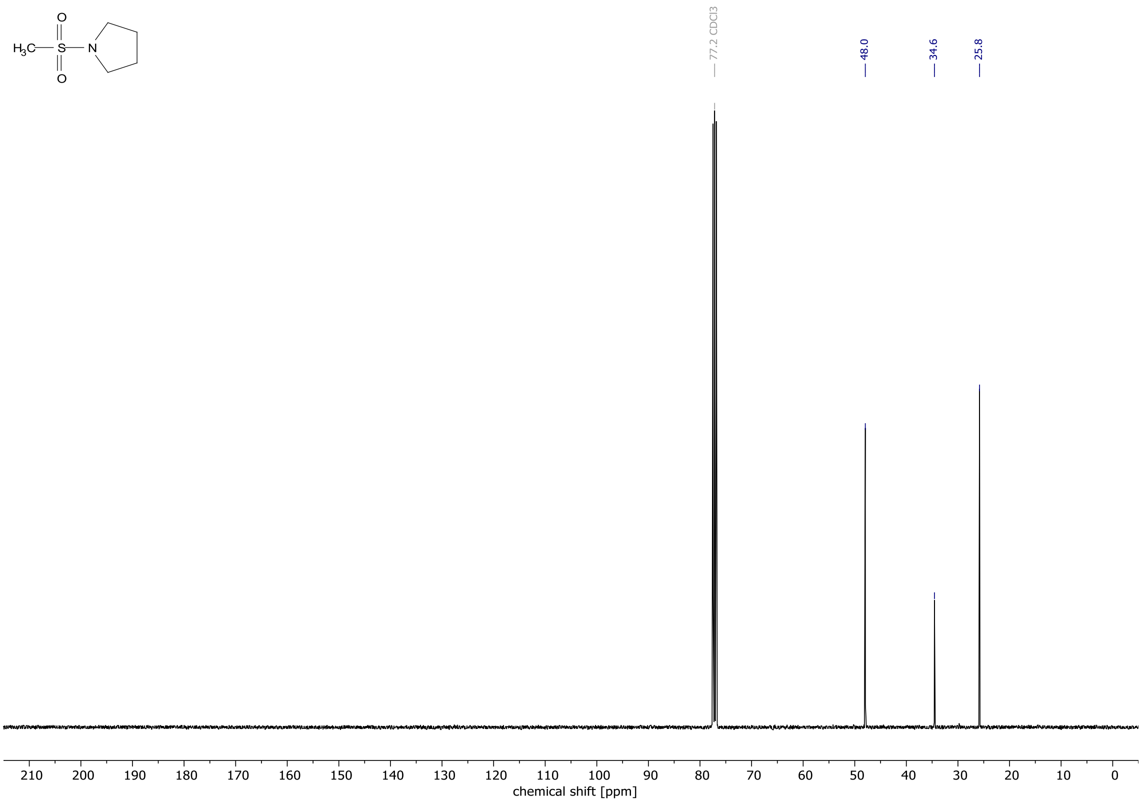

Figure S10: ^{13}C{^{1}H} NMR spectrum (101 MHz, 25 °C, $CDCl_3$) of **2a**.

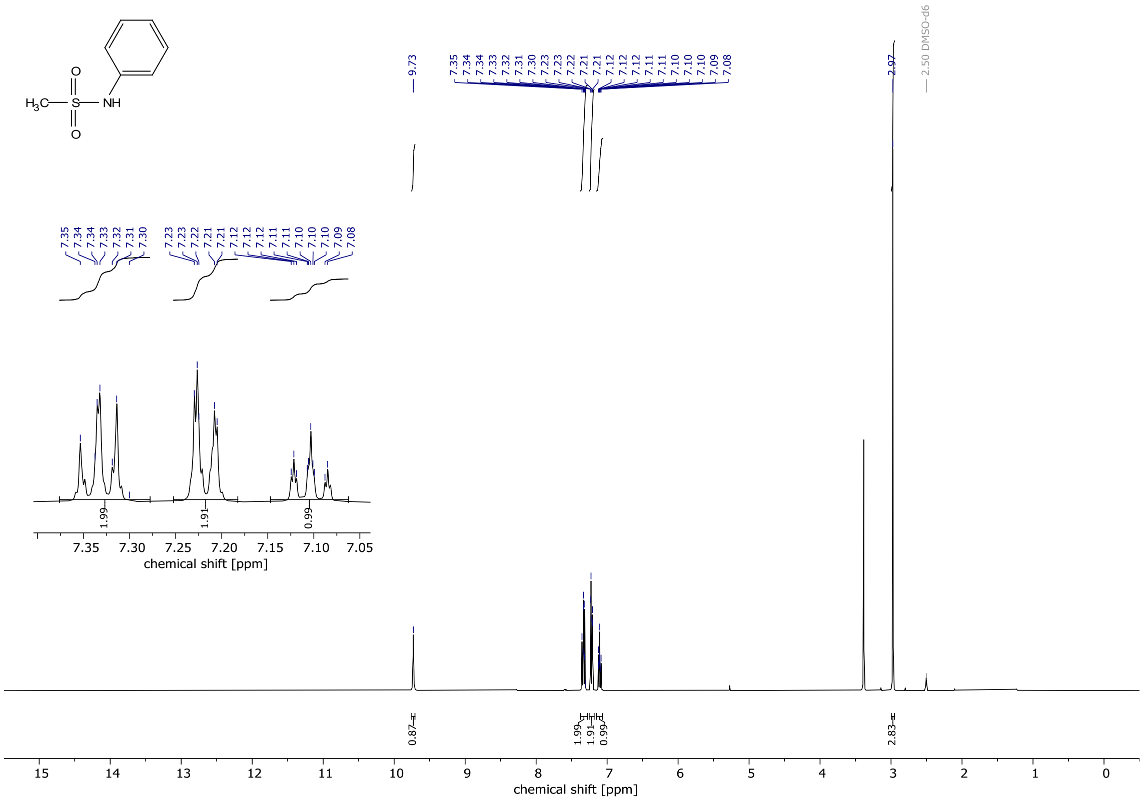

Figure S11: ^{1}H NMR spectrum (400 MHz, 25 °C, DMSO-d^6) of **2b**.

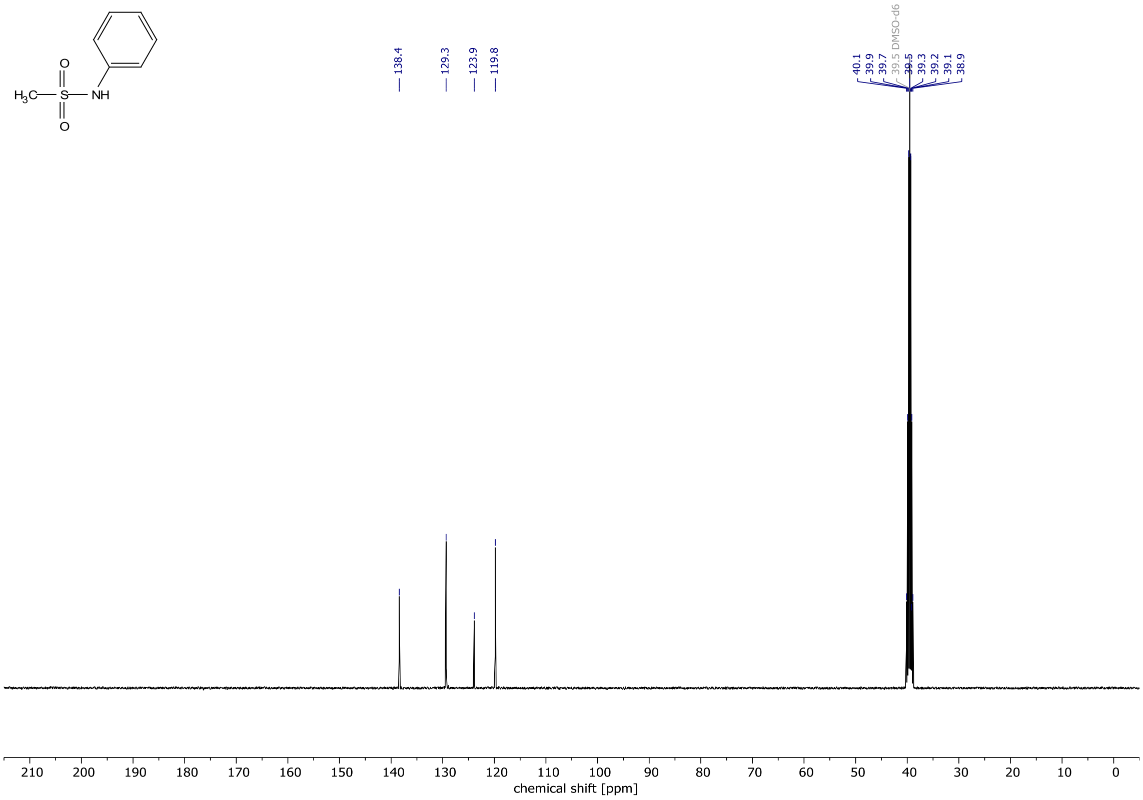

Figure S12: $^{13}C\{^{1}H\}$ NMR spectrum (101 MHz, 25 °C, DMSO-d^6) of **2b**.

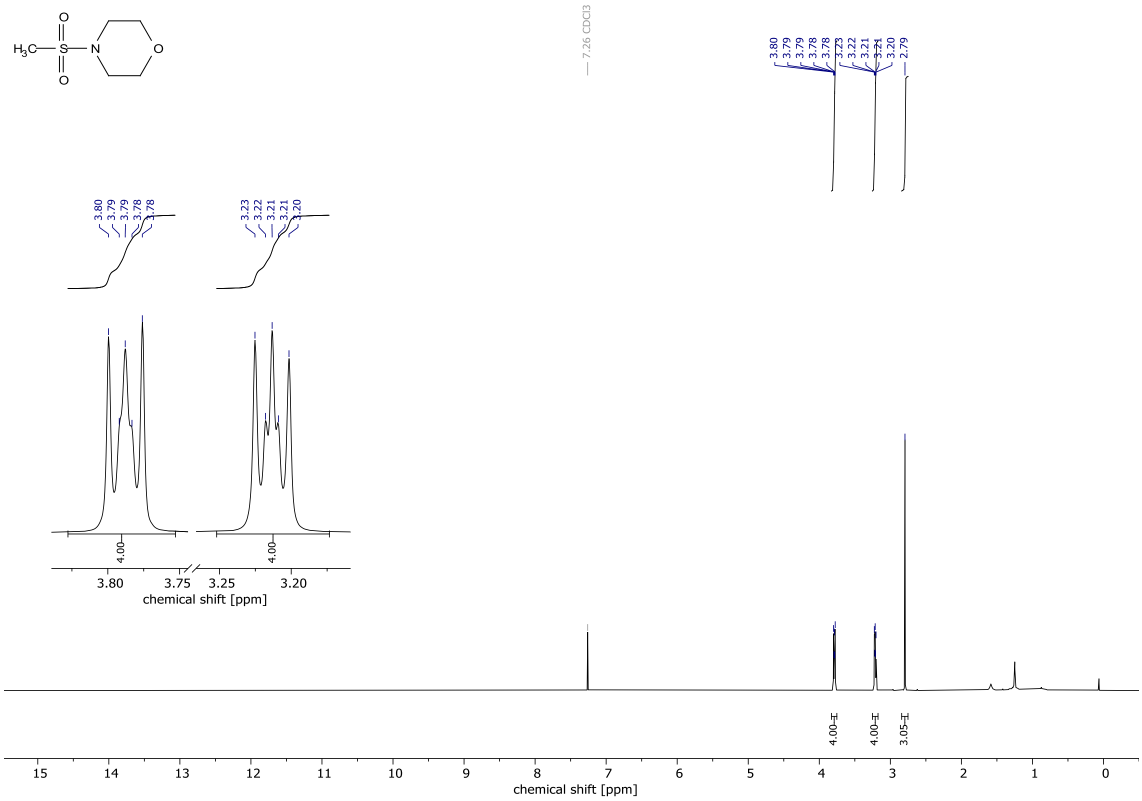

Figure S13: ^{1}H NMR spectrum (400 MHz, 25 °C, $CDCl_3$) of **2c**.

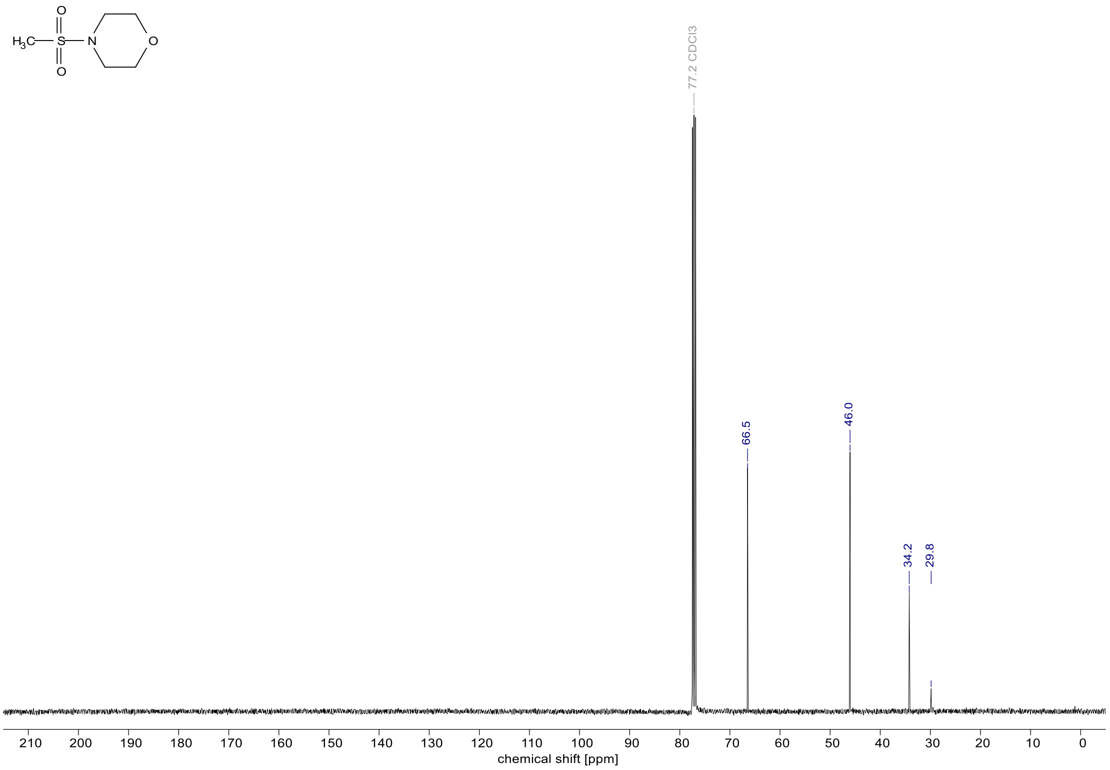

Figure S14: $^{13}C\{^{1}H\}$ NMR spectrum (101 MHz, 25 °C, $CDCl_3$) of **2c**.

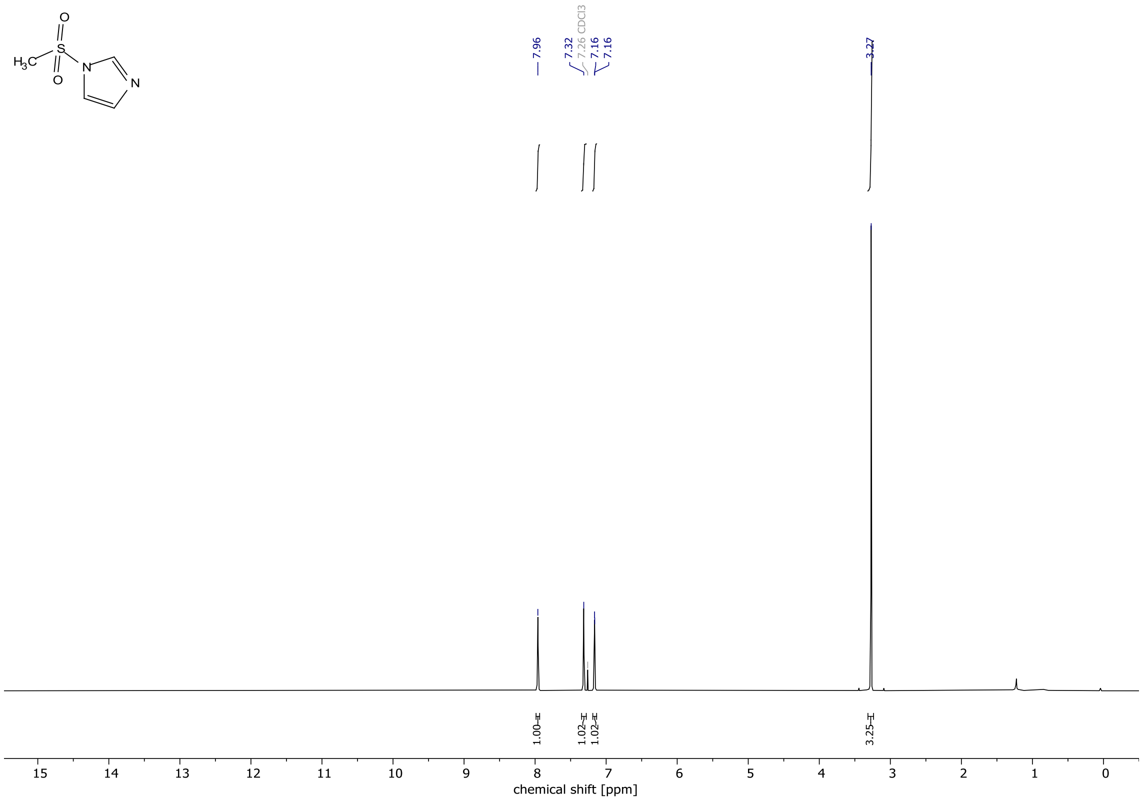

Figure S15: ^{1}H NMR spectrum (400 MHz, 25 °C, $CDCl_3$) of **2d**.

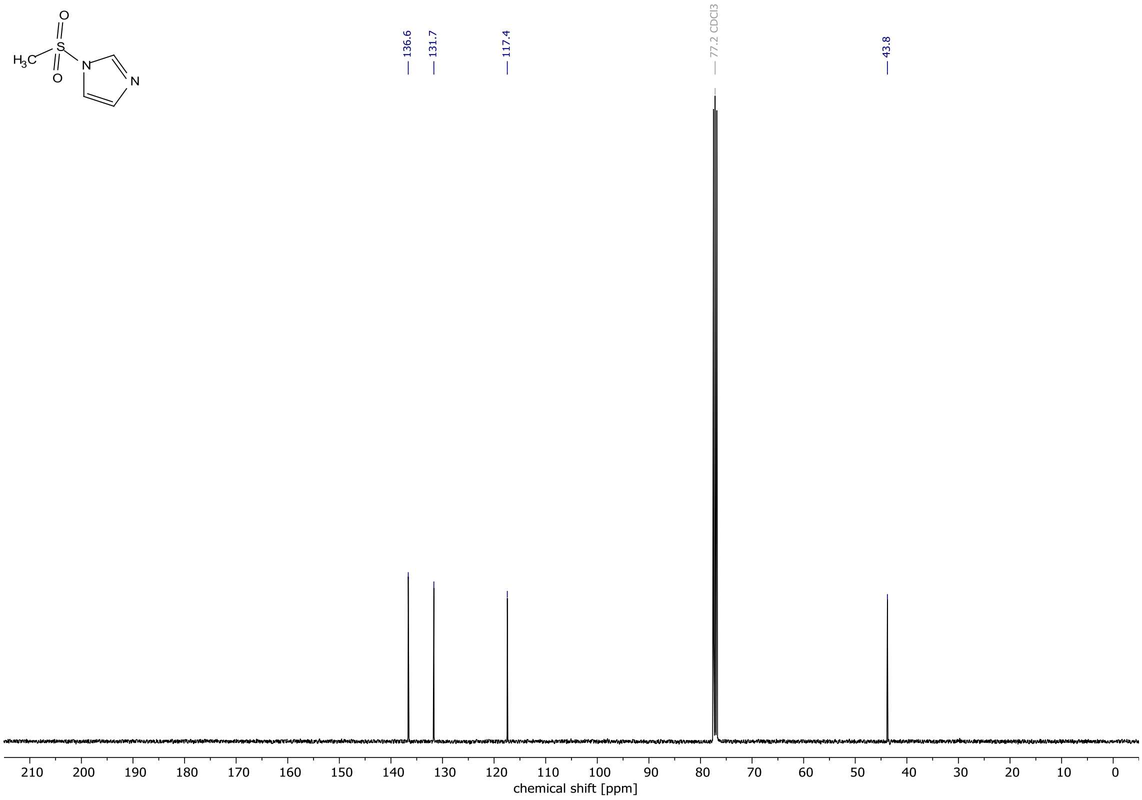

Figure S16: $^{13}C\{^{1}H\}$ NMR spectrum (101 MHz, 25 °C, $CDCl_3$) of **2d**.

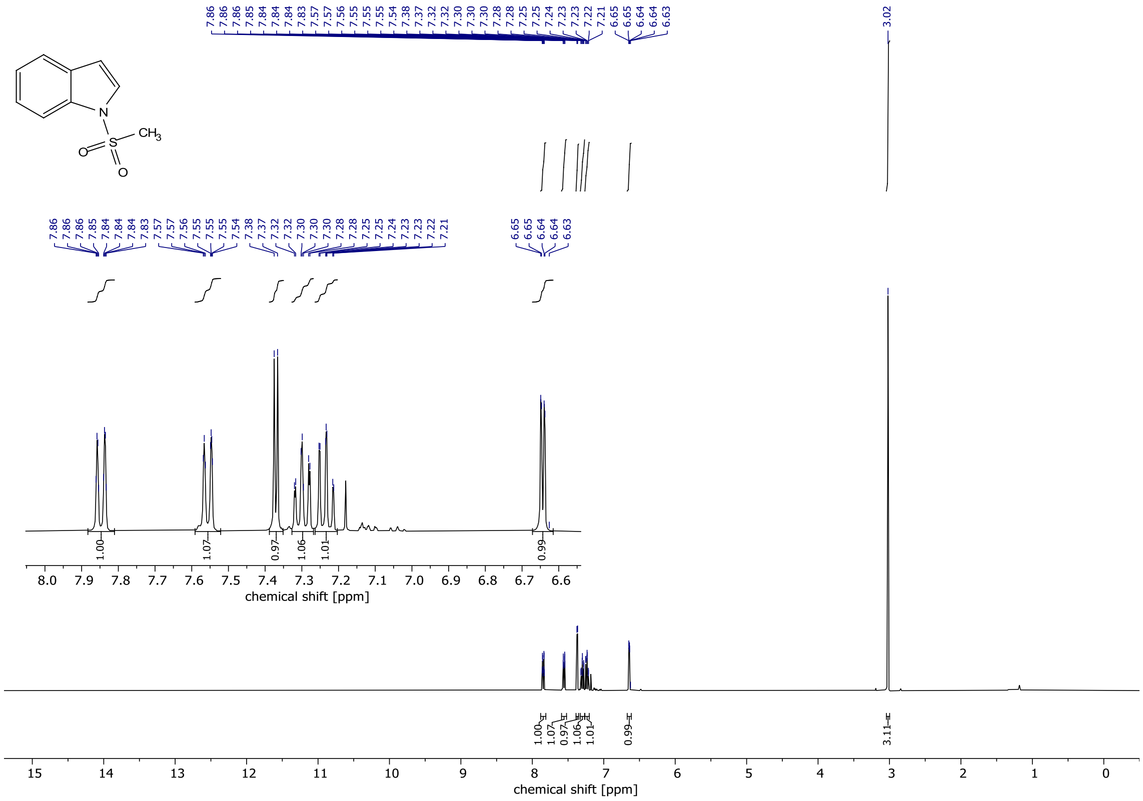

Figure S17: ^{1}H NMR spectrum (400 MHz, 25 °C, $CDCl_3$) of **2e**.

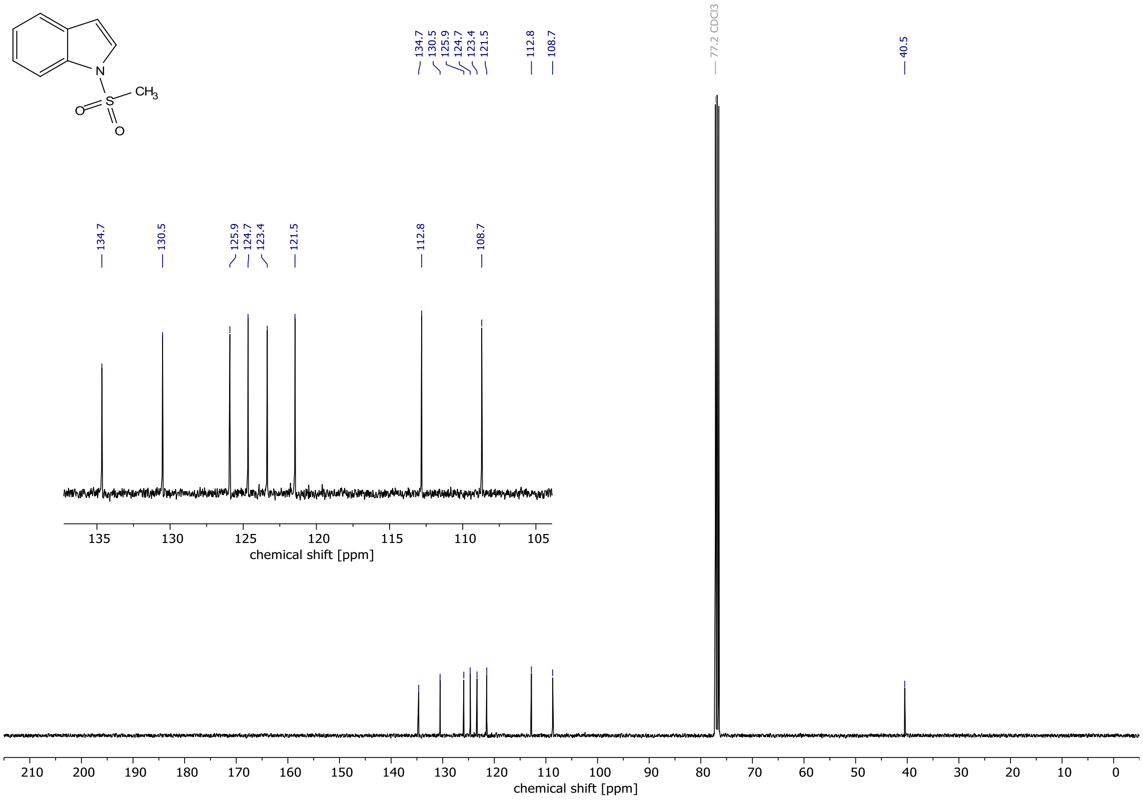

Figure S18: $^{13}C\{^{1}H\}$ NMR spectrum (101 MHz, 25 °C, $CDCl_3$) of **2e**.

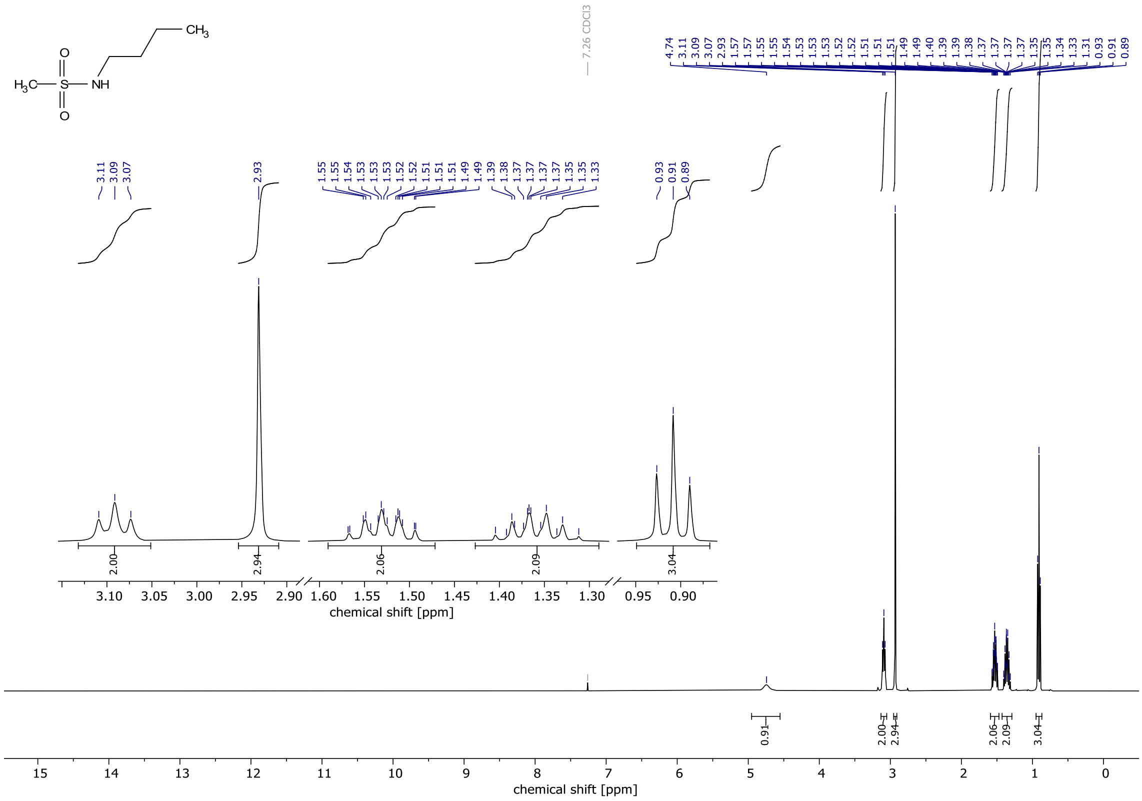

Figure S19: ^{1}H NMR spectrum (400 MHz, 25 °C, $CDCl_3$) of **2f**.

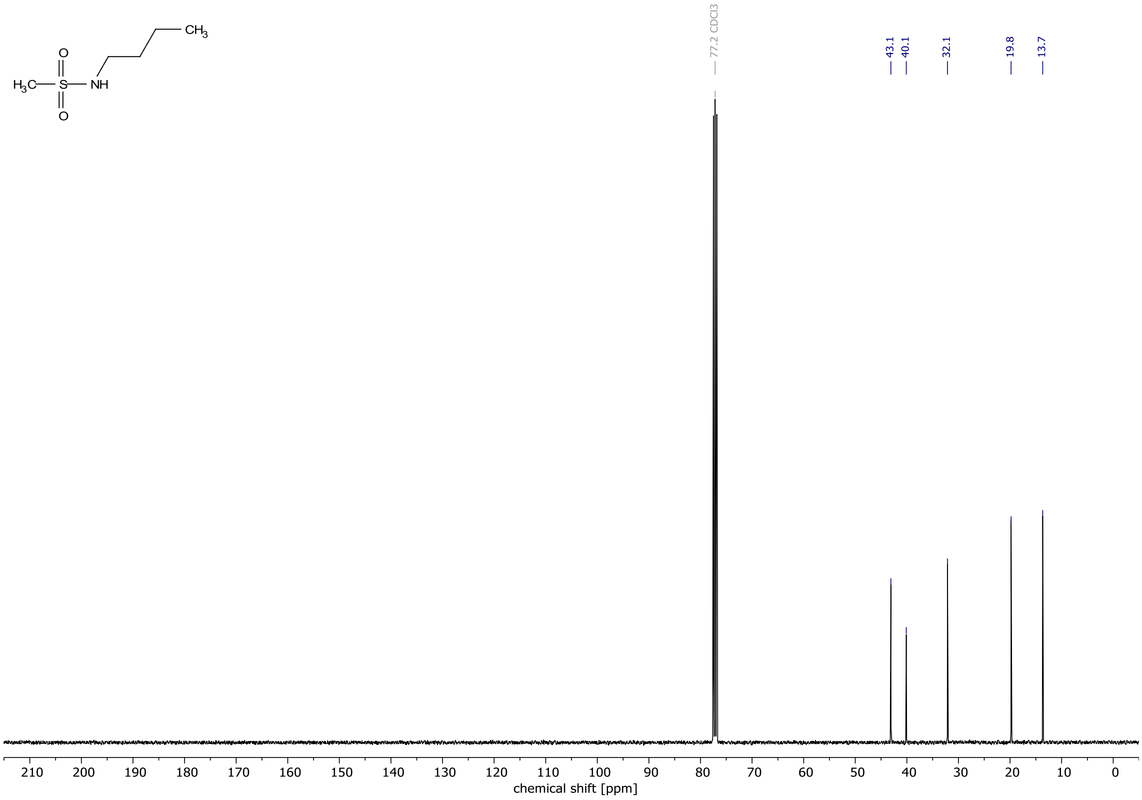

Figure S20: $^{13}C\{^{1}H\}$ NMR spectrum (101 MHz, 25 °C, $CDCl_3$) of **2f**.

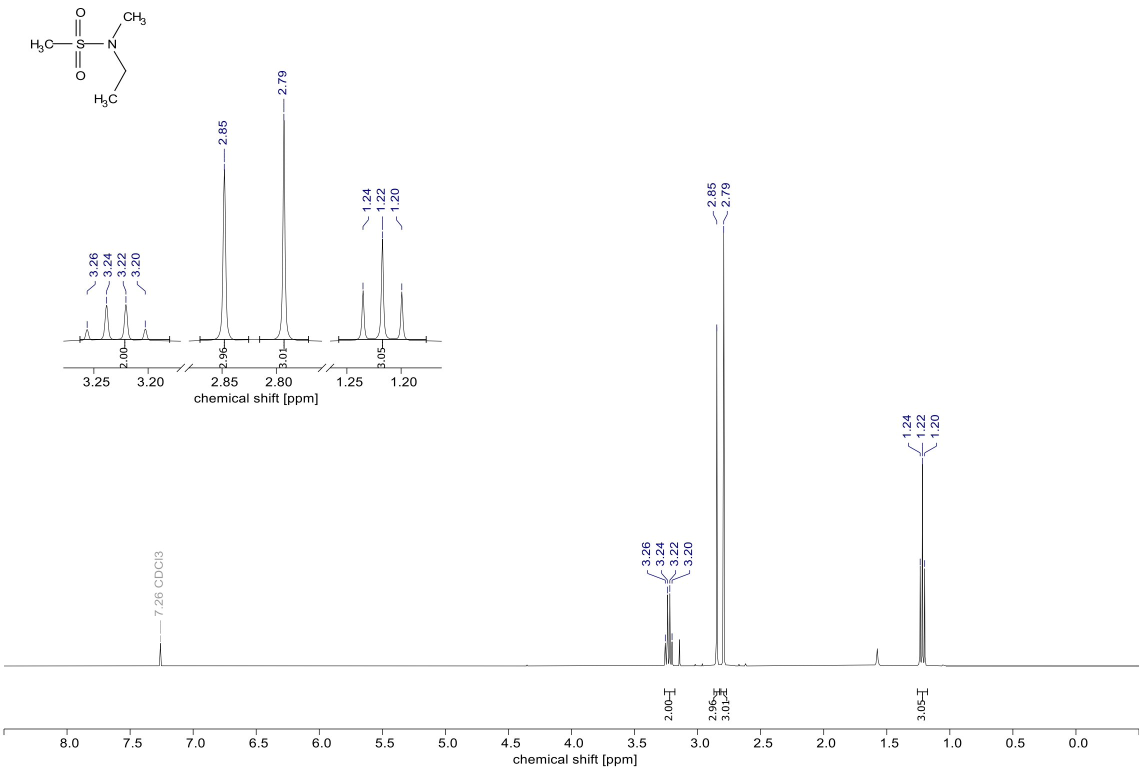

Figure S21: ^{1}H NMR spectrum (400 MHz, 25 °C, $CDCl_3$) of **2g**.

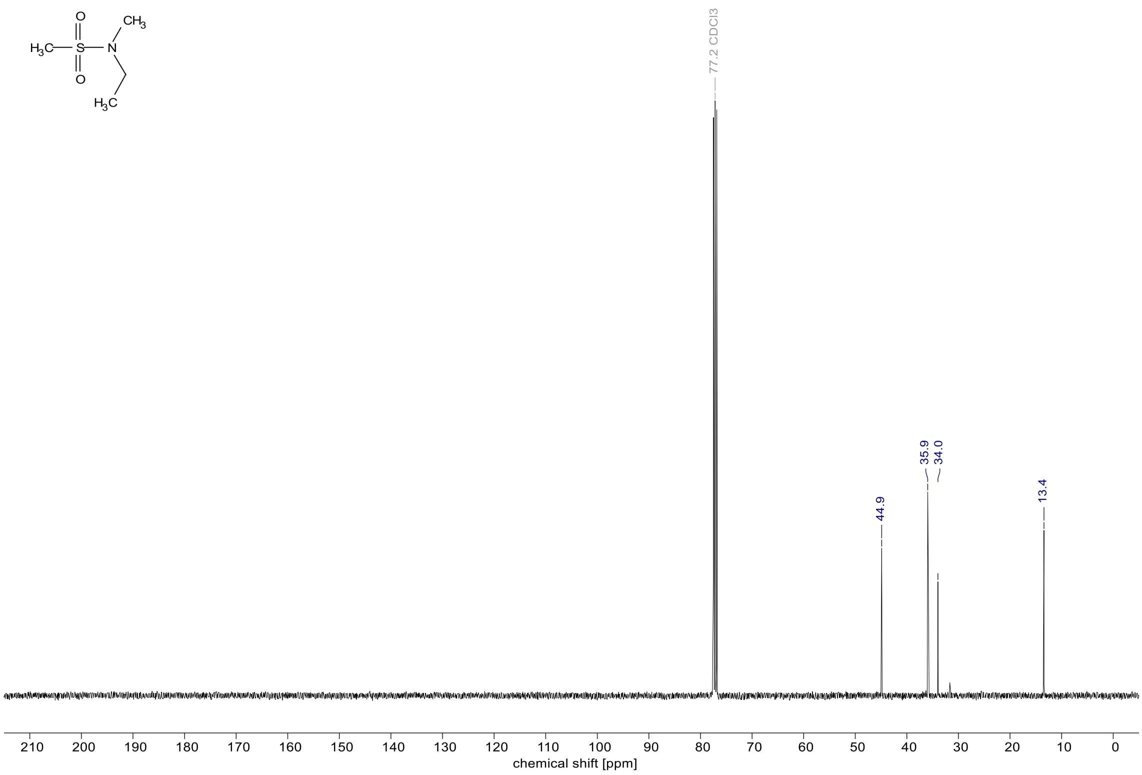

Figure S22: $^{13}C\{^1H\}$ NMR spectrum (101 MHz, 25 °C, $CDCl_3$) of **2g**.

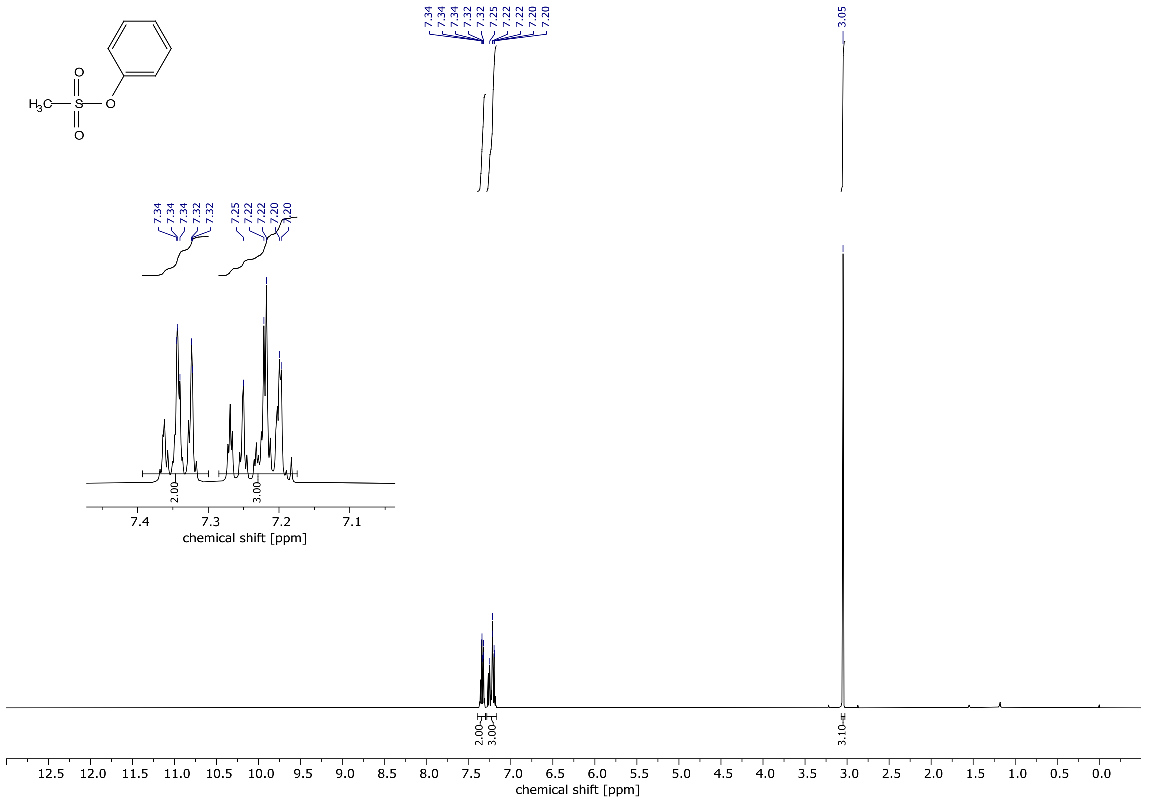

Figure S23: ^{1}H NMR spectrum (400 MHz, 25 °C, $CDCl_3$) of **3a**.

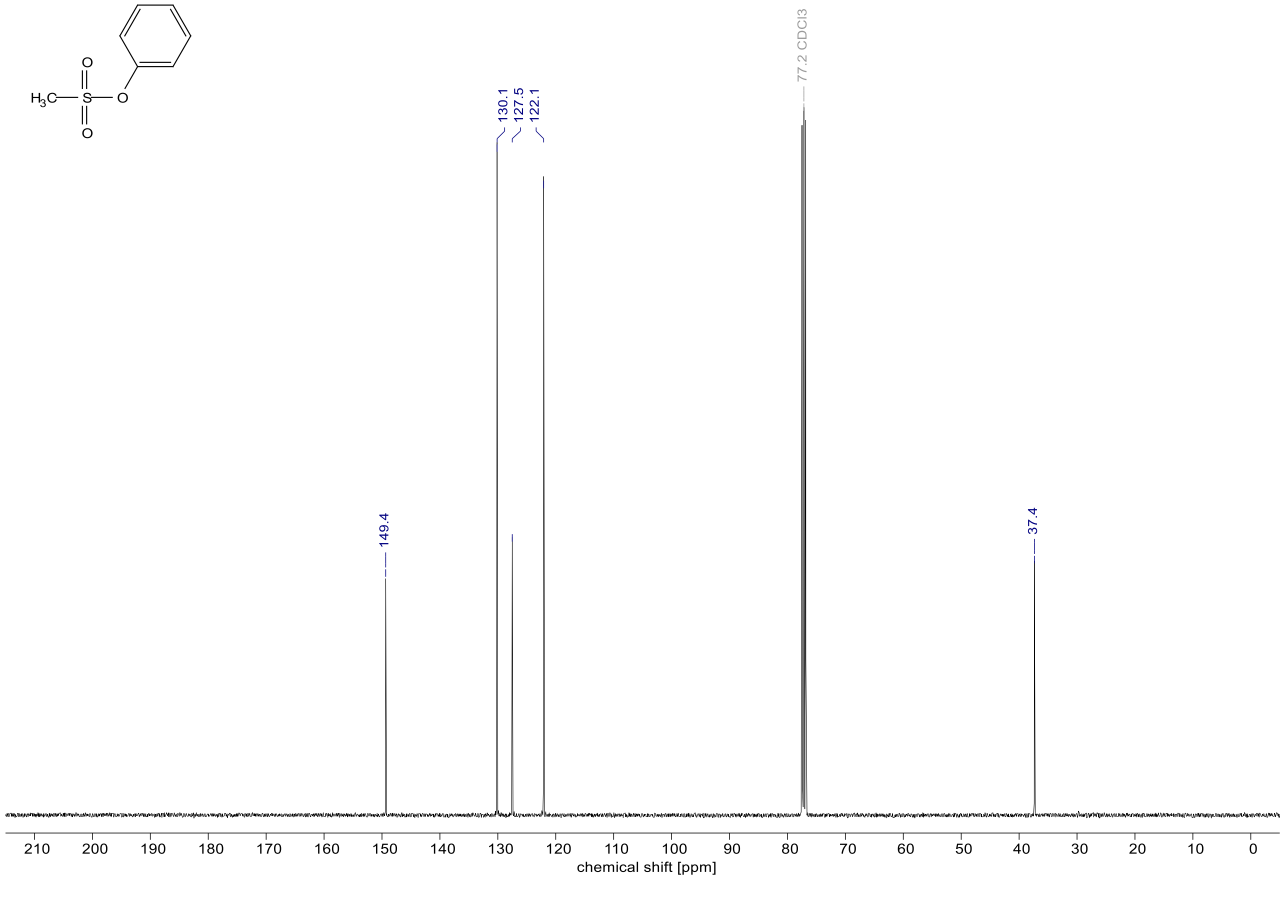

Figure S24: $^{13}C\{^{1}H\}$ NMR spectrum (101 MHz, 25 °C, $CDCl_3$) of **3a**.

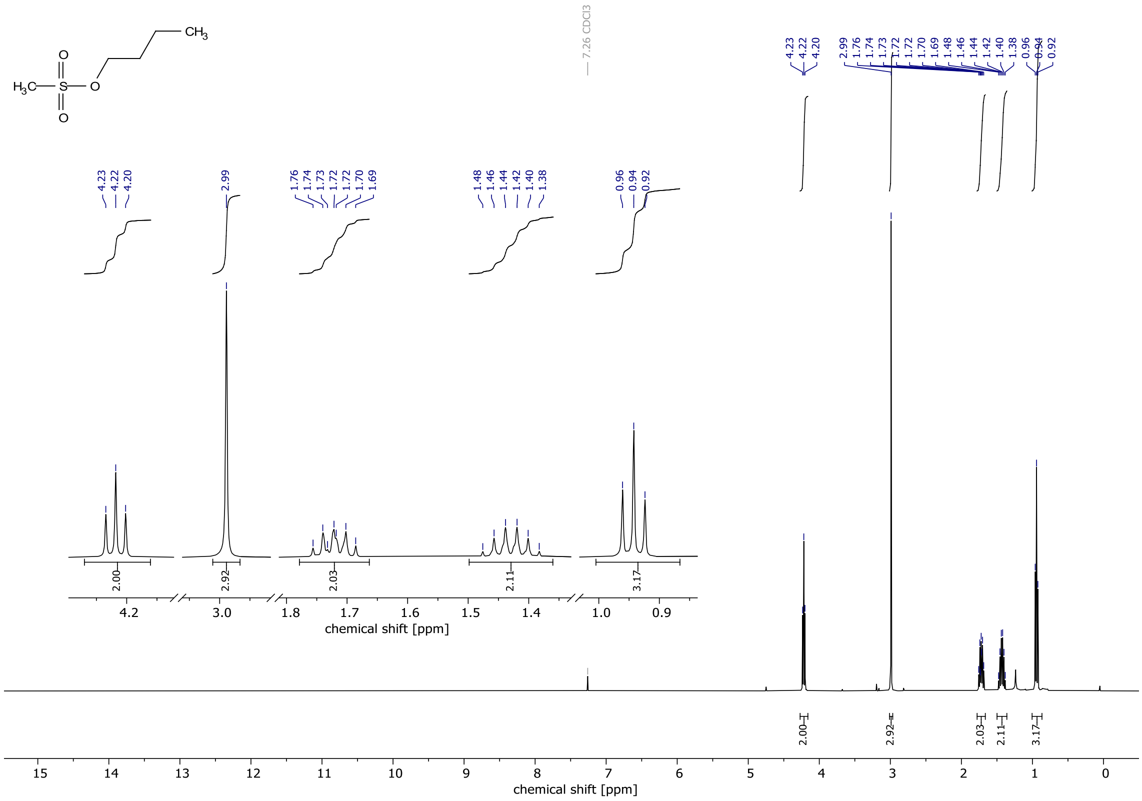

Figure S25: ^{1}H NMR spectrum (400 MHz, 25 °C, $CDCl_3$) of **3b**.

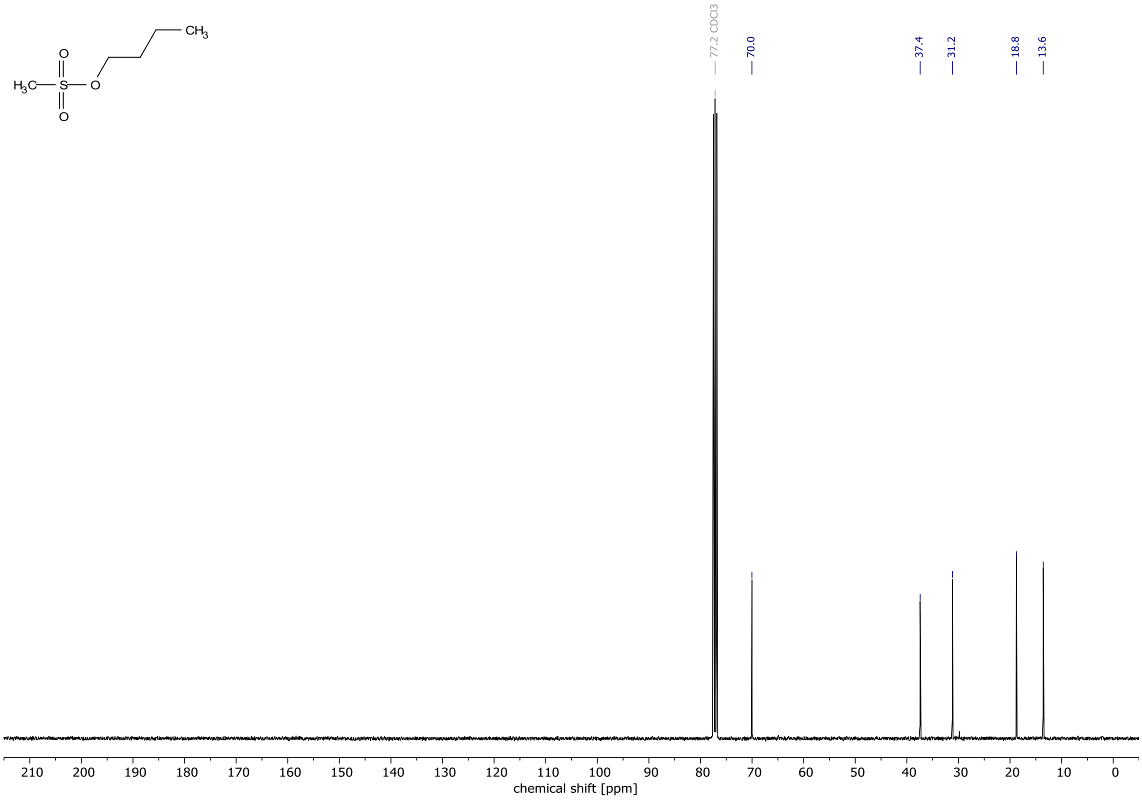

Figure S26: $^{13}C\{^{1}H\}$ NMR spectrum (101 MHz, 25 °C, $CDCl_3$) of **3b**.

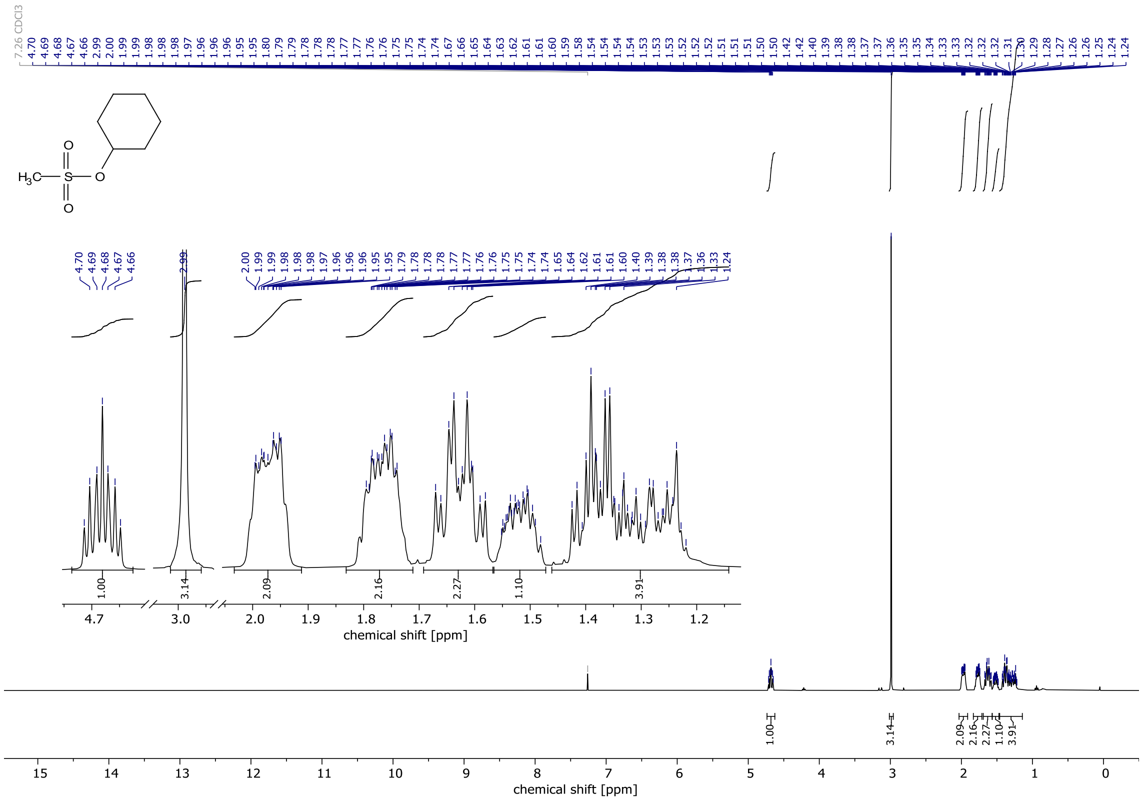

Figure S27: ^{1}H NMR spectrum (400 MHz, 25 °C, $CDCl_3$) of **3c**.

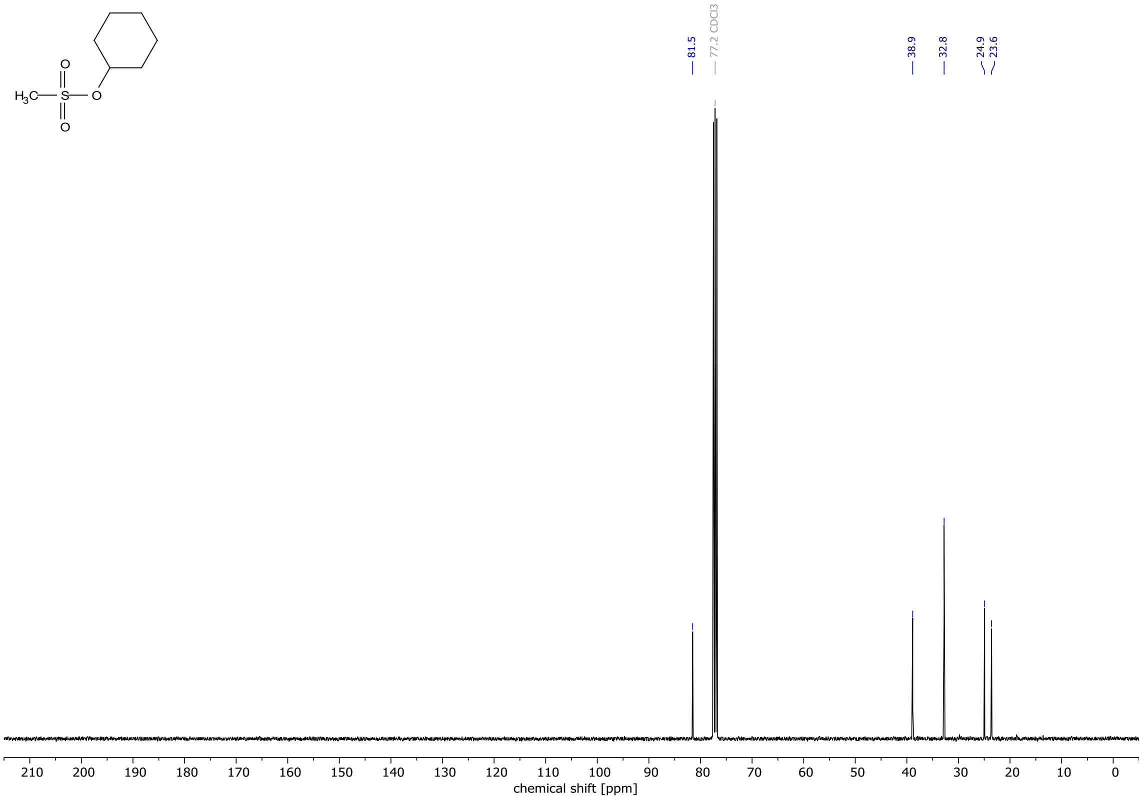

Figure S28: $^{13}C\{^1H\}$ NMR spectrum (101 MHz, 25 °C, $CDCl_3$) of **3c**.

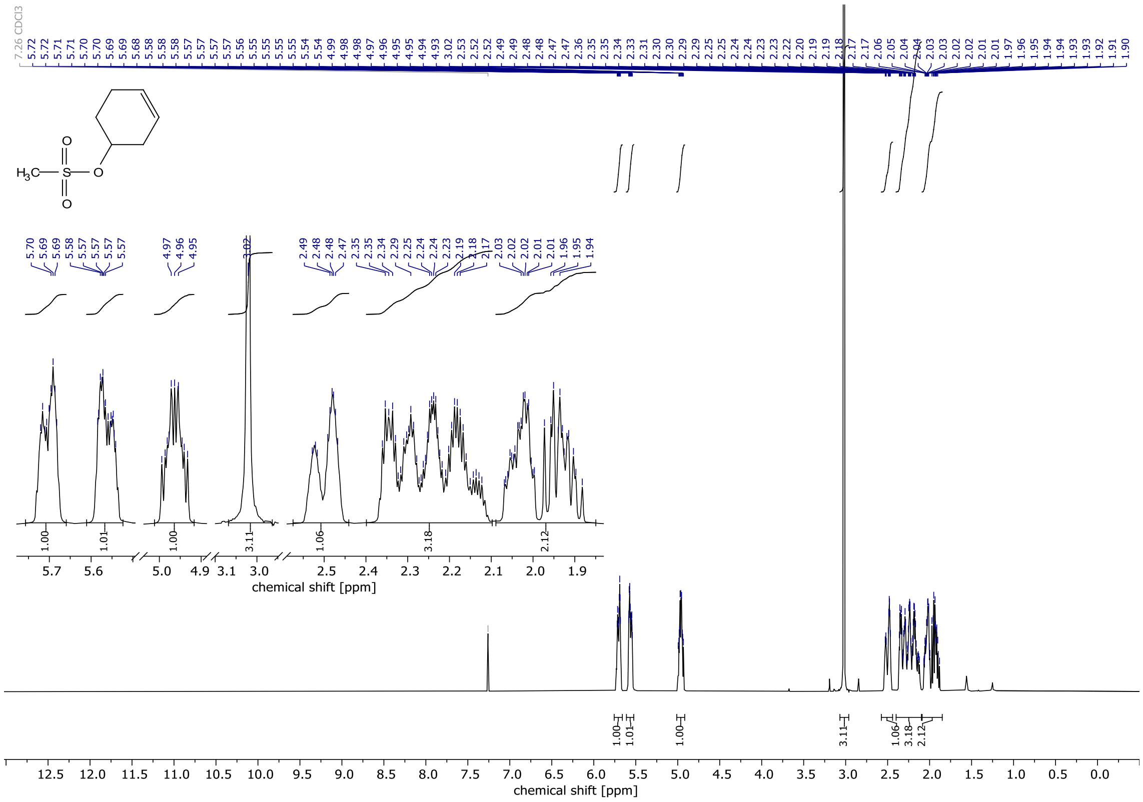

Figure S29: ^{1}H NMR spectrum (400 MHz, 25 °C, $CDCl_3$) of **3d**.

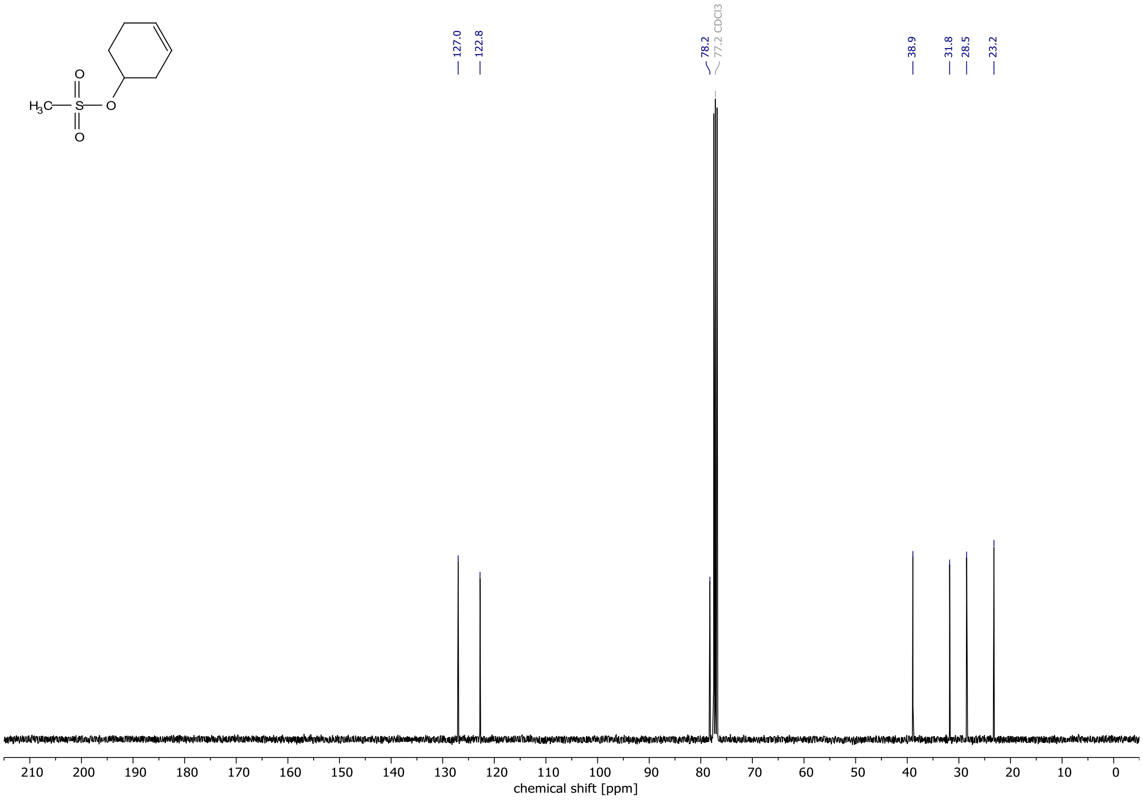

Figure S30: $^{13}C\{^{1}H\}$ NMR spectrum (101 MHz, 25 °C, $CDCl_3$) of **3d**.

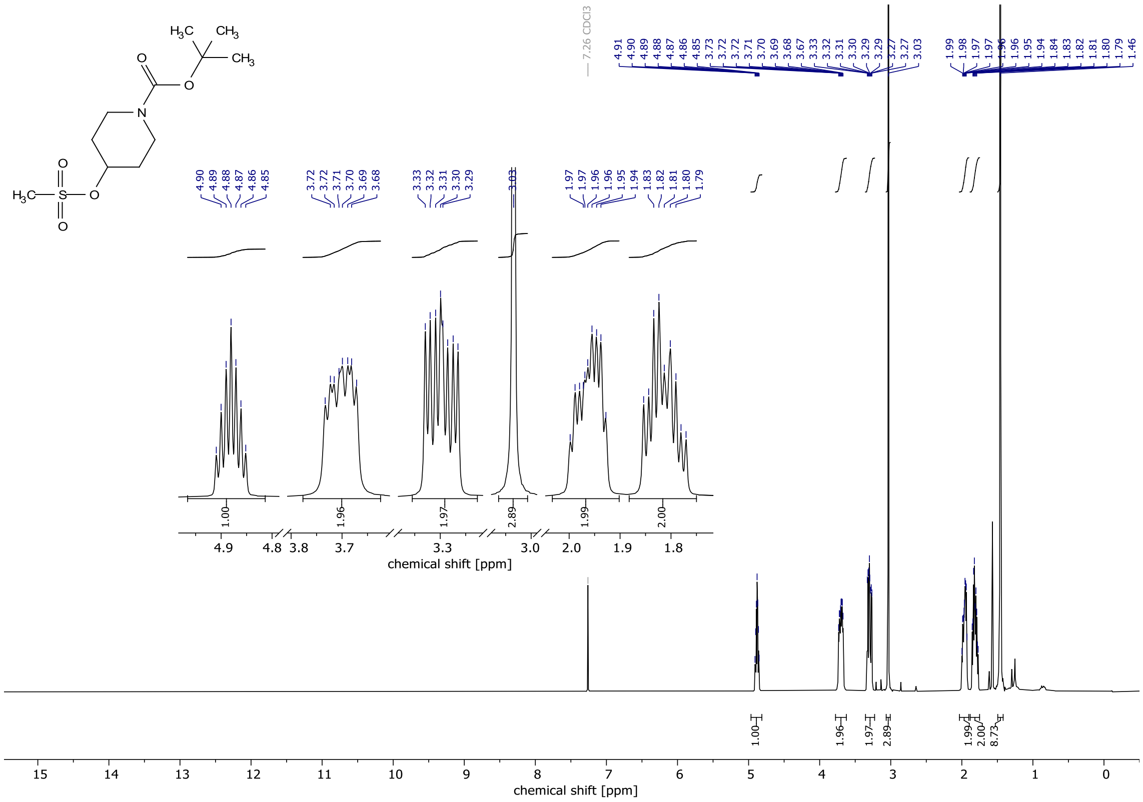

Figure S31: ^{1}H NMR spectrum (400 MHz, 25 °C, $CDCl_3$) of **3e**.

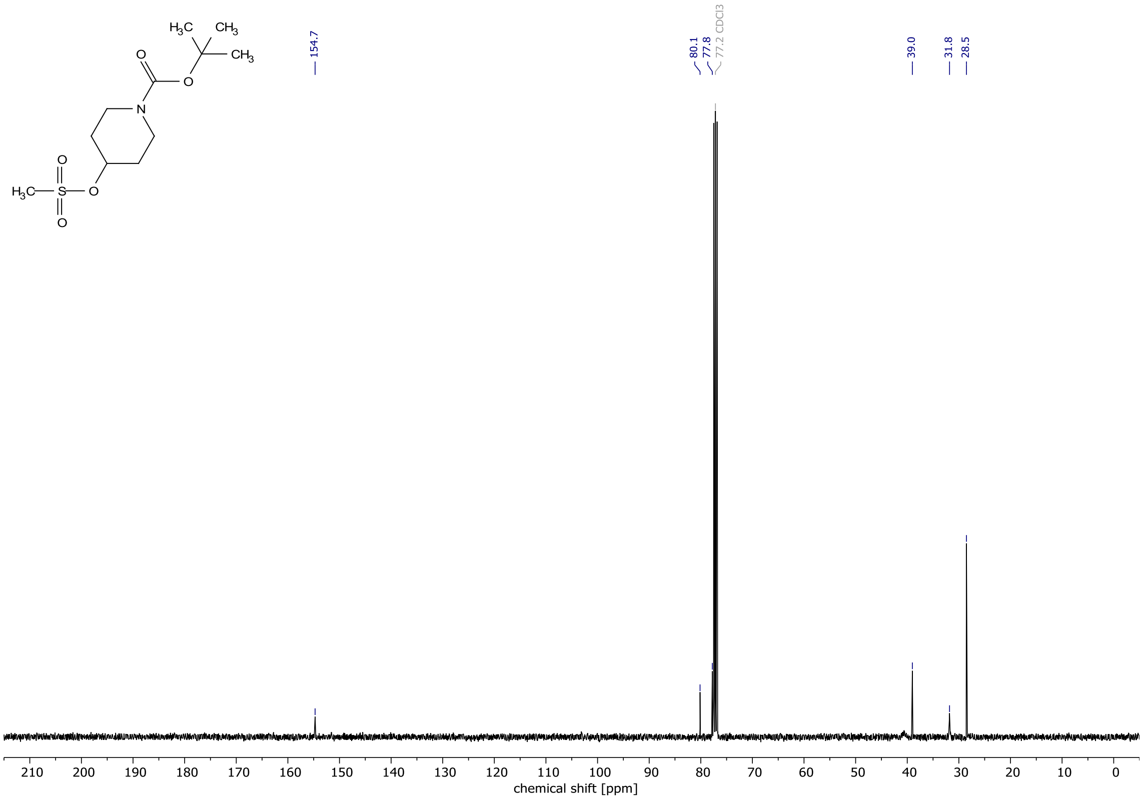

Figure S32: $^{13}C\{^{1}H\}$ NMR spectrum (101 MHz, 25 °C, $CDCl_3$) of **3e**.

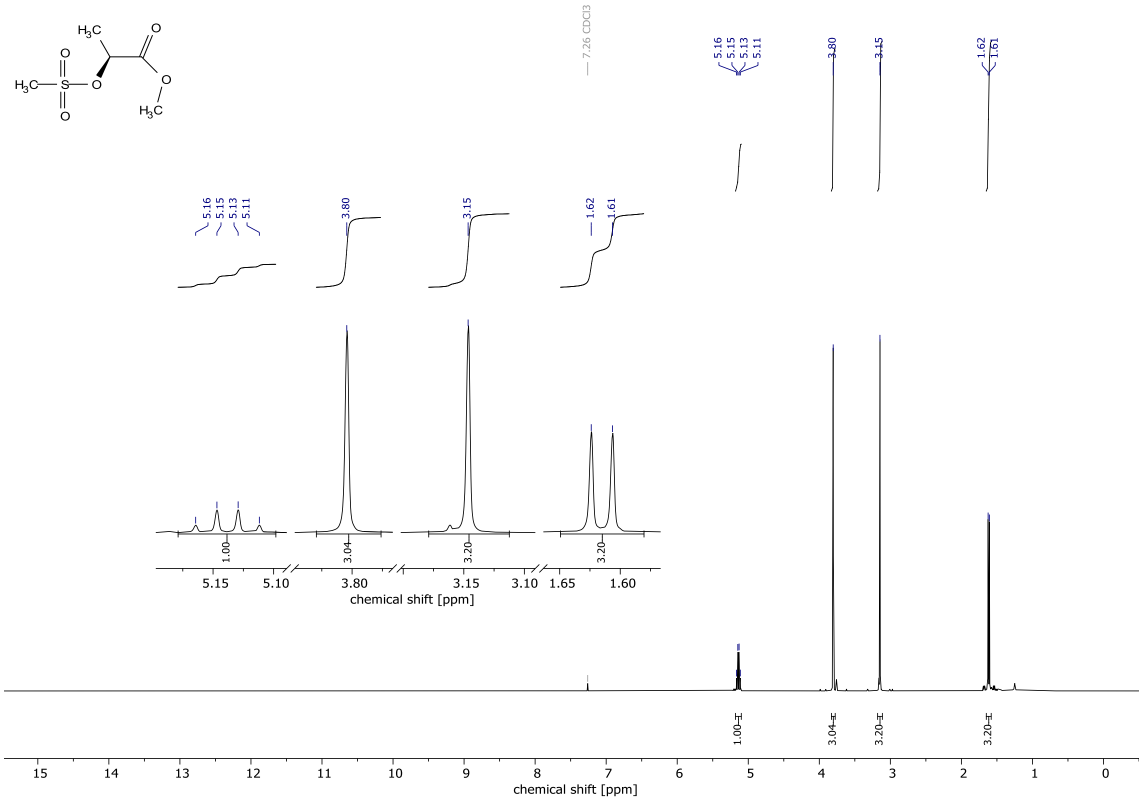

Figure S33: ^{1}H NMR spectrum (400 MHz, 25 °C, $CDCl_3$) of **3f**.

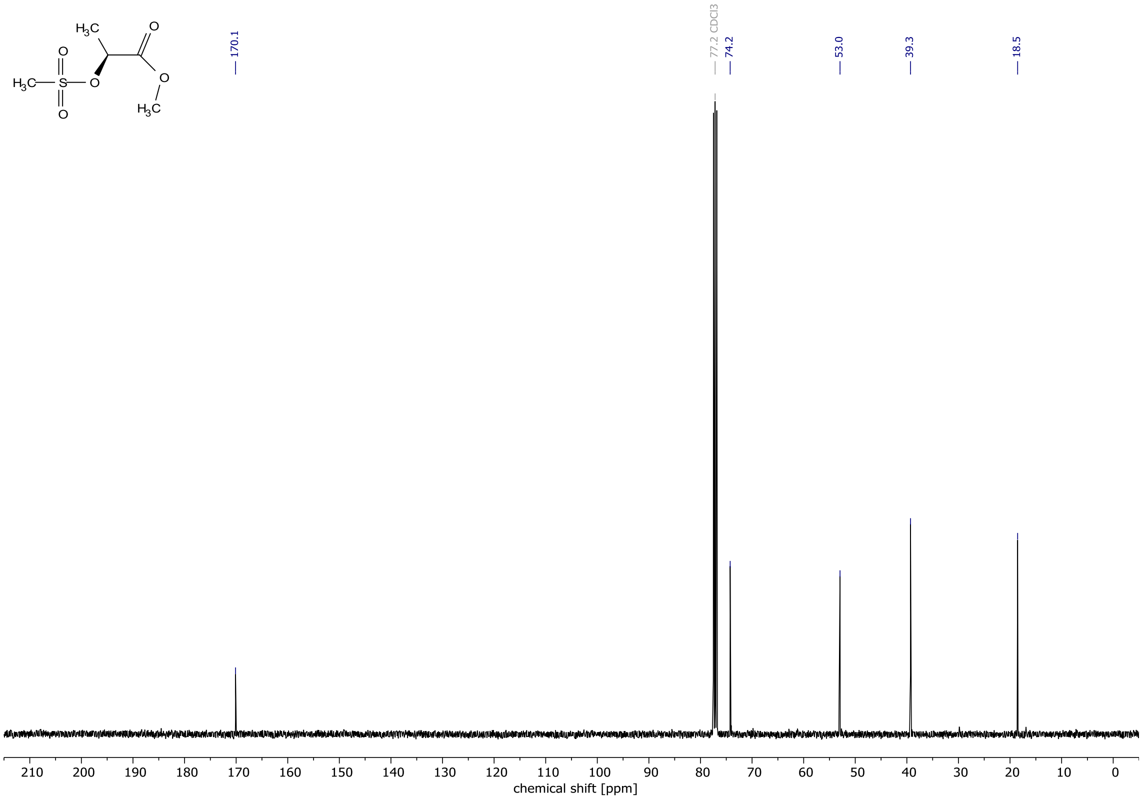

Figure S34: $^{13}C\{^{1}H\}$ NMR spectrum (101 MHz, 25 °C, $CDCl_3$) of **3f**.

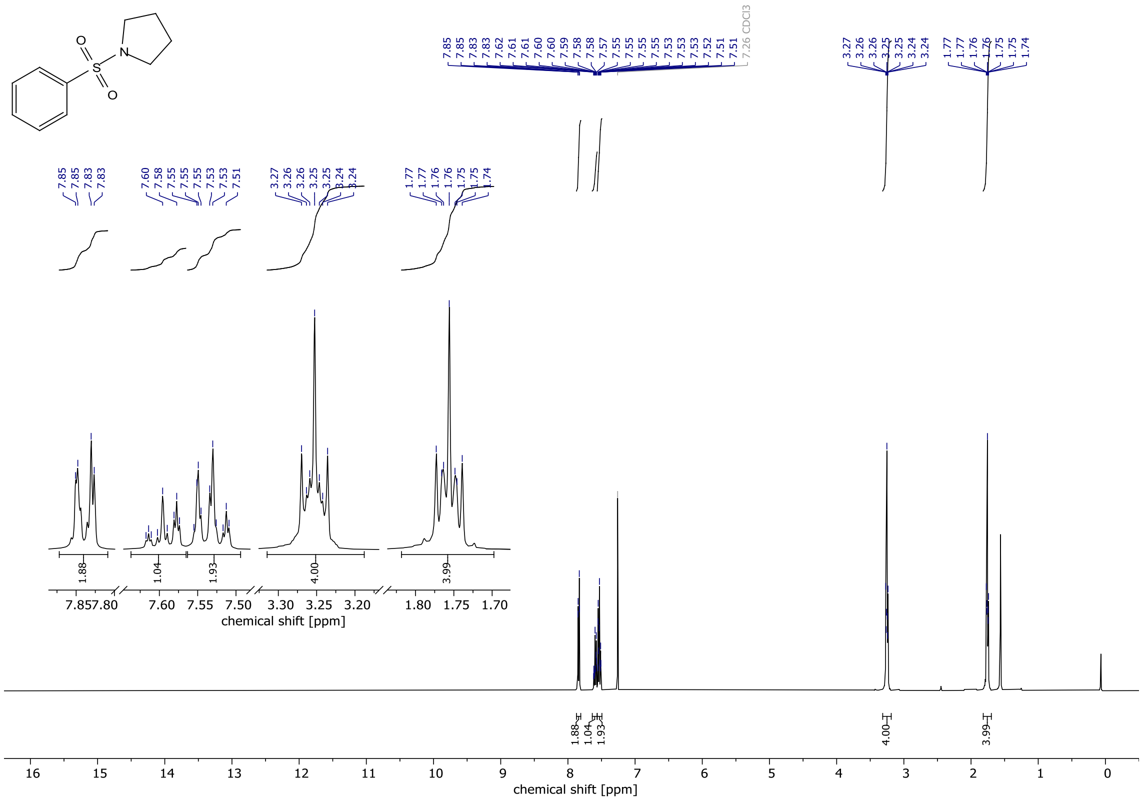

Figure S35: ^{1}H NMR spectrum (400 MHz, 25 °C, $CDCl_3$) of **4a**.

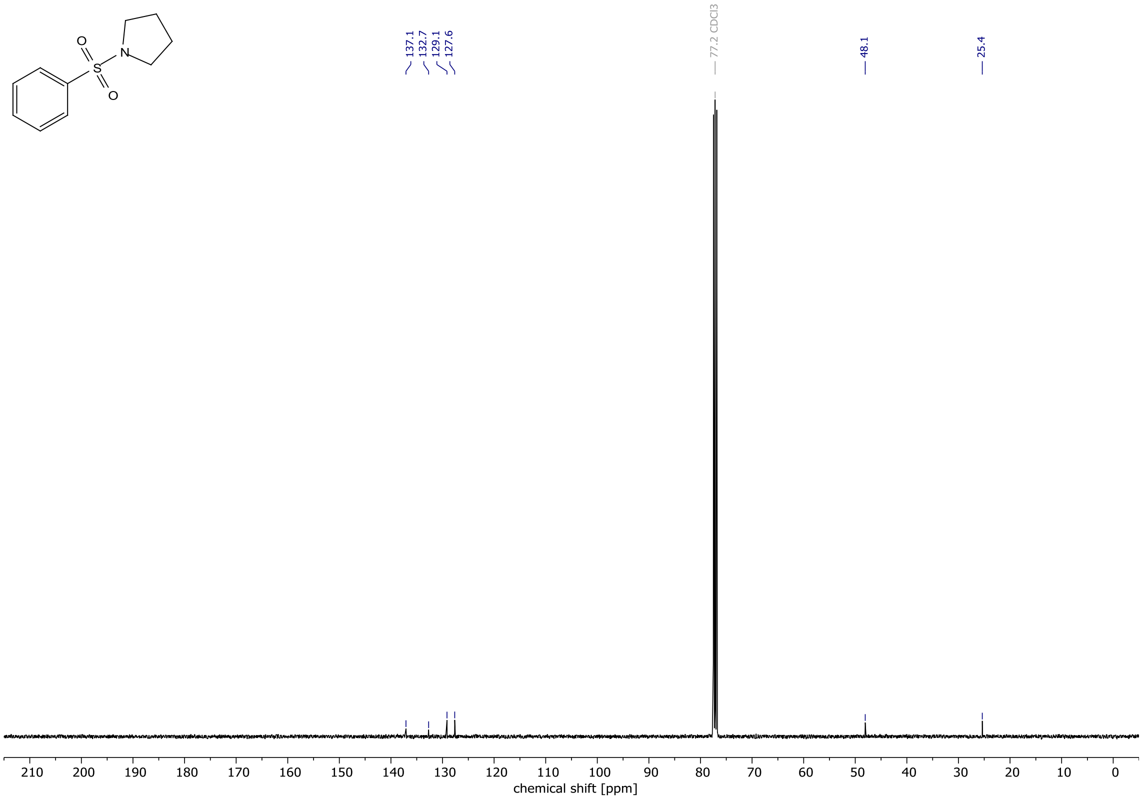

Figure S36: $^{13}C\{^{1}H\}$ NMR spectrum (101 MHz, 25 °C, $CDCl_3$) of **4a**.

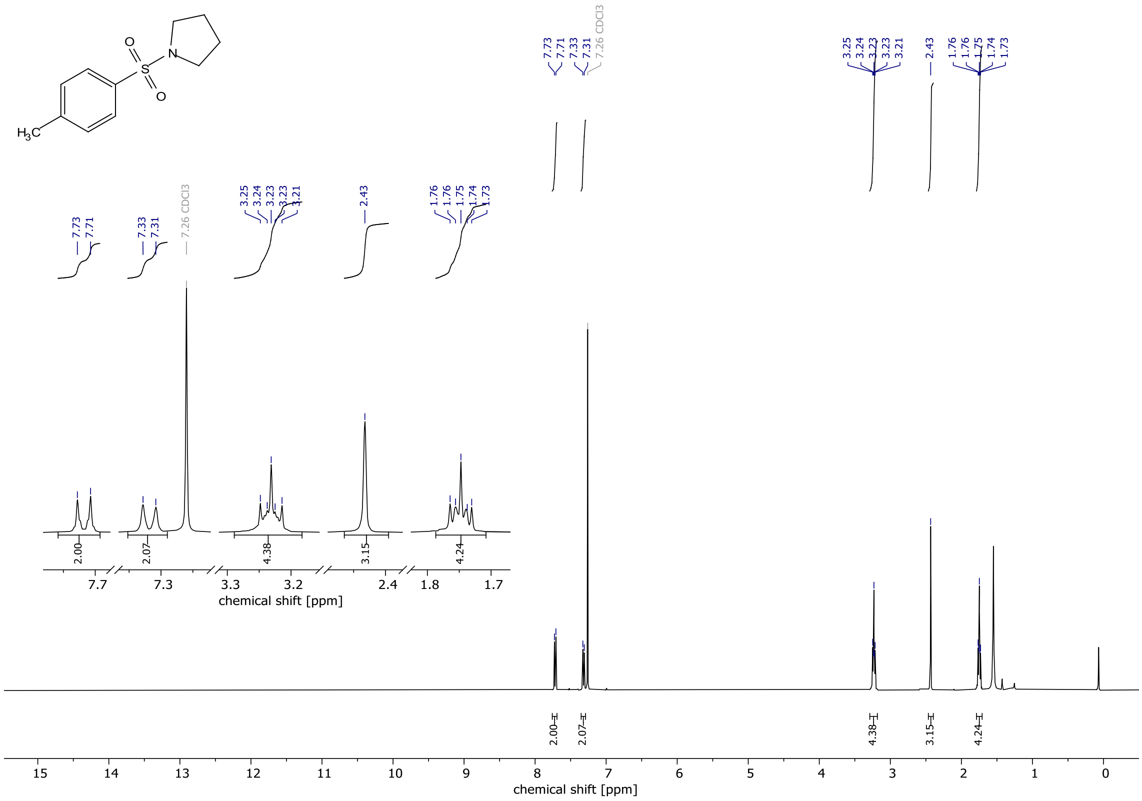

Figure S37: ^{1}H NMR spectrum (400 MHz, 25 °C, $CDCl_3$) of **4b**.

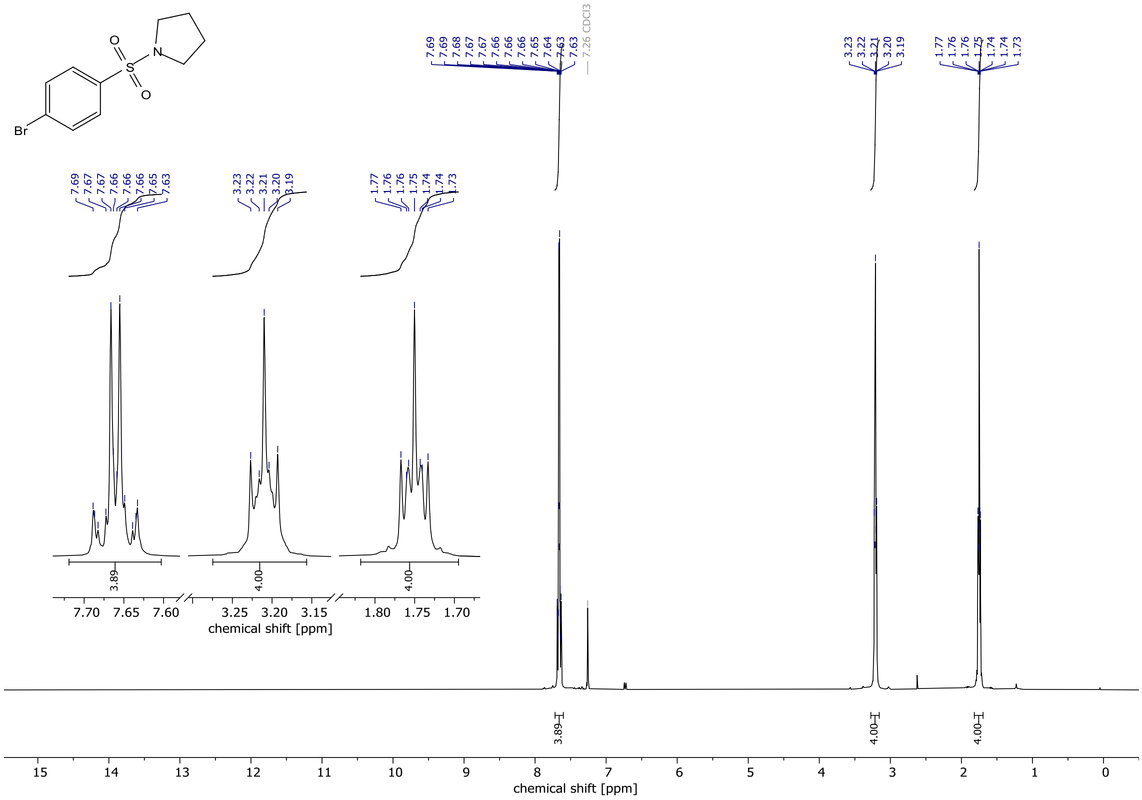

Figure S38: ^{1}H NMR spectrum (400 MHz, 25 °C, $CDCl_3$) of **4c**.

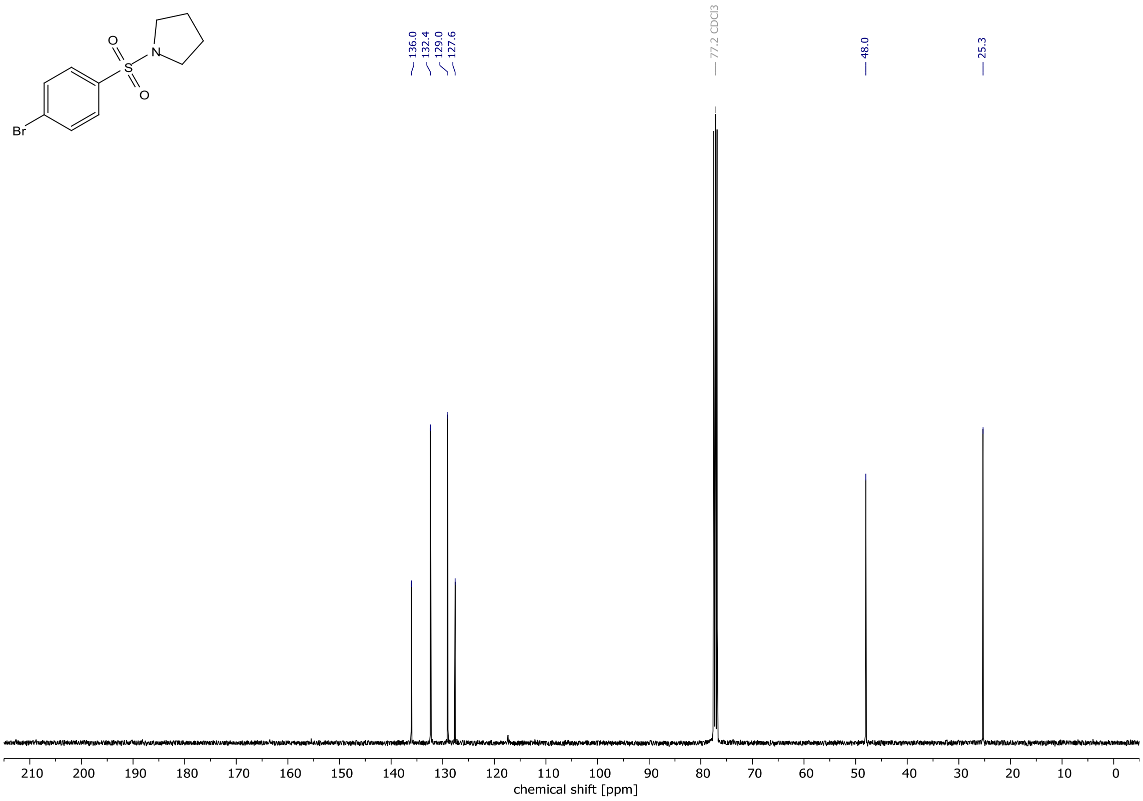

Figure S39: $^{13}C\{^{1}H\}$ NMR spectrum (101 MHz, 25 °C, $CDCl_3$) of **4c**.

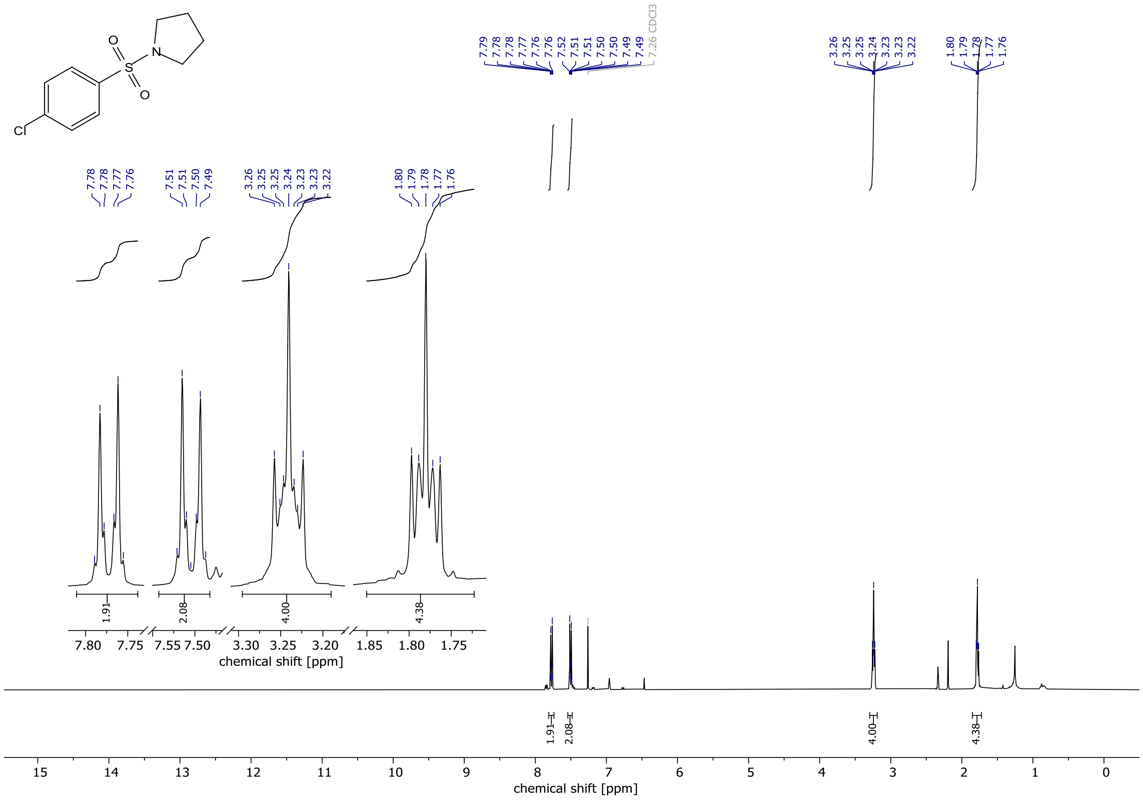

Figure S40: ^{1}H NMR spectrum (400 MHz, 25 °C, $CDCl_3$) of **4d**.

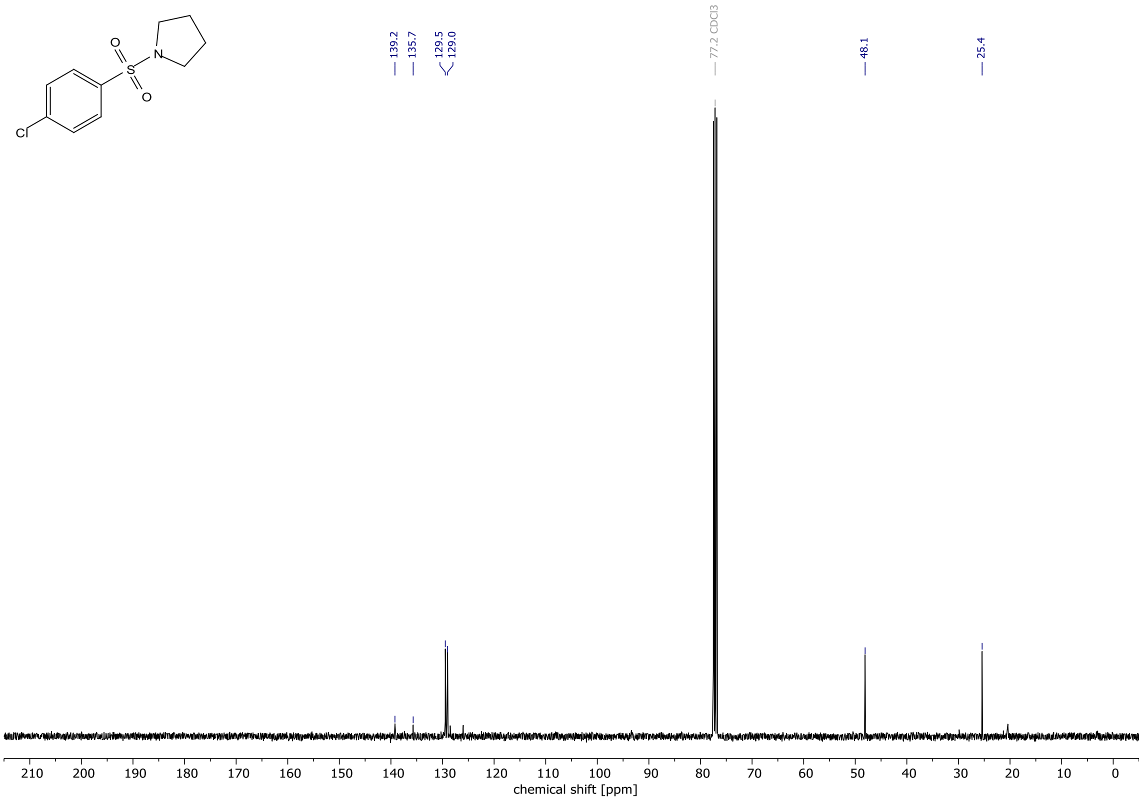

Figure S41: $^{13}C\{^{1}H\}$ NMR spectrum (101 MHz, 25 °C, $CDCl_3$) of **4d**.

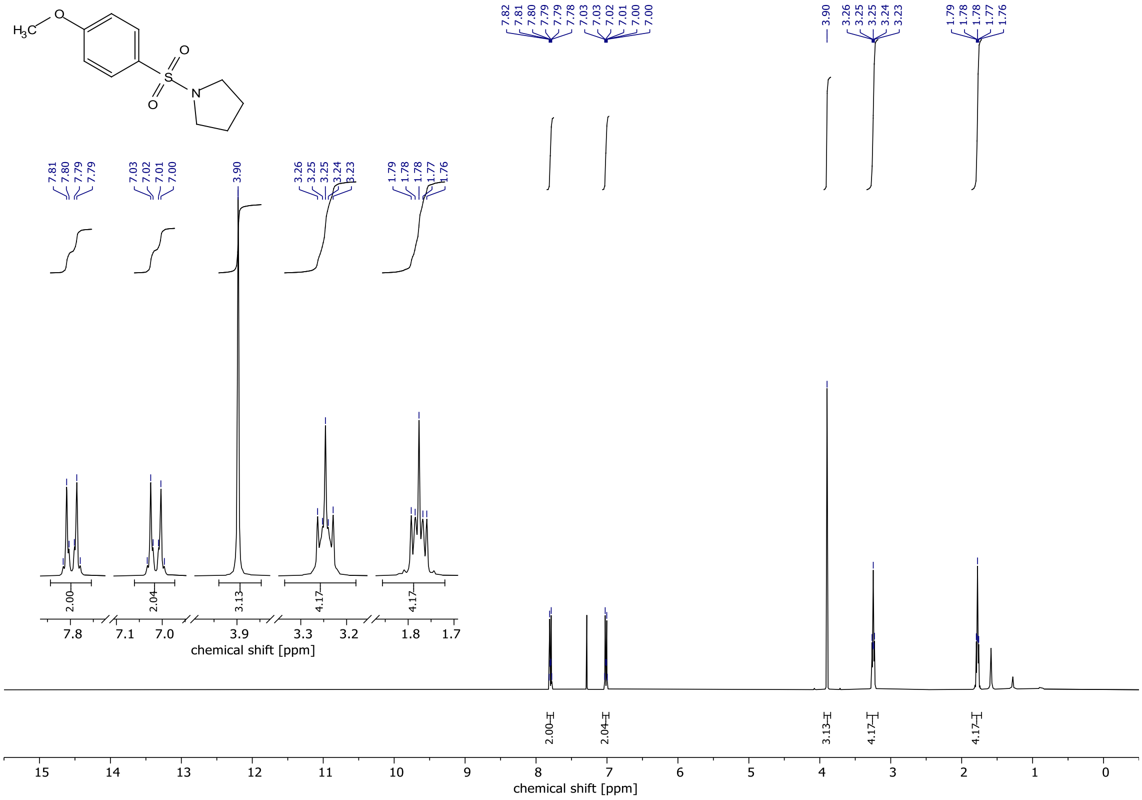

Figure S42: ^{1}H NMR spectrum (400 MHz, 25 °C, $CDCl_3$) of **4e**.

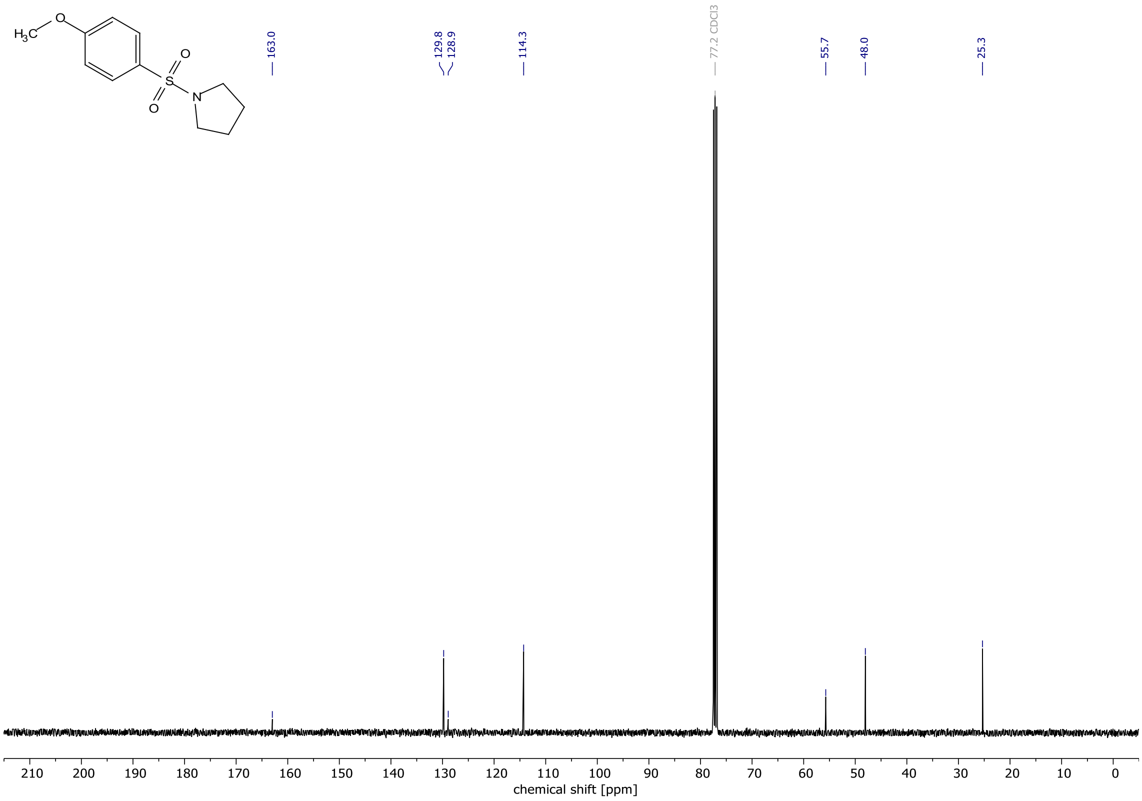

Figure S43: $^{13}C\{^1H\}$ NMR spectrum (101 MHz, 25 °C, $CDCl_3$) of **4e**.

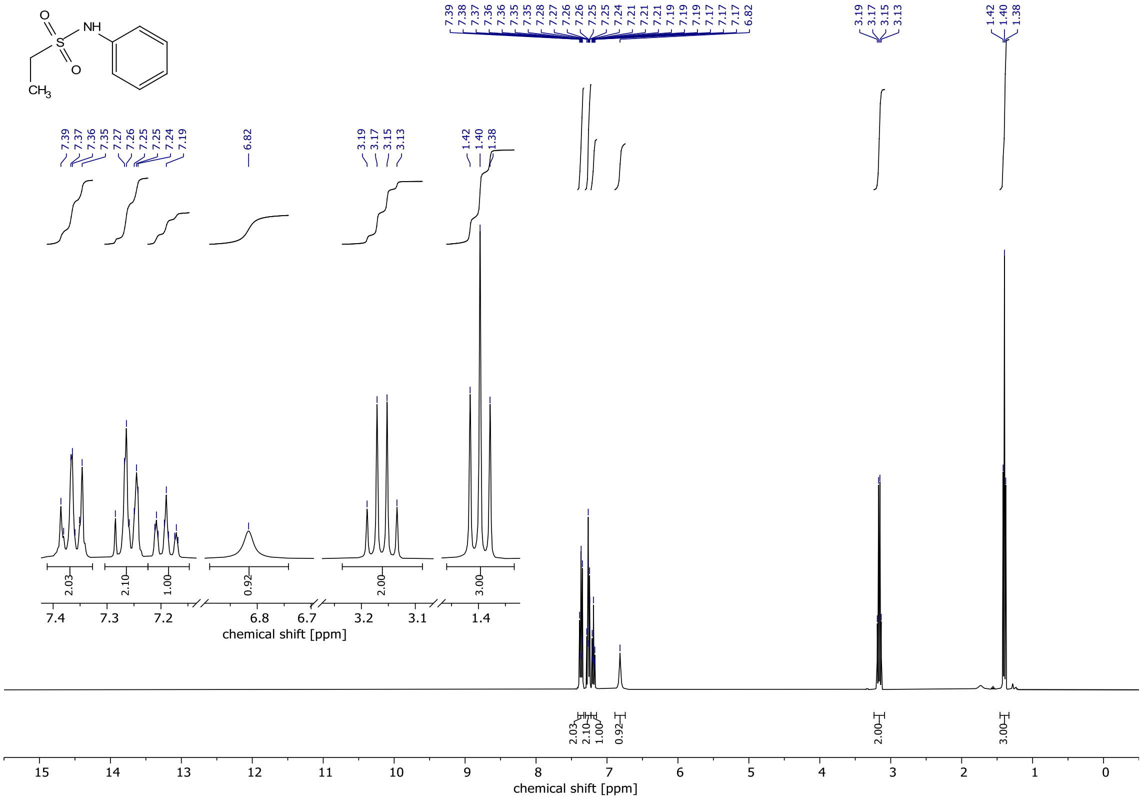

Figure S44: ^1H NMR spectrum (400 MHz, 25 °C, $CDCl_3$) of **4f**.

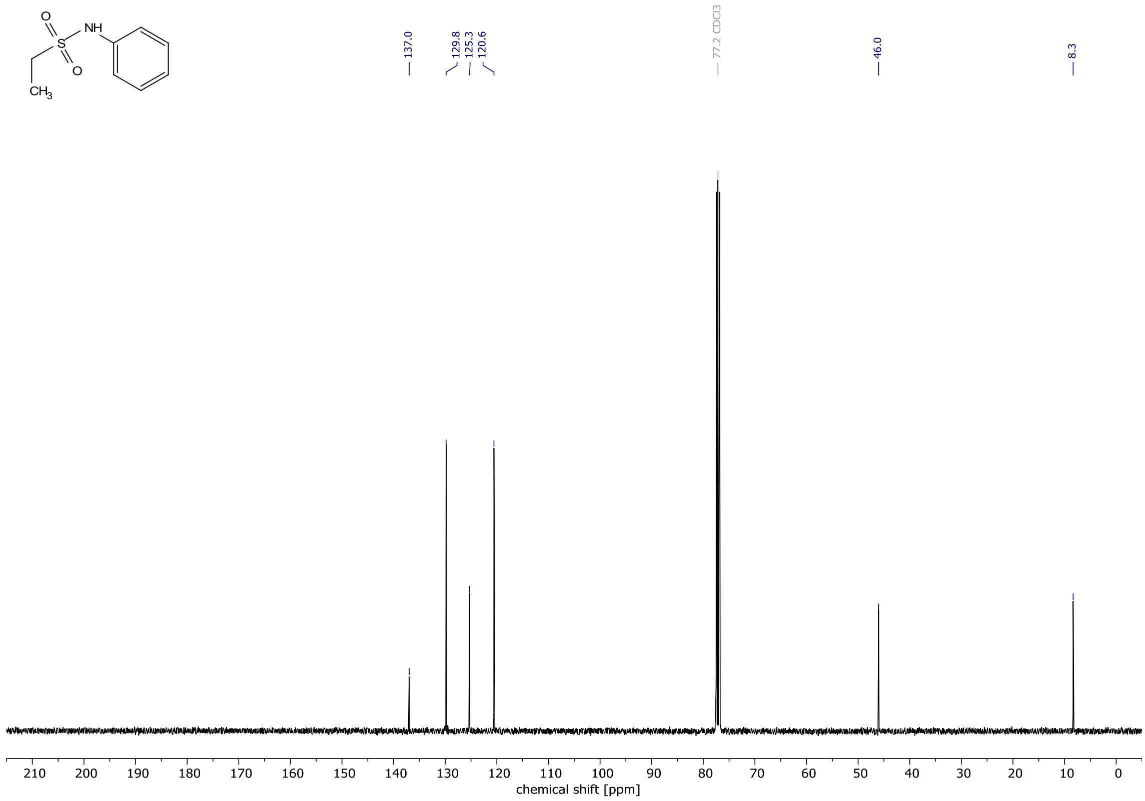

Figure S45: $^{13}C\{^{1}H\}$ NMR spectrum (101 MHz, 25 °C, $CDCl_3$) of **4f**.

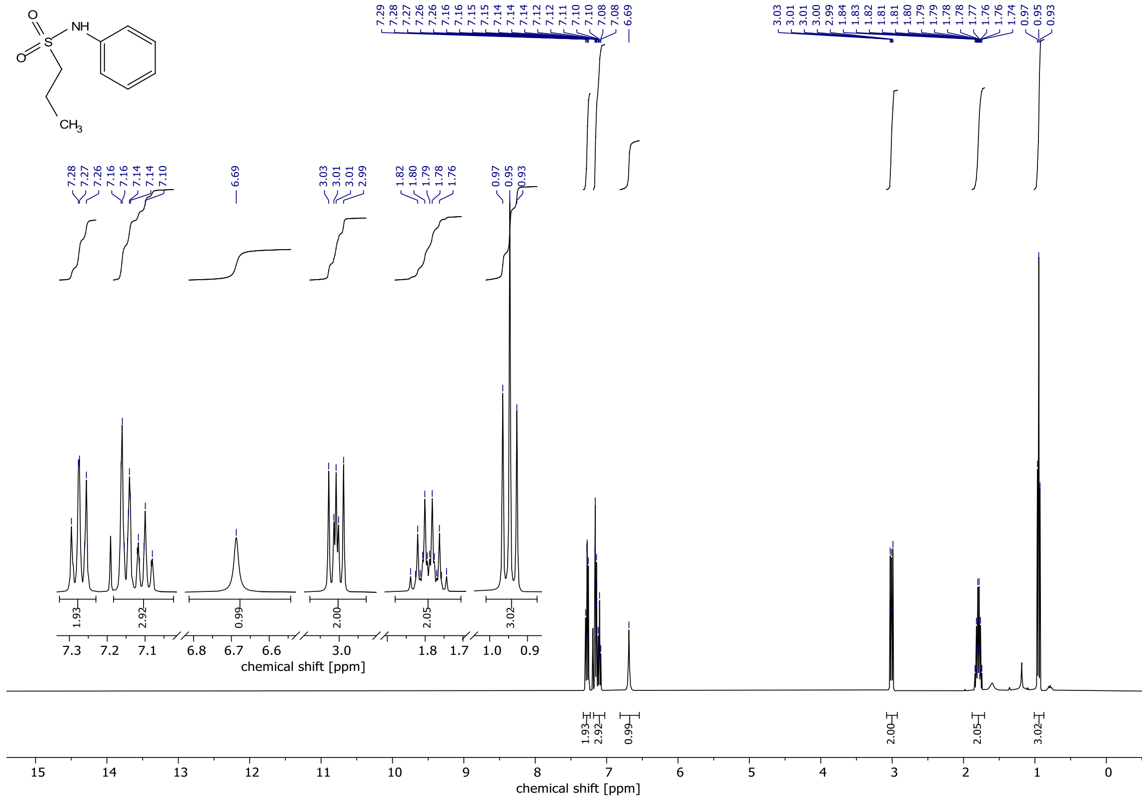

Figure S46: ^{1}H NMR spectrum (400 MHz, 25 °C, $CDCl_3$) of **4g**.

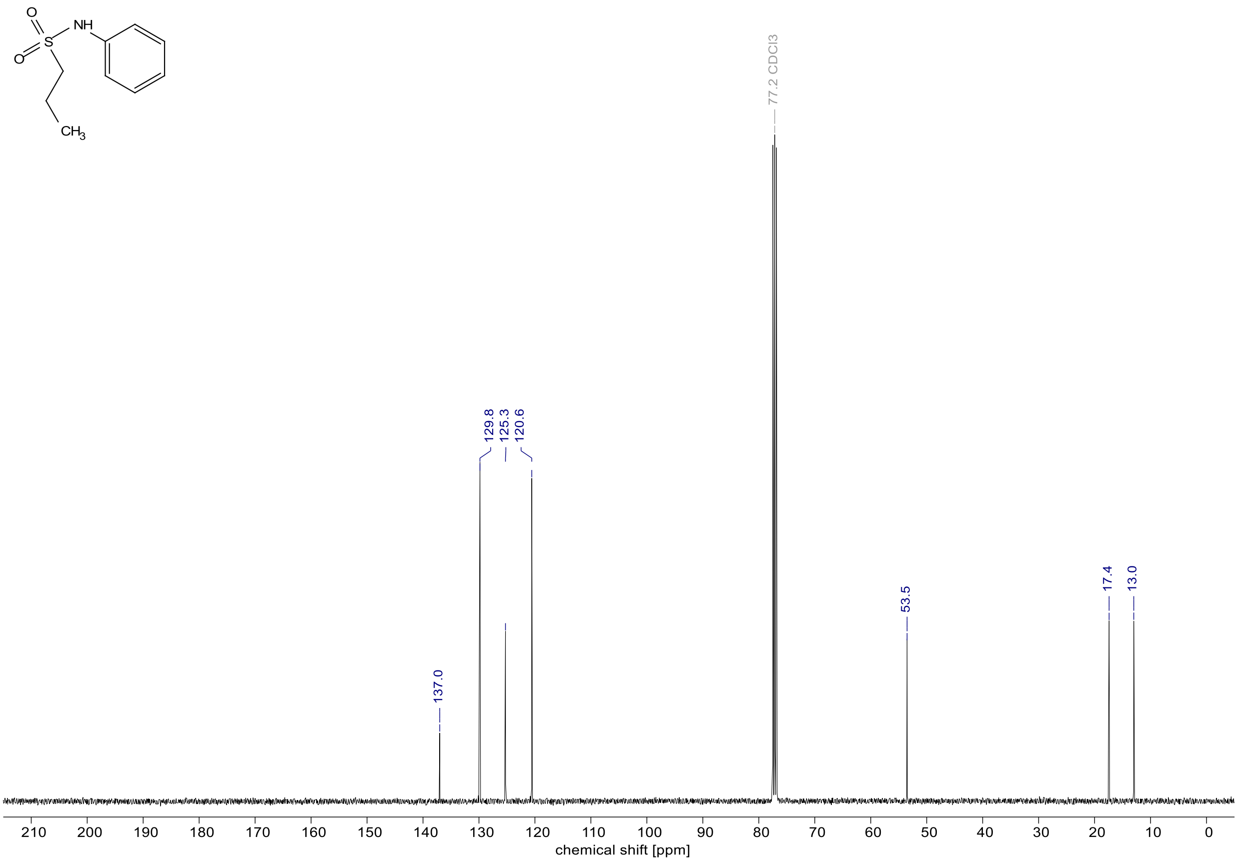

Figure S47: $^{13}C\{^1H\}$ NMR spectrum (101 MHz, 25 °C, $CDCl_3$) of **4g**.

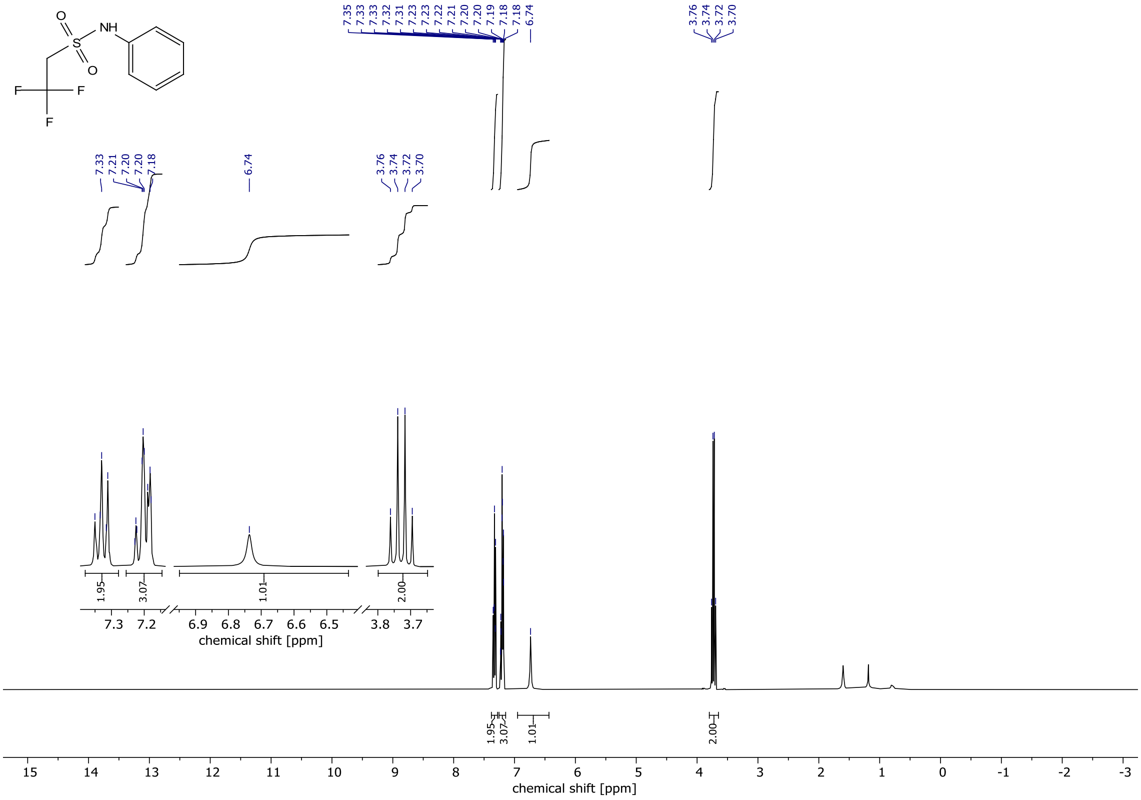

Figure S48: ^{1}H NMR spectrum (400 MHz, 25 °C, $CDCl_3$) of **4h**.

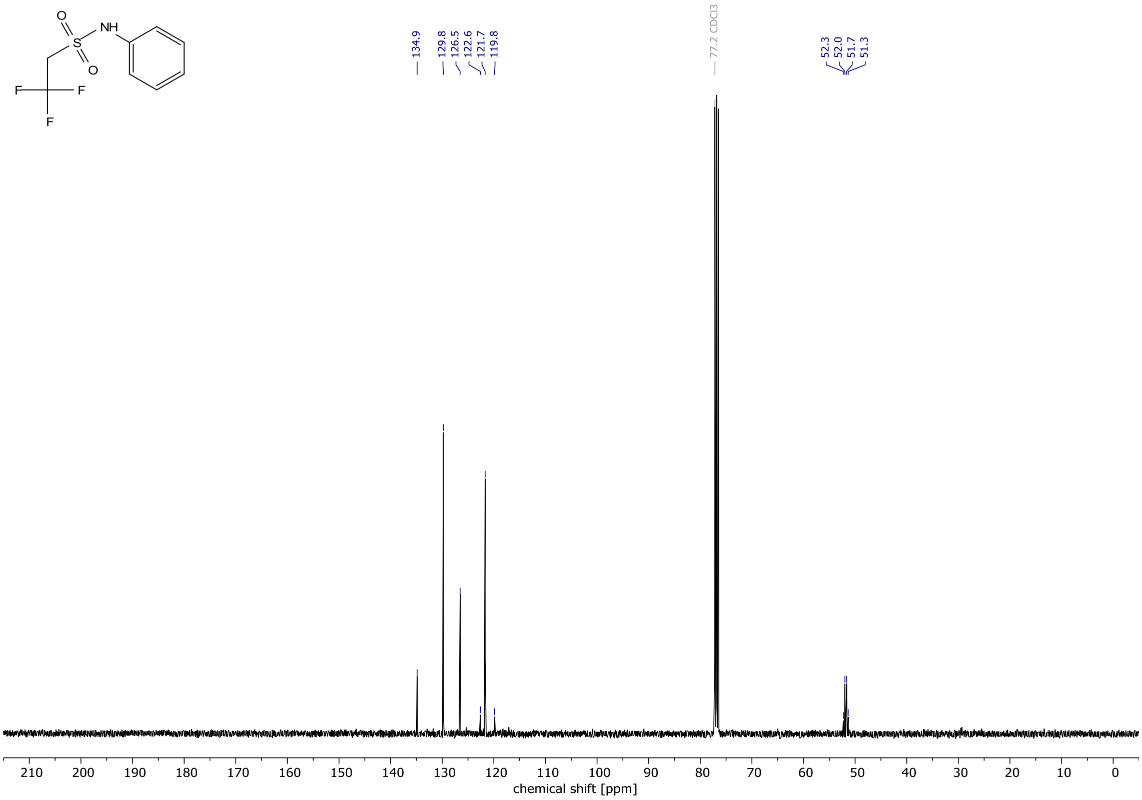

Figure S49: $^{13}C\{^{1}H\}$ NMR spectrum (101 MHz, 25 °C, $CDCl_3$) of **4h**.

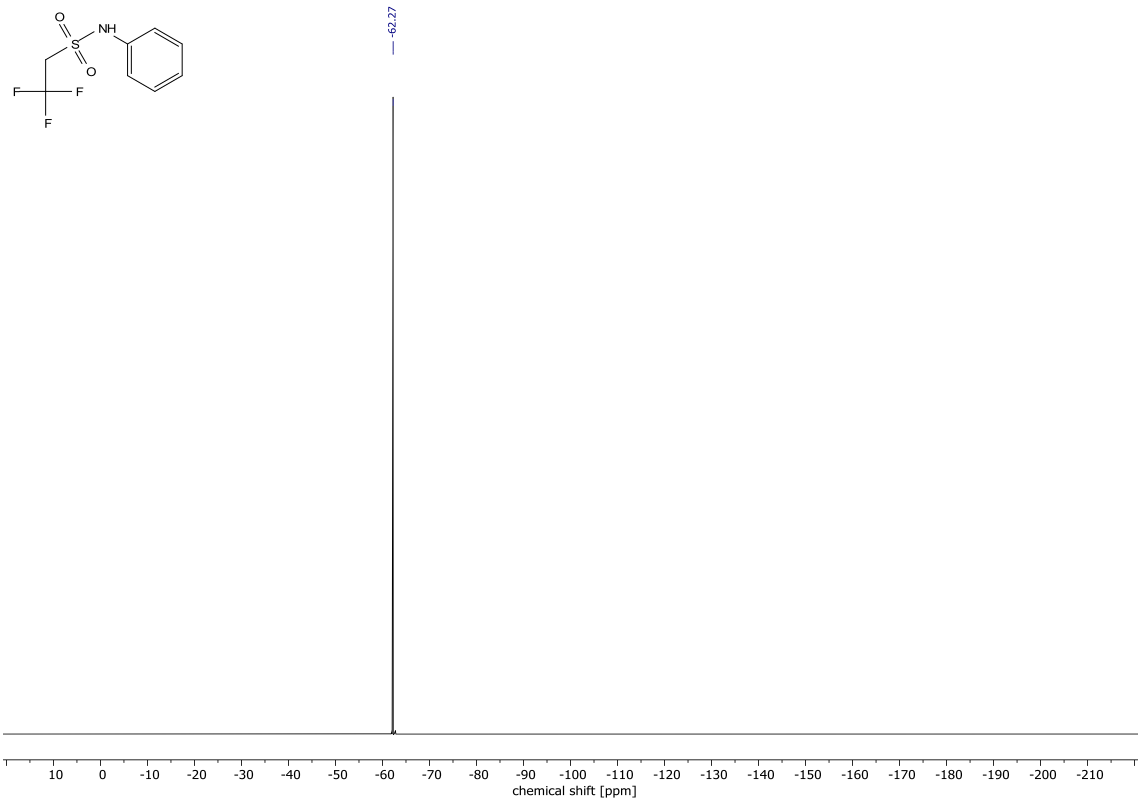

Figure S50: ^{19}F NMR (376 MHz, 25 °C, $CDCl_3$) of **4h**.

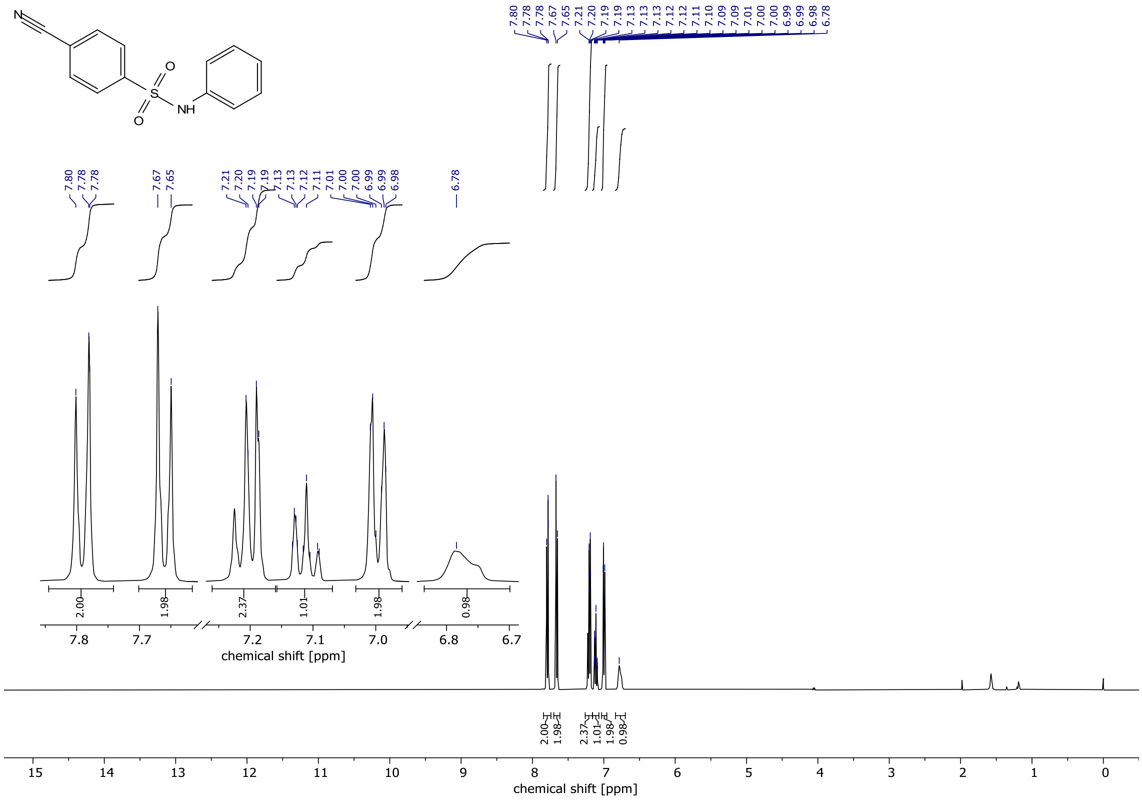

Figure S51: ^{1}H NMR spectrum (400 MHz, 25 °C, $CDCl_3$) of **4i**.

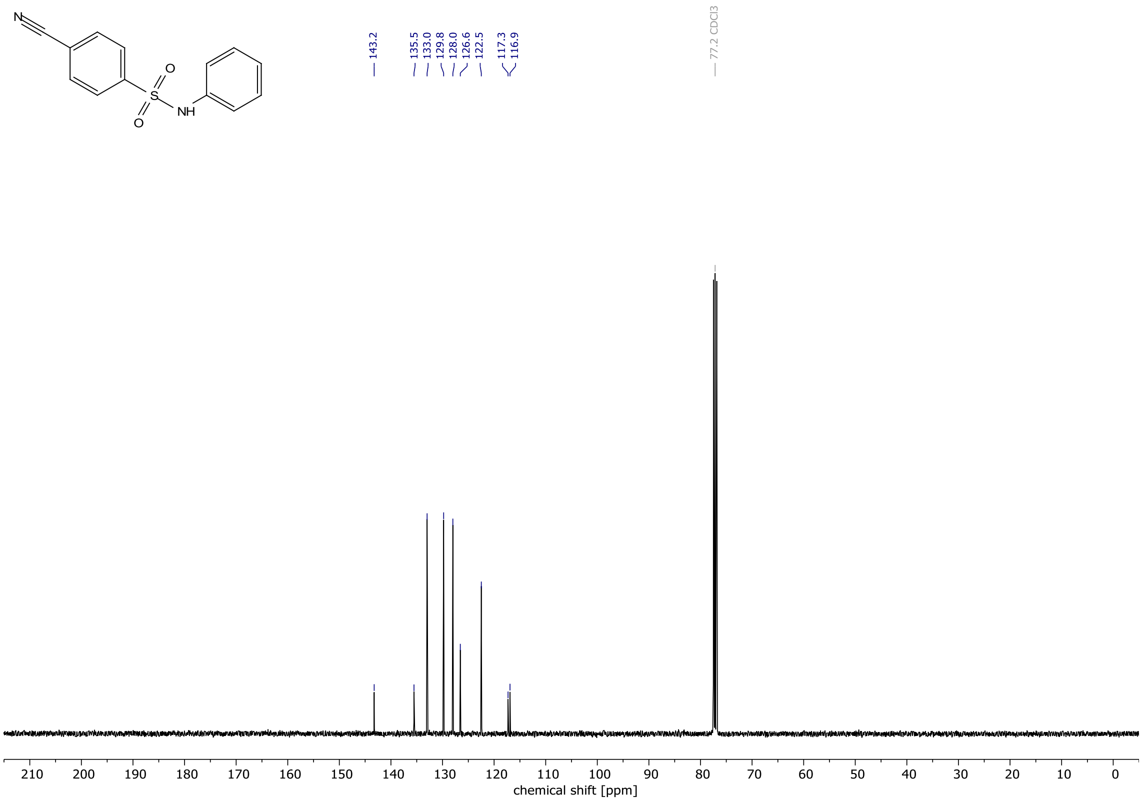

Figure S52: $^{13}C\{^{1}H\}$ NMR spectrum (101 MHz, 25 °C, $CDCl_3$) of **4i**.

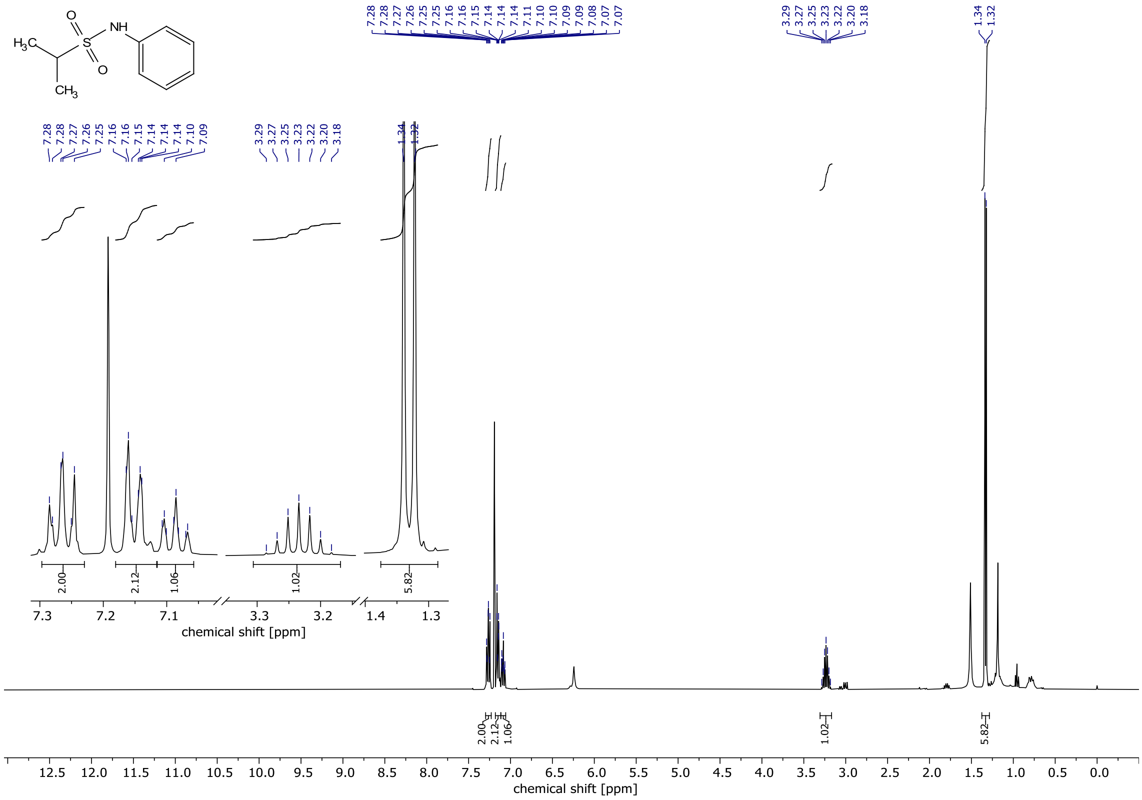

Figure S53: ^{1}H NMR spectrum (400 MHz, 25 °C, $CDCl_3$) of **4j**.

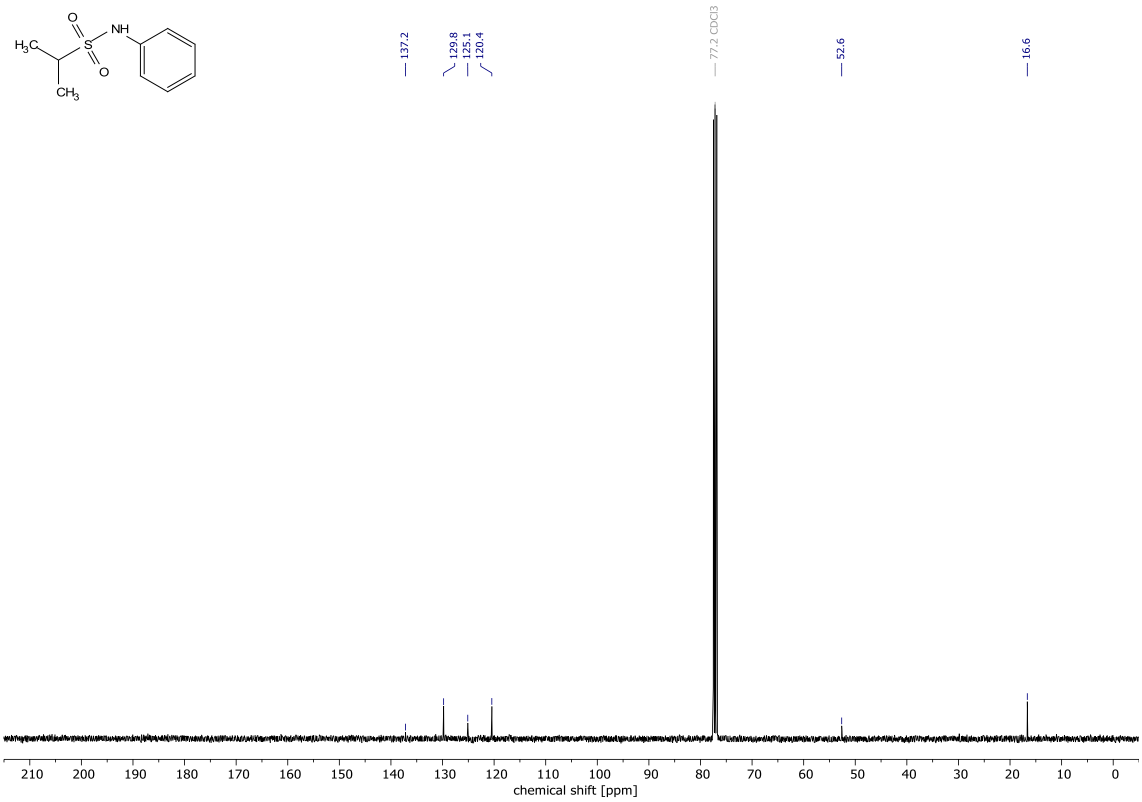

Figure S54: $^{13}C\{^{1}H\}$ NMR spectrum (101 MHz, 25 °C, $CDCl_3$) of **4j**.

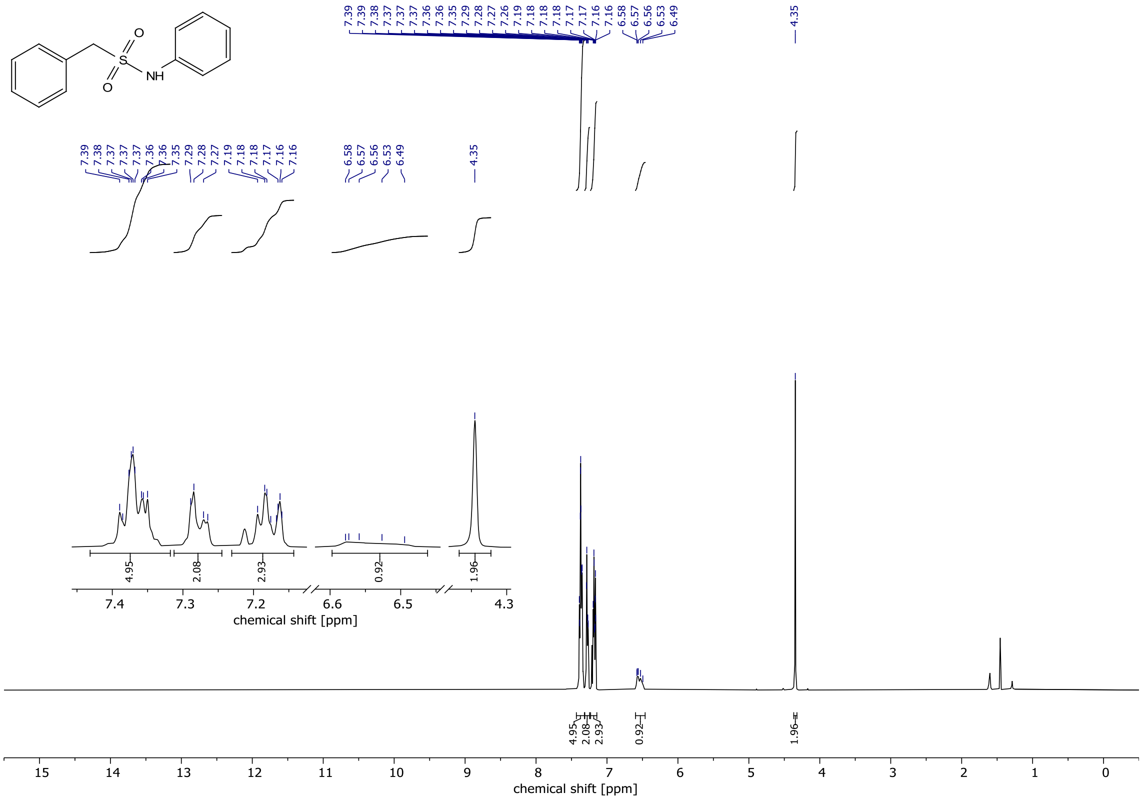

Figure S55: ^{1}H NMR spectrum (400 MHz, 25 °C, $CDCl_3$) of **4k**.

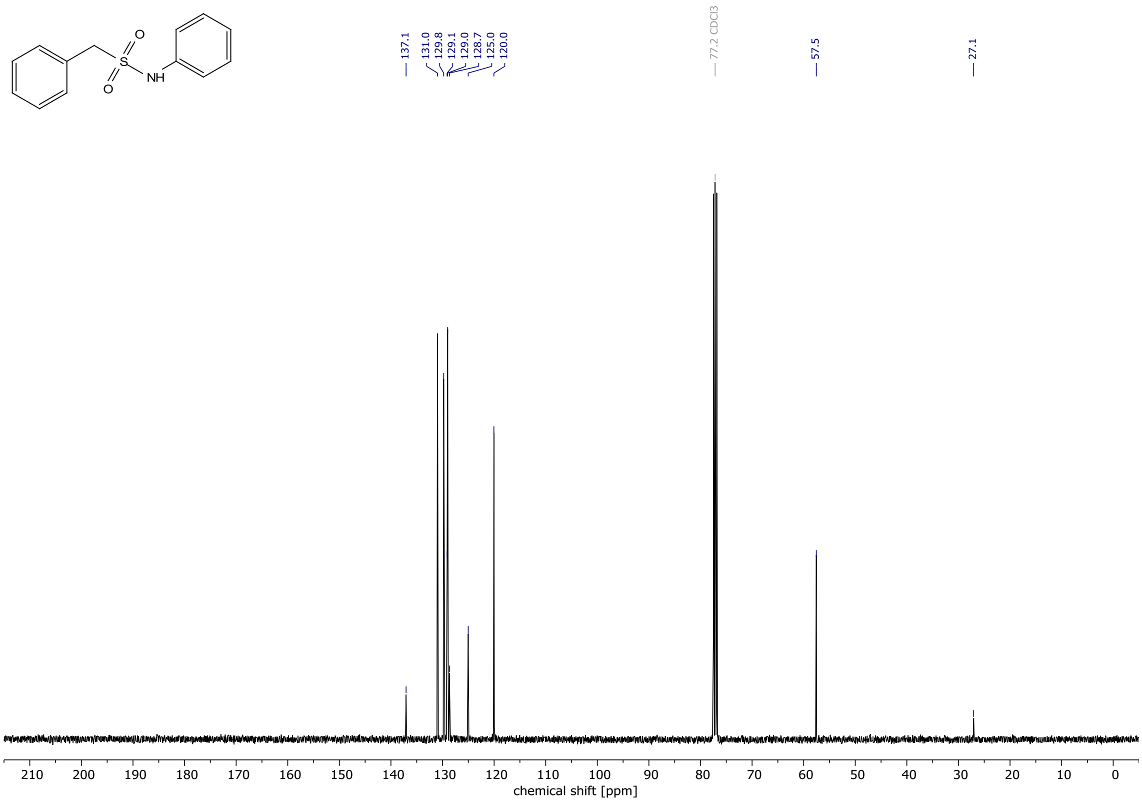

Figure S56: $^{13}C\{^{1}H\}$ NMR spectrum (101 MHz, 25 °C, $CDCl_3$) of **4k**.

Mass Spectra

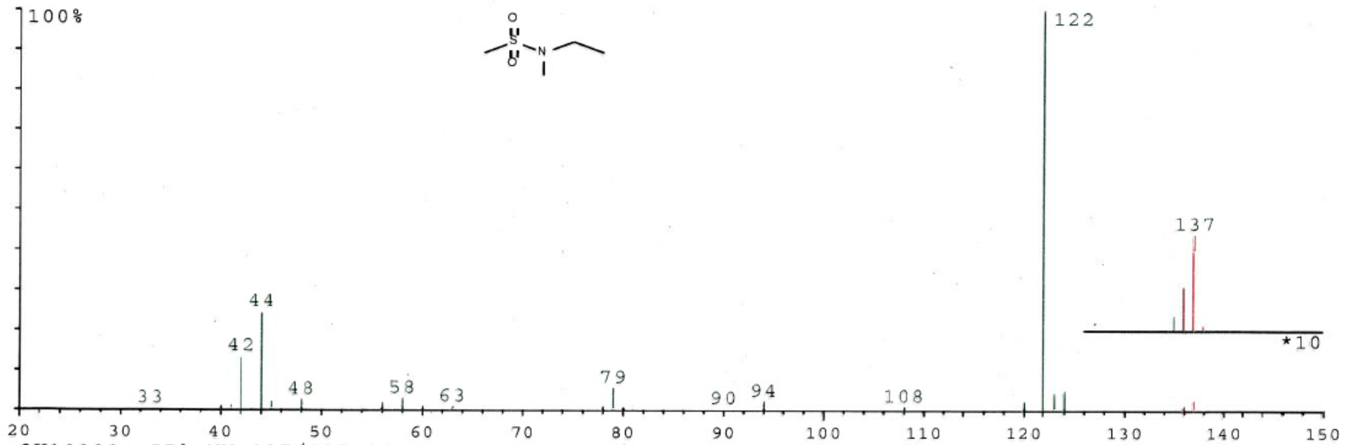

OU10222 57% MW:137/137.20 C4H11N1O2S1

33	0.03	65	0.05
35	0.01	78	0.70
38	0.06	79	5.30
40	0.68	80	0.44
41	0.87	81	0.34
42	13.09	90	0.18
43	0.38	94	2.00
44	24.20	106	0.39
45	1.80	108	0.64
46	0.24	111	0.01
48	2.31	120	1.90
52	0.02	122	100.00
54	0.22	123	4.05
55	0.11	124	4.55
56	1.58	125	0.14
57	0.16	135	0.38
58	2.68	136	1.11
60	0.58	137	2.37
62	0.03	138	0.14
63	0.64	139	0.01
64	0.07		

OU10222 57% MW:137/137.20 C4H11N1O2S1

Figure S57: Mass Spectrum of product **2g**.

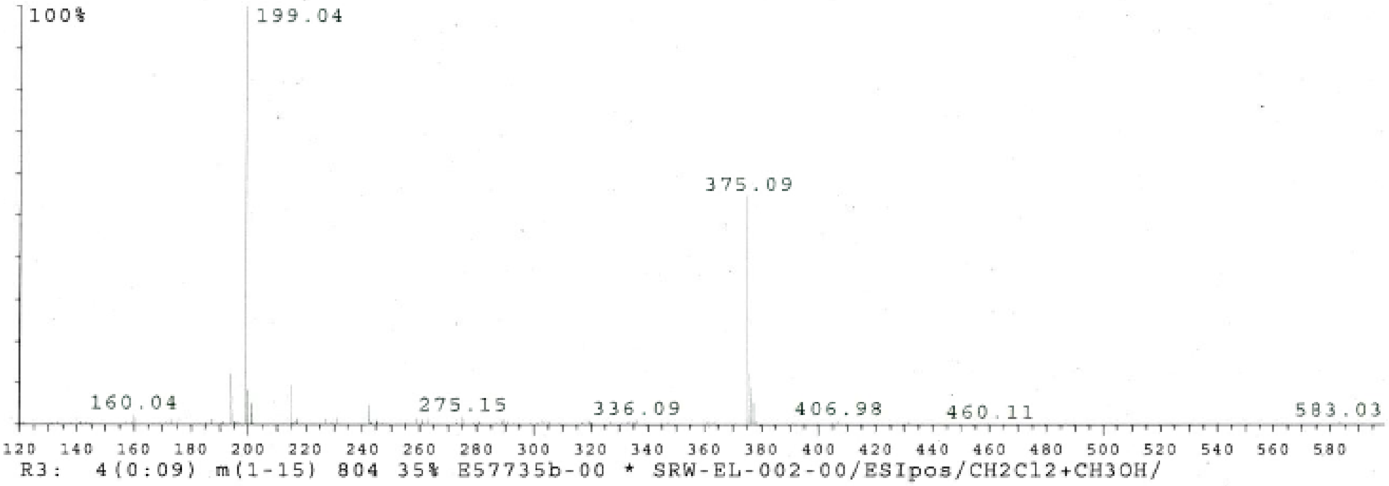

R3: 4(0:09) m(1-15) 804 35% E57735b-00 * SRW-EL-002-00/ESIpos/CH2Cl2+CH3OH/

Mass to be matched (m/z): 199.03999 charge: 1

Mass tolerance: ±0.005
restriction of atom numbers:

C	H	O	S	Na
1-100	1-100	0-10	1-1	1-1

Number of calculated formulas: 1

Formula	Diff. (ppm)	theor. m/z
C7 H12 Na1 O3 S1	-0.25	199.03994

Figure S58: Mass Spectrum of product **3d**.

Radical Trapping Experiment

Procedure:
The reaction was carried out using the undivided Teflon™ cells with a PTFE lid including the anode and a cathode as described in the General procedure GP2.

Electrolyte: An undivided Teflon™ cell is filled with methanesulfonic acid (3.54 mmol, 1 equiv.), tetrabutylammonium thiocyanate (0.5 mmol, 0.15 equiv.), 2,2,6,6-Tetramethylpiperidinyloxyl (TEMPO, 1.77 mmol, 1 equiv.) and acetonitrile (5 mL).

The lid including the electrodes is attached to the cell, the electric current and amount of applied charge are set to 172 mA (75 mA cm^{-2}), 3 *F* and the electrolysis was carried out at room temperature under constant stirring (900 rpm). After completion, the reaction mixture has been analyzed via GC-MS. No species other than TEMPO itself could be observed.

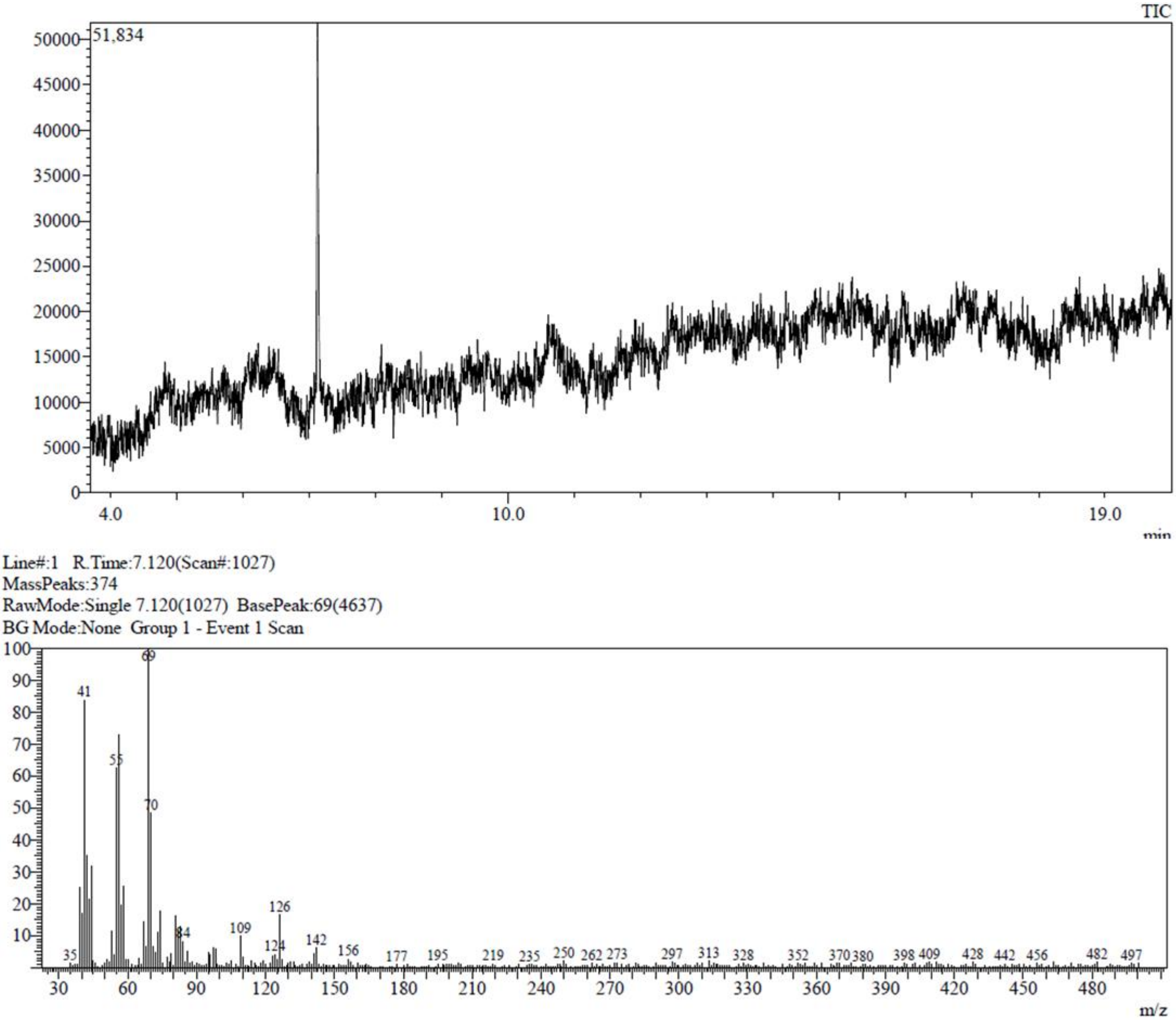

Figure S59: Result of the radical trapping experiment with TEMPO: No species other than TEMPO could be observed in GCMS.

Author contributions

**E.L.:**	conceptualization, investigation, methodology, data curation and analysis, writing the original draft.
**A.V.K.:**	supporting (part of the scope, cyclic voltammetry, scale up).
**J.S.:**	supporting (initial investigation, writing).
**S.R.W.:**	conceptualization, funding acquisition, project administration, resources, supervision, writing the original draft.

References

(1) Lowe, D. M.; Corbett, P. T.; Murray-Rust, P.; Glen, R. C. Chemical name to structure: OPSIN, an open source solution. *J. Chem. Inf. Model.* **2011**, *51* (3), 739–753. DOI: 10.1021/ci100384d

(2) Rundlöf, T.; Mathiasson, M.; Bekiroglu, S.; Hakkarainen, B.; Bowden, T.; Arvidsson, T. Survey and qualification of internal standards for quantification by 1H NMR spectroscopy. *J. Pharm. Biomed. Anal.* **2010**, *52* (5), 645–651. DOI: 10.1016/j.jpba.2010.02.007

(3) a) Izutsu, K. *Electrochemistry in Nonaqueous Solutions;* Wiley, 2002. DOI: 10.1002/3527600655#; b) Compton, R. G.; Banks, C. E. *Understanding voltammetry / Richard G Compton, Craig E Banks;* World Scientific, 2025; c) Elgrishi, N.; Rountree, K. J.; McCarthy, B. D.; Rountree, E. S.; Eisenhart, T. T.; Dempsey, J. L. A Practical Beginner's Guide to Cyclic Voltammetry. *J. Chem. Educ.* **2018**, *95* (2), 197–206. DOI: 10.1021/acs.jchemed.7b00361

(4) Gütz, C.; Klöckner, B.; Waldvogel, S. R. Electrochemical Screening for Electroorganic Synthesis. *Org. Process Res. Dev.* **2016**, *20* (1), 26–32. DOI: 10.1021/acs.oprd.5b00377

(5) Breitschaft, F. A.; Saak, A. L.; Krumbiegel, C.; Bartolomeu, A. d. A.; Weyhermüller, T.; Waldvogel, S. R. Multicomponent Electrosynthesis of Enaminyl Sulfonates Starting from Alkylamines, SO2, and Alcohols. *Org. Lett.* **2025**, *27* (5), 1210–1215. DOI: 10.1021/acs.orglett.4c04746

(6) Shi, W.; Bai, C.-M.; Zhu, K.; Cui, D.-M.; Zhang, C. Brønsted acid-assisted N-alkylation of sulfonamides using ethers as the alkylation reagents. *Tetrahedron* **2014**, *70* (2), 434–438. DOI: 10.1016/j.tet.2013.11.036

(7) Kloeckner, U.; Nachtsheim, B. J. Mild hypervalent iodine mediated oxidative nitration of N-aryl sulfonamides. *Chem. Commun.* **2014**, *50* (72), 10485–10487. DOI: 10.1039/C4CC04738A

(8) Tang, X.; Huang, L.; Qi, C.; Wu, X.; Wu, W.; Jiang, H. Copper-catalyzed sulfonamides formation from sodium sulfinates and amines. *Chem. Commun.* **2013**, *49* (54), 6102–6104. DOI: 10.1039/C3CC41249K

(9) Dɑbkowski, W.; Michalski, J.; Skrzypczyński, Z. A Convenient Synthesis Of Phosphorus And Sulfonyl Substituted N - Imidazoles (Triazoles) Using The Corresponding Acid Chlorides And N -Trimethylsilyl Imidazoles (Triazoles). *Phosphorus Sulfur Relat. Elem.* **1986**, *26* (3), 321–326. DOI: 10.1080/03086648608084586

(10) Varela-Fernández, A.; Varela, J. A.; Saá, C. Ruthenium-Catalyzed Cycloisomerization of Aromatic Homo- and Bis-Homopropargylic Amines/Amides: Formation of Indoles, Dihydroisoquinolines and Dihydroquinolines. *Adv. Synth. Catal.* **2011**, *353* (11-12), 1933–1937. DOI: 10.1002/adsc.201100095

(11) O'Sullivan, S.; Doni, E.; Tuttle, T.; Murphy, J. A. Metal-free reductive cleavage of C-N and S-N bonds by photoactivated electron transfer from a neutral organic donor. *Angew. Chem. Int. Ed.* **2014**, *53* (2), 474–478. DOI: 10.1002/anie.201306543

(12) Wilson, D. A.; Wilson, C. J.; Moldoveanu, C.; Resmerita, A.-M.; Corcoran, P.; Hoang, L. M.; Rosen, B. M.; Percec, V. Neopentylglycolborylation of aryl mesylates and tosylates catalyzed by Ni-based mixed-ligand systems activated with Zn. *J. Am. Chem. Soc.* **2010**, *132* (6), 1800–1801. DOI: 10.1021/ja910808x

(13) Albrecht, S.; Defoin, A.; Tarnus, C. Simple Preparation of O-Substituted Hydroxylamines from Alcohols. *Synthesis* **2006**, *2006* (10), 1635–1638. DOI: 10.1055/s-2006-926440

(14) Kuhne, S.; Kooistra, A. J.; Bosma, R.; Bortolato, A.; Wijtmans, M.; Vischer, H. F.; Mason, J. S.; Graaf, C. de; Esch, I. J. P. de; Leurs, R. Identification of Ligand Binding Hot Spots of the Histamine H1 Receptor following Structure-Based Fragment Optimization. *J. Med. Chem.* **2016**, *59* (19), 9047–9061. DOI: 10.1021/acs.jmedchem.6b00981

(15) Curti, C.; Zanardi, F.; Battistini, L.; Sartori, A.; Rassu, G.; Pinna, L.; Casiraghi, G. Streamlined, asymmetric synthesis of 8,4'-oxyneolignans. *J. Org. Chem.* **2006**, *71* (22), 8552–8558. DOI: 10.1021/jo061521t

(16) Laudadio, G.; Barmpoutsis, E.; Schotten, C.; Struik, L.; Govaerts, S.; Browne, D. L.; Noël, T. Sulfonamide Synthesis through Electrochemical Oxidative Coupling of Amines and Thiols. *J. Am. Chem. Soc.* **2019**, *141* (14), 5664–5668. DOI: 10.1021/jacs.9b02266

(17) Hu, L.; Liu, X.; Liao, X. Nickel-Catalyzed Methylation of Aryl Halides with Deuterated Methyl Iodide. *Angew. Chem. Int. Ed.* **2016**, *55* (33), 9743–9747. DOI: 10.1002/anie.201604406

(18) Chen, J.; Han, X.; Mei, L.; Liu, J.; Du, K.; Cao, T.; Li, Q. Iodine-catalyzed sulfonylation of sulfonyl hydrazides with tert-amines: a green and efficient protocol for the synthesis of sulfonamides. *RSC Adv.* **2019**, *9* (54), 31212–31216. DOI: 10.1039/C9RA07361B

(19) Moon, S.-Y.; Nam, J.; Rathwell, K.; Kim, W.-S. Copper-catalyzed Chan-Lam coupling between sulfonyl azides and boronic acids at room temperature. *Org. Lett.* **2014**, *16* (2), 338–341. DOI: 10.1021/ol403717f

(20) Tan, B. Y.-H.; Teo, Y.-C.; Seow, A.-H. Low Catalyst Loadings for Ligand-Free Copper(I)-Oxide-Catalyzed N -Arylation of Methanesulfonamide in Water. *Eur. J. Org. Chem.* **2014**, *2014* (7), 1541–1546. DOI: 10.1002/ejoc.201301561

(21) Holden, C. M.; Sohel, S. M. A.; Greaney, M. F. Metal Free Bi(hetero)aryl Synthesis: A Benzyne Truce–Smiles Rearrangement. *Angew. Chem.* **2016**, *128* (7), 2496–2499. DOI: 10.1002/ange.201510236

(22) García Ruano, J.; Parra, A.; Yuste, F.; Mastranzo, V. Mild and General Method for the Synthesis of Sulfonamides. *Synthesis* **2008**, *2008* (2), 311–319. DOI: 10.1055/s-2007-1000850

(23) Hill, B.; Liu, Y.; Taylor, S. D. Synthesis of alpha-fluorosulfonamides by electrophilic fluorination. *Org. Lett.* **2004**, *6* (23), 4285–4288. DOI: 10.1021/ol048249z
